# Scaffolds in the microbial resistant era: Fabrication, materials, properties and tissue engineering applications

**DOI:** 10.1016/j.mtbio.2022.100412

**Published:** 2022-08-30

**Authors:** Ángel Serrano-Aroca, Alba Cano-Vicent, Roser Sabater i Serra, Mohamed El-Tanani, AlaaAA. Aljabali, Murtaza M. Tambuwala, Yogendra Kumar Mishra

**Affiliations:** a Biomaterials and Bioengineering Lab, Centro de Investigación Traslacional San Alberto Magno, Universidad Católica de Valencia San Vicente Mártir, C/Guillem de Castro 94, 46001, Valencia, Spain; b Centre for Biomaterials and Tissue Engineering, Universitat Politècnica de València, 46022, València, Spain; c Pharmacological and Diagnostic Research Centre, Faculty of Pharmacy, Al-Ahliyya Amman University, Amman, 19328, Jordan; d Department of Pharmaceutics and Pharmaceutical Technology, Yarmouk University, Irbid, 21163, Jordan; e School of Pharmacy and Pharmaceutical Science, Ulster University, Coleraine, BT52 1SA, UK; f Mads Clausen Institute, NanoSYD, University of Southern Denmark, Alsion 2, 6400, Sønderborg, Denmark

**Keywords:** Biomaterials, Scaffolds, Antimicrobial activity, Tissue engineering, Fabrication

## Abstract

Due to microbial infections dramatically affect cell survival and increase the risk of implant failure, scaffolds produced with antimicrobial materials are now much more likely to be successful. Multidrug-resistant infections without suitable prevention strategies are increasing at an alarming rate. The ability of cells to organize, develop, differentiate, produce a functioning extracellular matrix (ECM) and create new functional tissue can all be controlled by careful control of the extracellular microenvironment. This review covers the present state of advanced strategies to develop scaffolds with antimicrobial properties for bone, oral tissue, skin, muscle, nerve, trachea, cardiac and other tissue engineering applications. The review focuses on the development of antimicrobial scaffolds against bacteria and fungi using a wide range of materials, including polymers, biopolymers, glass, ceramics and antimicrobials agents such as antibiotics, antiseptics, antimicrobial polymers, peptides, metals, carbon nanomaterials, combinatorial strategies, and includes discussions on the antimicrobial mechanisms involved in these antimicrobial approaches. The toxicological aspects of these advanced scaffolds are also analyzed to ensure future technological transfer to clinics. The main antimicrobial methods of characterizing scaffolds’ antimicrobial and antibiofilm properties are described. The production methods of these porous supports, such as electrospinning, phase separation, gas foaming, the porogen method, polymerization in solution, fiber mesh coating, self-assembly, membrane lamination, freeze drying, 3D printing and bioprinting, among others, are also included in this article. These important advances in antimicrobial materials-based scaffolds for regenerative medicine offer many new promising avenues to the material design and tissue-engineering communities.

## Highlights

• Antibacterial, antifungal and antibiofilm scaffolds.
• Antimicrobial scaffold fabrication techniques.
• Antimicrobial biomaterials for tissue engineering applications.
• Antimicrobial characterization methods of scaffolds.
• Bone, oral tissue, skin, muscle, nerve, trachea, cardiac, among other applications.

## Graphical abstract

**Figure:**
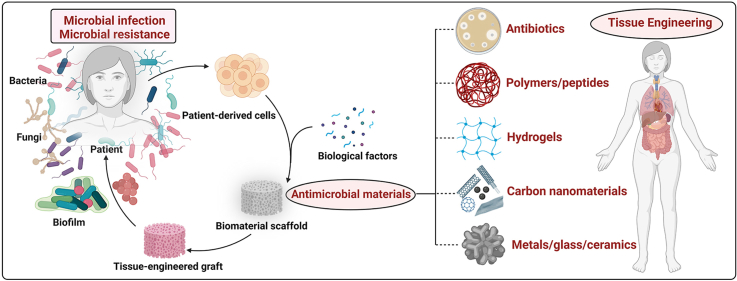

## 1 Introduction to tissue engineering and microbial resistance

**Fig. 1:**
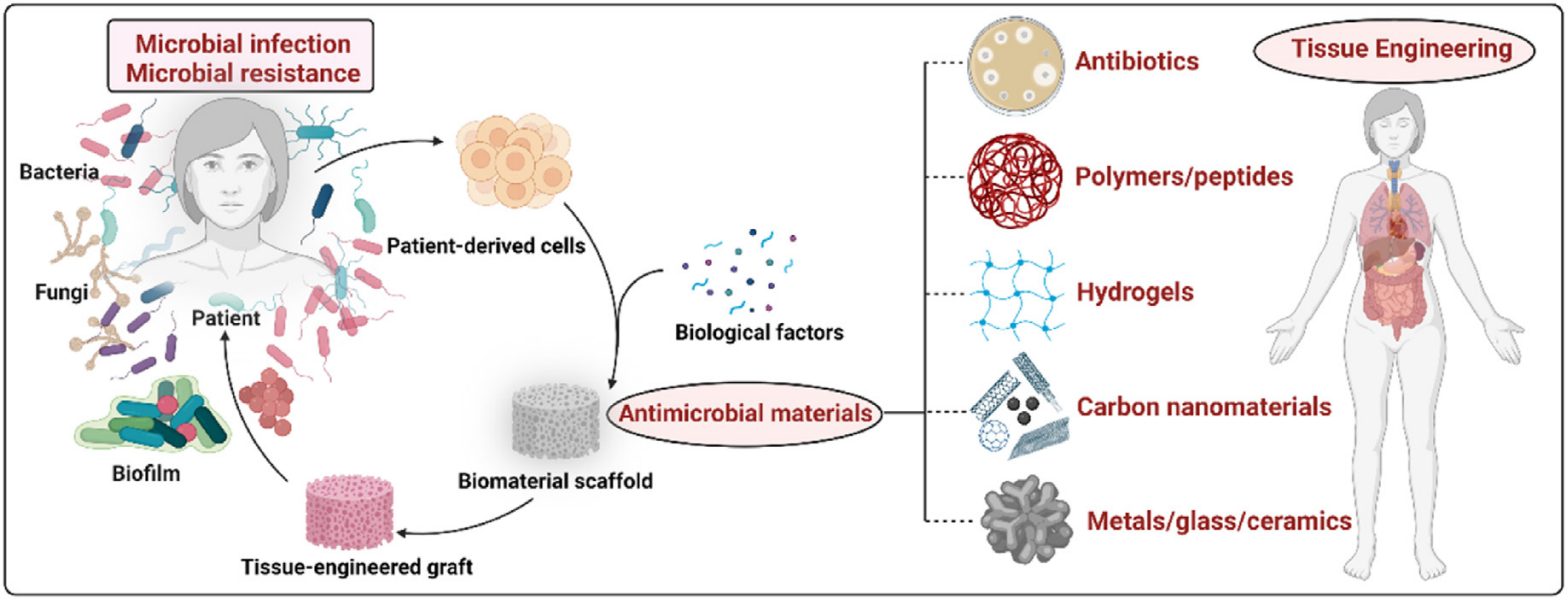
Antimicrobial scaffolds to prevent microbial infections in tissue engineering applications. Created with Biorender by Ángel Serrano-Aroca.

Tissue engineering is currently attempting to provide breakthrough technologies capable of achieving successful results in regenerative medicine [1]. The tissue engineering regeneration strategy relies on the creation of biomimetic 3D cellular microenvironments (artificial ECM or scaffolds) that control and guide local tissue regeneration, usually made from a combination of natural and/or synthetic biodegradable biomaterials, cells and biomolecules (bioactive factors) [2]. The natural and synthetic polymers commonly used in tissue engineering include chitosan, alginate, gelatin, agarose, collagen, hyaluronic acid, carrageenan (CG), polycaprolactone (PCL), polyvinyl alcohol (PVA), polylactic acid (PLA), polyglycolide acid (PGA), poly lactic-co-glycolic acid (PLGA), poly (hydroxybutyrate-co-valerate) (PHBV) and many others [[3], [4], [5], [6], [7], [8], [9]]. Depending on the type of application, the scaffold will require specific physical-chemical (biodegradability, mechanical properties, etc.) and morphological properties (surface topology, pore size, pore distribution and interconnection, etc.) to mimic the cellular environment *in vivo* [10]. Most of the degradable polymers used to produce scaffolds can also release biomolecules that promote tissue regeneration, including growth factors, or antimicrobials to fight infections. The ability to manipulate physical-chemical variables (cross-linking, blends, copolymerization, etc.) enables the release dynamics to be tailored to the requirements of the application [11,12]. However, antibiotic resistance in pathogenic microorganisms has reached alarming levels and has become a serious global public health problem [13]. The use of alternative antimicrobial agents capable of dealing with antibiotic-resistant bacteria such as metal ions [[14], [15], [16]], quaternary ammonium compounds [17,18], antimicrobial peptides [19], peptoids [20], α-peptides [21], β-peptides [22], carbon-based nanomaterials [[23], [24], [25]] or combined strategies [26,27] are being given a lot of attention by researchers for their important contributions to future healthcare systems. Growth factors are often studied in cell-free tissue-engineering approaches to facilitate tissue regeneration [28]. However, their use can generate problems associated with immunogenicity, cancer risk and associated problems in cellular homeostasis [29,30]. In this context, the use of inorganic biomolecules is being studied for regeneration applications since they induce tissue regeneration without the drawbacks of growth factors [30,31]. Biometals have shown potential results in regenerative medicine, mostly because of their affordability, stability, and capacity to trigger cellular responses *via* signaling pathways. Biometals like zinc integrated into scaffolds are also being researched as regenerative agents [[32], [33], [34], [35], [36]] Their antibacterial qualities give them additional advantages for the prevention of infections following scaffold implantations. Biomolecules with both bioactivity and antibacterial characteristics have thus been the basis of newly discovered methods for regenerative medicine applications [[37], [38], [39]]. Some of the biomaterials used as scaffolds for tissue engineering (with no additional components) also possess intrinsic antimicrobial properties, providing a cellular microenvironment capable of stimulating cellular response and simultaneously inhibiting microbial growth [[40], [41], [42]] (Fig. 1).

Since surgical infections in tissue engineering are associated with significant postoperative morbidity, increased healthcare costs and high risk of death in case of multidrug-resistant pathogens, the scientific community have been working hard on the development of antimicrobial scaffolds for the last ten years. This article reviews the current state of antimicrobial scaffolds produced for bone, oral tissue, skin, muscle, nerve, trachea, cardiac and other tissue engineering applications. The latest scaffolds developed to prevent infections produced by bacteria and fungi are also discussed in depth with detailed descriptions.

## 2 Production strategies for antimicrobial scaffolds

**Fig. 2:**
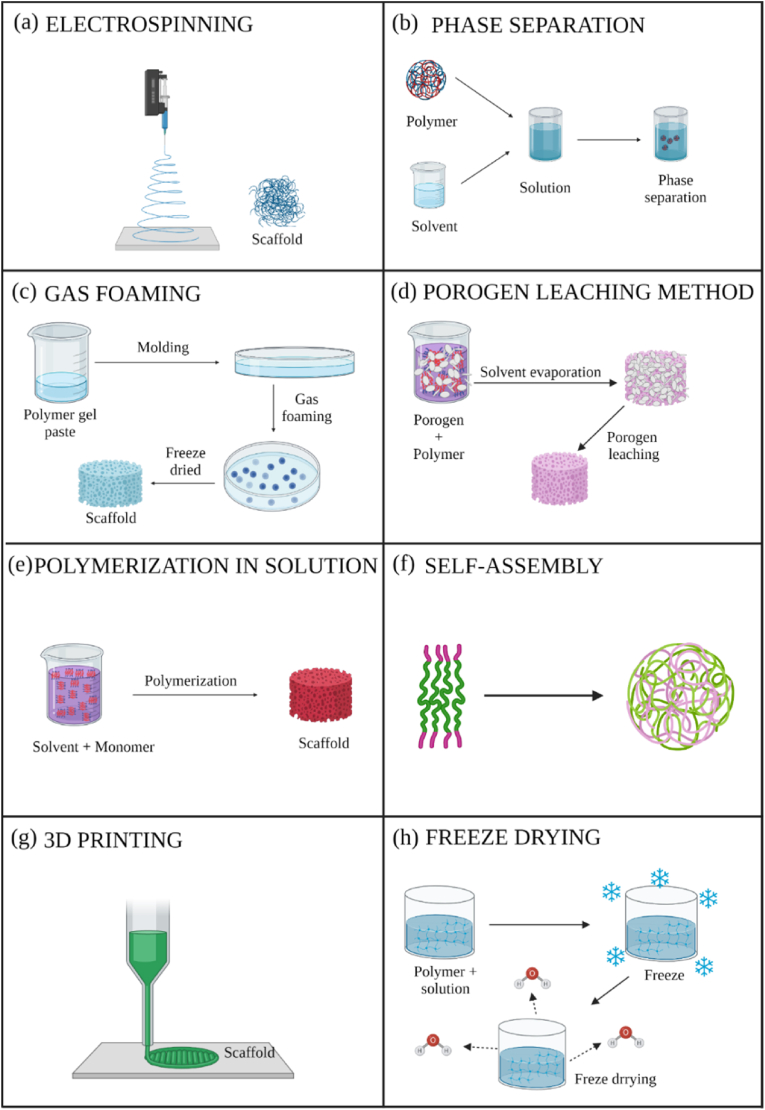
Production methods for antimicrobial scaffolds: (a) electrospinning; (b) phase separation; (c) gas foaming; (d) porogen leaching method; (e) polymerization in solution; (f) self-assembly; (g) 3D printing; (h) freeze drying.

Many production techniques have been developed for porous materials to be used as scaffolds in tissue engineering applications, such as electrospinning [3,8], phase separation [43,44], gas foaming [45,46], porogen method [[47], [48], [49]], polymerization in solution [[50], [51], [52], [53], [54]], fiber mesh coating [55,56], self-assembly [57,58], membrane lamination [59,60], freeze drying [1,61,62], 3D-printing [[63], [64], [65]] and bioprinting [66], among others [67]. These methods require the use or introduction of materials with intrinsic antimicrobial activity as fillers to produce antimicrobial scaffolds. The main scaffold production methods, such as electrospinning, phase separation, gas foaming, porogen leaching, polymerization in solution, self-assembly, 3D printing and freeze drying, are shown in Fig. 2.

**Fig. 3:**
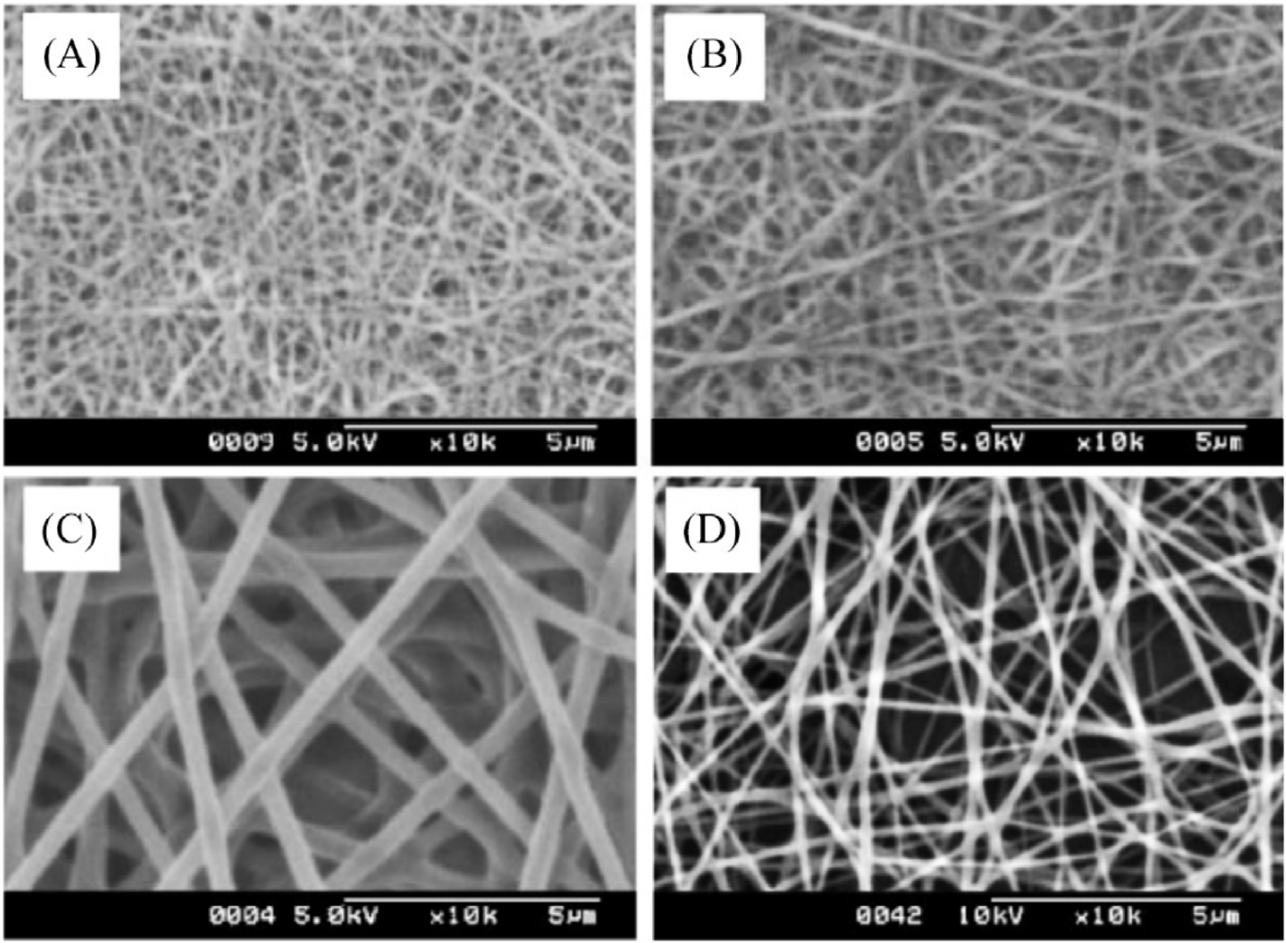
Scanning electron microscope images of chitosan and PVA blended electrospun fibers at a magnification of x10000. Chitosan and PVA were dissolved in formic acid at 7% *w/w* and in distilled water at 9% *w/w*, respectively. The two solutions were mixed and electrospun in the indicated chitosan: PVA specified volume ratios of 50:50 (A), 30:70 (B) and 0:100 (C). Electrospun fibers made of a mixture of chitosan dissolved in formic acid (or 0.2 ​M acetic acid) at 2% *w/w* and mixed with a solution of 9% *w/w* PVA in a volume ratio of 50:50 (D). Adapted with permission from Ref. [69]. Copyright 2004 Elsevier.

Electrospinning uses polymers for scaffold design, generating polymeric fibers controlled by an electric field between two electrodes [3,8] to produce porous substrates made of ultra-fine fibers with a large surface area, which makes them ideal environments for cell growth and subsequent tissue organization [68], e.g. antimicrobial scaffolds made of polymers with intrinsic antimicrobial activity such as chitosan (CS) (Fig. 3) [69,70].

The phase separation scaffold production technique is based on separating the polymeric solution into two phases by temperature changes [43]. The polymer is dissolved in a solvent to produce porous scaffolds with bioactive molecules integrated into that structure after evaporation and sublimation [44]. Phase separation can be combined with other techniques to design 3D structures with a controlled pore morphology [71]. This technique is widely used to make polymer-based scaffolds such as PLA for regenerative medicine applications [72]. The polymeric matrix can be combined with other materials with intrinsic antimicrobial properties.

The great advantage of gas foam scaffolding manufacturing techniques is that they do not require chemicals or high temperatures, which can damage cells, tissue and the microenvironment [43]. Nucleation of pores is created due to gas phase separation from the polymer, expanding the scaffold volume while reducing the polymer density [45]. The gas foaming method is often used to produce new nanocomposite scaffolds charged with a material with antimicrobial properties [46].

The porogen leaching method is commonly used to produce scaffolds with the required geometry, pore size and pore interconnection using a porogen such as salt, wax, sugar, polymers, glass, fibers, polymer microspheres, meshes, etc. [47,48,73,74]. A porogen of the desired size and shape is leached away from the polymer mixture by a suitable solvent to make the scaffold with the required characteristics [75]. This technique is often used in combination with melt molding [[76], [77], [78]] to produce degradable polymer scaffolds, which are the basis of many new developments in antimicrobial scaffolds.

Scaffolds can also be produced *via* polymerization in the presence of a solvent, e.g. poly (methyl methacrylate) (PMMA) sponges can be made *via* polymerization in solution with ethanol [51,52,79]. Poly (2-hydroxyethyl acrylate) (PHEA) porous hydrophilic sponges have also been created by polymerization in solution in the presence of water, ethanol or methanol [50,53,80]. Hybrid PHEA/PMMA sponges can be obtained by combining polymerization in solution with plasma polymerization [[81], [82], [83]]. This means antimicrobial scaffolds could be synthetized by polymerization in solution combined with the incorporation of antimicrobial nanomaterials such as graphene oxide (GO) [84].

The self-assembly technique is based on the spontaneous organization of several molecules in a given medium, forming an ordered structure with a specific function [57]. It commonly used, for example, in amphiphilic peptides in aqueous solution that link their hydrophobic residues through non-covalent bonds [85], forming 3D nanofibers for tissue engineering [58].

The membrane lamination method is used to construct layer by layer anatomically accurate three-dimensional scaffold assemblies during the manufacturing process [59,60]. The fiber mesh coating method consists of depositing a polymer solution on a porous polymer fiber mesh and subsequently allowing the solvent to evaporate [55,56].

The freeze dying technique is based on the sublimation principle and is used to manufacture porous scaffolds for tissue engineering [1,61,62]. Scaffolds with high porosity can be produced by dissolving a polymer in a solvent. After freezing the mixture, the solvent is removed by lyophilization [86]. This technique is simple and can manufacture highly porous scaffolds of a certain pore size, which are determining factors in tissue engineering [87].

**Fig. 4:**
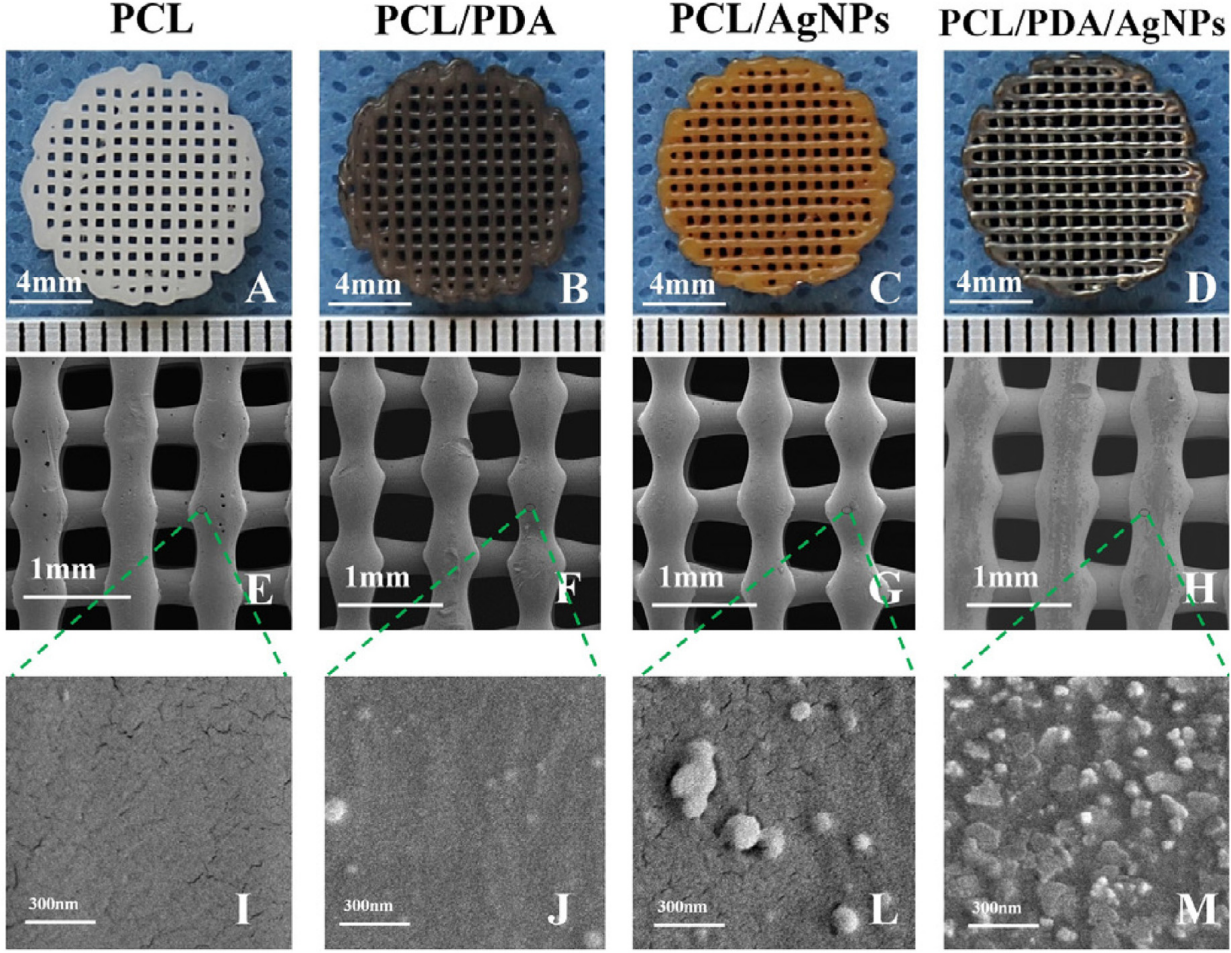
Fused deposition modeling 3D-printing scaffolds for bone tissue regeneration: morphology and surface microstructure. Scaffold images of PCL (A), PCL/PDA (B), PCL/AgNPs (C), PCL/PDA/AgNPs (D). Scanning electron microscopy photographs of PCL (E, I), PCL/PDA (F, J), PCL/AgNPs (G, L), PCL/PDA/AgNPs (H, M) scaffolds. Reprinted with permission from Ref. [63]. Copyright 2019 Elsevier.

Additive manufacturing (AM) of 3D printed scaffolds is a highly reproducible method, as it can produce computer-controlled 3D porous materials [8]. The previous design of scaffold models is required by advanced computer-aided design [64,65]. The AM techniques available to create scaffolds include fused deposition modeling (FDM) [88], selective laser sintering (SLS) and stereolithography [8], among many others, e.g. antimicrobial 3D printed dual-functional PCL-based biomaterial scaffolds with self-assembly micro-nano surface, polydopamine (PDA) and enriched nano argentumo as silver nanoparticles (AgNPs) (abbreviated to PCL/PDA/AgNPs) have been made by FDM (Fig. 4) [63].

These scaffolds not only showed good antibacterial and cytocompatibility results *in vitro*, but also performed well in an *in vivo* rabbit model, demonstrating their potential for bone regeneration due to their compatibility, antimicrobial capacity and mechanical properties [63]. The production of porous metal alloys with powerful antimicrobial properties by AM for potential biomedical applications has recently been reported [89,90].

The reproducible, automatic 3D bioprinting technique uses biomaterials, cells and growth factors to produce artificial living tissues or even an entire organ [10,91,92]. Multicellular building blocks (bioinks) are distributed layer by layer and scaled to manufacture the final construct [10].

**Fig. 5:**
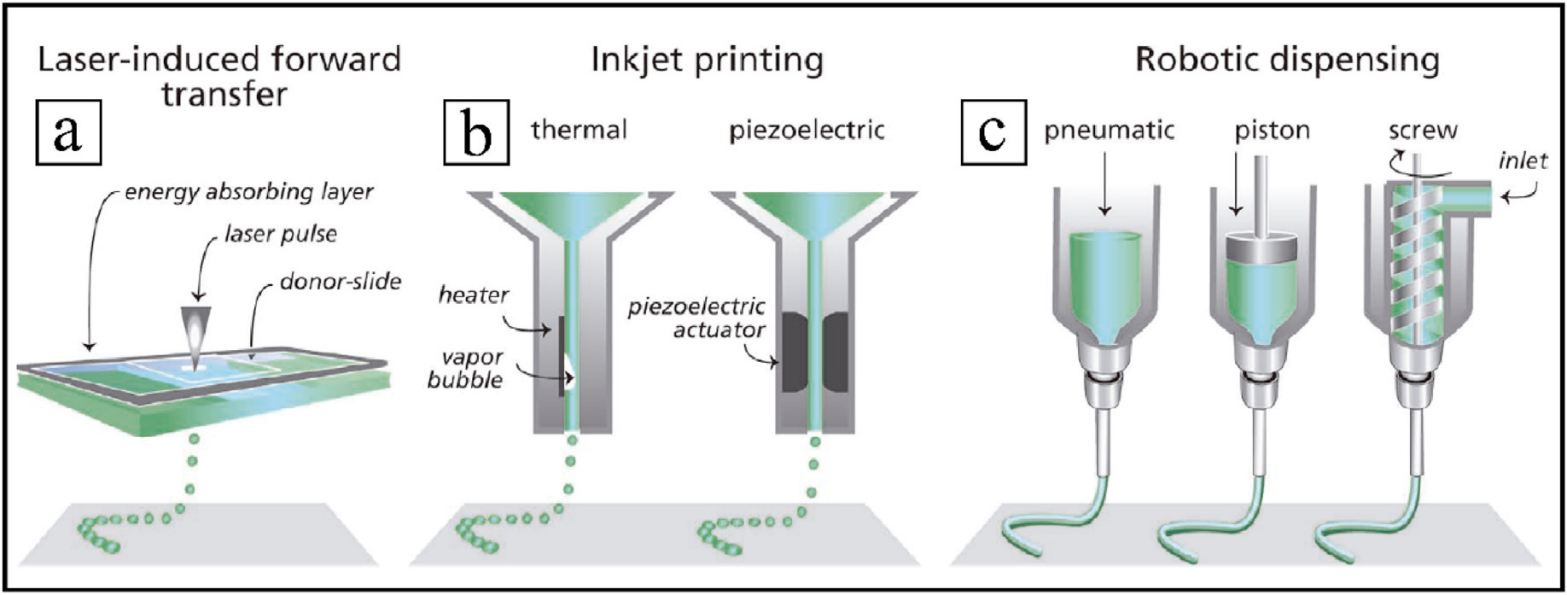
Main bioprinting technological methods: laser-induced forward transfer (a), inkjet printing (b) and robotic dispensing (c). Adapted with permission from Ref. [66]. Copyright 2013 John Wiley and Sons.

Bioprinting includes a number of different methods: laser-induced forward transfer, inkjet printing, or robotic dispensing [66] (Fig. 5), with specific requisites for bioinks. Bioprinting aims to engineer solid organs by computer-controlled systems capable of depositing biomaterials with or without cells to create solid and viable organs. However, the diversity of solid organs in terms of specific cellular and structural microenvironments, together with the demands of nutrients, is still a challenge [93,94]. Different types of tissue approaches have recently been investigated, such as blood vessels [95,96], skin [97,98], cardiac tissue [99,100], bladder and urethral tissue [10,101], cartilage [96,102] or bone [102,103], among others.

We firmly believe that bioprinting is a rapidly emerging technology that will provide a real clinical solution to the shortage of organ donors while avoiding the associated risks of transmitting diseases and immunological rejection. This method will certainly revolutionize the biomedical sector in the near future. Also, if the bioprinting design is performed with the additional aim of incorporating antimicrobial agents, the chances of success of the engineered constructs increase exponentially. A variety of antimicrobial materials can thus be used to enhance the current bioink formulations to improve biocompatibility and combat the spread of multidrug-resistant infections [104,105].

## 3 Antimicrobial scaffolds for tissue engineering

Tissue engineering has undoubtedly become a promising strategy for repairing damaged or diseased tissue [3] by means of highly porous materials or scaffolds capable of providing structural support for the engineered cellular environment with rapid diffusion of nutrients and metabolites [106]. If these scaffolds also incorporate new antimicrobial materials that can prevent microbial infections they will be even more promising. Controlled drug delivery materials, medical prostheses and medical devices are examples of other biomedical applications [3]. Enormous progress has been made in material engineering and the design of biomaterials that can mimic ECM [107]. Many preclinical and clinical trial studies have looked into the effect of stem cell-based therapies for tissue regeneration [108,109]. For example, human induced pluripotent stem cells are a powerful tool for the generation of specialized cells to treat diseases such as nonalcoholic steatohepatitis (NASH) [110]. Mesenchymal stem cells (MSCs) are gaining a lot of interest as perfect candidates for cell therapy and tissue engineering, due to their ability to differentiate into different cell types [111,112]. However, their potential in bioengineering is reduced when the reactive oxygen and nitrogen species levels overcome the physiological levels, which can worsen differentiation and proliferation while it favors senescence and cell death [113]. In this regard, nano-antioxidants in the form of chemical compounds, biometabolites, or protein precursors/proteins are effective in the treatment of MSCs to optimize their clinical use.

**Fig. 6:**
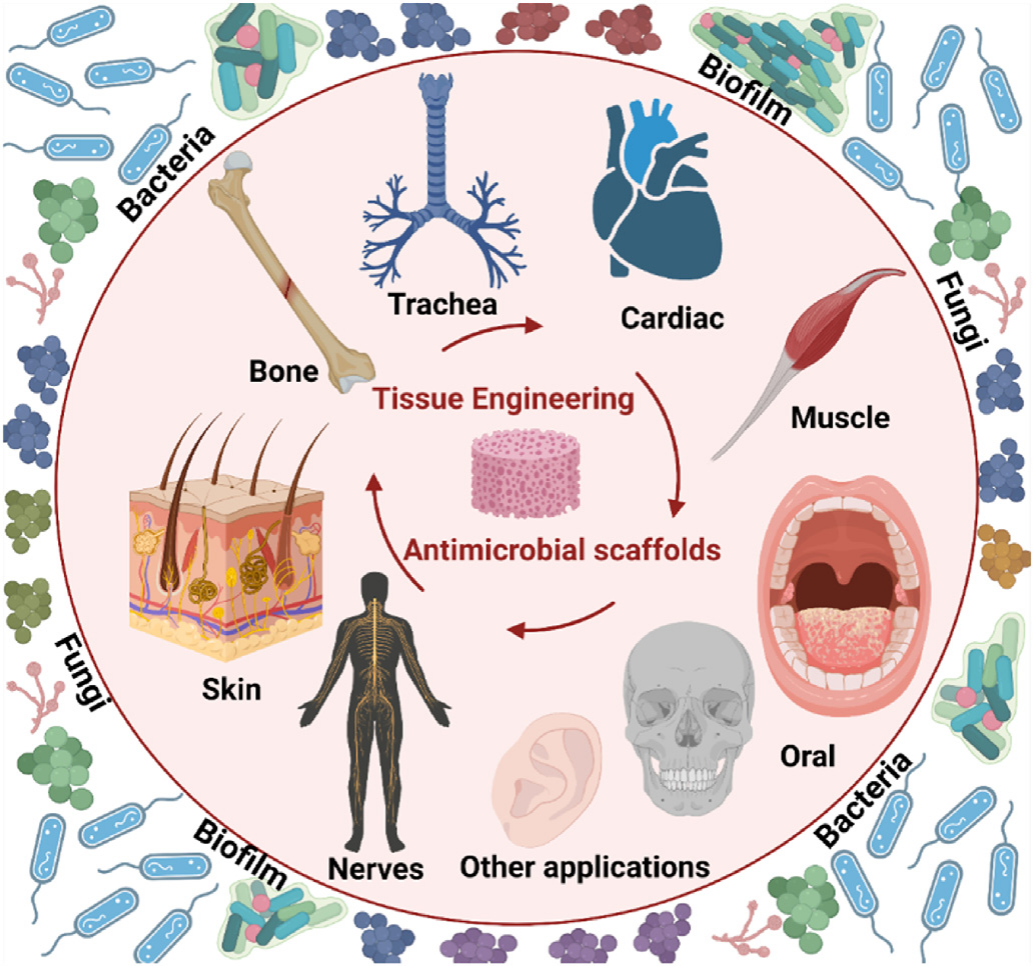
Tissue engineering application fields for antibacterial, antifungal and antibiofilm scaffolds. Created with Biorender by Ángel Serrano-Aroca.

Biomaterials used as scaffolds for tissue engineering are preferably endowed with antimicrobial intrinsic or extrinsic agents to provide a 3D environment with bioactive and biocidal properties. This section describes a broad range of current antibacterial, antifungal and antibiofilm scaffolds according to their specific tissue engineering application, such as bone, oral tissue, muscle, nerve, trachea, cardiac, and skin, among others (Fig. 6).

### 3.1 Antimicrobial fillers

Scaffolds containing antimicrobial fillers such as antibiotics, antiseptics, polymers, peptides, carbon nanomaterials, metals, ceramics or combined and alternative strategies have been developed to prevent and/or treat infections in tissue engineering. For example, polyhydroxyalkanoate/chitosan (PHA/CS) and 2D molybdenum disulfide-doped (2D MoS_2_) scaffolds have been proposed for biomedical and antimicrobial applications such as wound healing and antibacterial treatment of skin infections caused by methicillin-resistant *Staphylococcus aureus* (MRSA) [114]. These materials are biocompatible and also show promise for drug delivery. Other approaches consisted of creating PCL nanofibers containing Ag nanoparticles by electrospinning to produce antibacterial scaffolds [115]. Cell viability studies on this material have revealed that cytotoxicity is highly dependent on the concentration of silver nanoparticles. Brennan et at. evaluated the degradation products resulting from the acid digestion of scaffolds composed of ECM for antibacterial effects against *Staphylococcus aureus* and *Escherichia coli*. The results suggest that several low-molecular-weight peptides with antibacterial activity exist within the ECM, which may help explain the resistance to bacterial infection provided by these biobased scaffolds [116]. Biocompatible boron nitride doped polyhydroxyalkanoate/chitosan (PHA/Ch-hBN) nanocomposite scaffolds have been successfully designed and manufactured with superior antibacterial activity by means of the solvent casting technique [117]. In another study, CS-blended PLA nanofibers were successfully produced by electrospinning [118]. CS-blended PLA nanofibers exhibited antibacterial activity against *E. coli* and no cytotoxicity in mouse fibroblasts (L929 ​cell line), making them potential candidates for biomedical applications. Many types of antibacterial scaffolds that are safe and efficient for biological applications have thus been produced containing different types of antimicrobial fillers to provide the required characteristics for specific tissue engineering applications.

### 3.2 Antibacterial scaffolds for bone regeneration

The most widely used practice to treat large bone defects has been autologous bone grafting [119]. Unfortunately, this strategy is associated with the morbidity of the donor site, the need for one or more surgical interventions and the small amount of bone that can be removed from the patient [120]. A lot of research has been done to make bone substitutes that are structurally and functionally similar to real bone, such as highly porous 3D scaffolds that help to achieve the diffusion of nutrients and metabolites and antibacterial activity following a broad range of strategies (see Table 1).

**Table 1:** Antibacterial scaffolds for bone regeneration: scaffolds with antibiotics, polymers, peptides, carbon nanomaterials, metals, combined and alternative strategies.

Material	Fabrication method	Bacteria	Non-toxicity: cell line/animal model	Year	Ref
**Scaffolds with antibiotics**					
Gentamicin-contained PCL-HAp composite scaffold	Electrospinning	*E. coli*	Not studied	2013	[121]
CPFX loaded gelatin-HAp scaffolds	Freeze drying	*S. aureus (MRSA)*	Adipose derived MSCs	2015	[122]
Bioactive glass, PVA, several antibiotics	Rapid prototyping	*E. coli and S. aureus*	MC3T3-E1 preosteoblast cells	2017	[123]
Baghdadite-vancomycin scaffolds	Space holder method	*S. aureus*	MG-63 osteoblast cells	2017	[124]
Vancomycin-laden mesoporous bioglass/poly (lactic-*co*-glycolic acid) composite scaffolds	Freeze-drying	*S. aureus*	Human BMSCs	2018	[125]
Poly (ε-Caprolactone) composite scaffolds with vancomycin-loaded polylactic acid-glycolic acid	3D printing	*S. aureus*	Rabbit bone MSCs	2018	[126]
Levofloxacin hydrochloride-loaded mesoporous silica microspheres on nano HAp/PU	*In situ* foaming method	*E. coli and S. aureus*	L929 mouse fibroblast cells/Rabbit	2019	[127]
Macroporous agarose/nHCA scaffolds containing VEGF and cephalexin	3D printing (GELPOR3D method)	*S. aureus*	MC3T3-E1 preosteoblast cells	2019	[128]
Polylactic acid-collagen-minocycline-nano HAp	3D printing	*S. aureus*	hMSCs	2019	[37]
Polyetheretherketone/polyglycolide acid scaffolds with total alkaloids from semen strychnine	3D printing	*E. coli and S. aureus*	hFOB1.19 osteoblast cells	2020	[129]
HA-SA–CS–VEGF and vancomycin	Microspheres-freeze drying	*S. aureus*	BMSCs	2021	[130]
Laponite nanoplates/amoxicillin-functionalized PLA nanofibrous scaffolds	Electrospinning	*E. coli and S. aureus*	hBMSCs	2022	[131]
**Scaffolds with antibacterial polymers/peptides**					
PCL/CS nanofibers with oligopeptides	Electrospinning	*S. epidermidis*	hFOB1.19 osteoblast cells	2013	[132]
O-Acrylamidomethyl-2-hydroxypropyltrimethyl ammonium chloride CS and silk modified mesoporous bioactive glass scaffolds	Surface modification	*S. epidermis and S. aureus*	hMSCs	2016	[133]
HACC-grafted PLGA/HAp scaffolds	3D printing	*S. aureus*	Rat and Rabbit	2018	[134]
PCL/PDA/AgNPs scaffold	3D printing	*S. aureus*	Rabbit BMSCs/Rabbit	2018	[63]
PLA-gelatin-nano HAp with ponericin	3D printing	*E. coli and S. aureus*	MC3T3-E1 preosteoblast cells	2018	[135]
nHA-starch-alginate/chitosan scaffolds	S-nitroso-N-acetyl-penicillamine (SNAP) as the NO donor	*S. aureus and P. aeruginosa*	3T3 mouse fibroblast cells	2019	[136]
Collagen-PLGA microspheres-synthetic peptide	Electrospray and freeze-drying	*E. coli and S. aureus*	MC3T3-E1 preosteoblast cells	2020	[137]
EPL/PCL/HAp scaffolds	3D printing	*S. aureus, E. coli and S. mutans*	MC3T3-E1 preosteoblast cells	2020	[138]
Chitosan-vanillin-bioglass	Freeze drying	*S. gordonii and S. Sanguinis*	MC3T3-E1 preosteoblast cells	2021	[139]
Antibacterial peptide-modified Silk fibroin and silica NPs	Micro-extrusion 3D printing and directional freeze-casting/drying approaches	*E. coli and S. aureus*	MC3T3-E1 preosteoblast cells	2021	[140]
Mineralized collagen fibrils and peptides,	Gelation and coating	*E. coli and S gordonii*	Human BMSCs	2021	[141]
Flax/silk protein-based nanofibrous scaffold	Electrospinning	*E. coli and S. aureus*	MG-63 osteoblast cells	2022	[142]
**Scaffolds with carbon nanomaterials**					
PLA-graphene and multi-walled carbon nanotubes oxides)	Solvent casting and plasma treatment	*E. coli and S aureus*	L-929 mouse fibroblast cells	2016	[143]
Polyetheretherketone and GO https://doi.org/10.1002/term.3168	Dip coating	*E. coli*	MG63 human osteosarcoma cells	2018	[144]
Percolated composites of PCL with rGO and electrostimulation	3D printing	*E. coli and S. aureus*	Human BMSCs	2020	[145]
PCL-3Dprinted fibrous scaffold and GO	Layer-by-layer	*S. epidermidis and E. coli*	HFF-1 human fibroblast cells	2020	[146]
rGO/gelatin/chitosan/TCP	3D printing	*E. coli and S aureus*	hOB human osteoblast cells	2021	[147]
Arabubinoxylan/GO/HAp/PVA hydrogel	Freeze-drying	*P. aeruginosa, E. coli and S. aureus*	MC3T3-E1 preosteoblast cells	2021	[148]
GO/HAp/bacterial cellulose and β-glucan	Radical polymerization and freeze-drying	*P. aeruginosa, E. coli and S. aureus*	MC3T3-E1 preosteoblast cells	2021	[149]
GO encapsulated forsterite (Mg_2_SiO_4_) scaffolds	Space holder processes	*E. coli and S. aureus*	MG-63 osteoblast-like cells	2022	[150]
**Scaffolds with metals/ceramics/glass**					
PLGA/Ag-TCP scaffolds	Electrospinning	*E. coli*	Not studied	2008	[151]
Ag ions 3D-glass–ceramic	Sponge impregnation method	*S. aureus*	Not studied	2008	[152]
Boron containing bioactive glass	Foam replica technique and sintering	*S. aureus*	Not studied	2009	[153]
Porous nano-HAp/titanium/polyamide66 scaffolds containing different amounts of silver ions	Inversion technique	Not specified	F12 medium	2010	[154]
Silver-loaded coral HAp	Surface adsorption process and ion-exchange	*E. coli and S. aureus*	MC3T3-E1 preosteoblast cells	2010	[155]
Ag 3D-Glass-Ceramic Scaffolds	Melt quenching and ion exchange	*S. aureus*	MG-63 osteoblast-like cells	2011	[156]
(Cu)-containing mesoporous bioactive glass	Ion exchange	*E.coli*	Human BMSCs	2013	[157]
(Chitlac-nAg)	Freeze drying	*E. coli, P. aeruginosa, S. aureus and S. epidermidis*	MG63 and Saos-2 osteoblast-like cells	2013	[158]
AgNPs containing scaffolds composed of PETA and HAp	Pressurized spray canister and expelled into molds	*E. coli and S. aureus*	Adipose MCSs	2014	[159]
Macroporous Gelatin/Bioactive-Glass/Nanosilver Scaffolds	Freeze-drying and crosslinking	*E. coli and S. aureus*	Human MSCs	2014	[160]
SiO_2_–CaO–P_2_O_5_*meso*-macroporous glass scaffolds ZnO enriched	3D printing (rapid prototyping)	*S. aureus*	HOS human osteoblast-like cells osteoblasts	2014	[161]
PLGA and TCP with Mg	Unique low-temperature rapid prototyping technology	Not specified	MC3T3-E1 preosteoblast cells	2015	[162]
Bioactive glass coated with Se NPs immobilized in PLGA particles	Foam replica method	*S. aureus, S. epidermidis*	Not studied	2015	[163]
nZn-HAp scaffold	Freeze-gelation method	*S. aureus*	Not studied	2015	[164]
Silver-doped borate bioactive glass scaffold	Foam replication technique	*E. coli and S. aureus*	MC3T3-E1 preosteoblast cells	2015	[165]
Nano-HAp/PU composite with silver phosphate particles	*In situ* foaming method	*E. coli and S. aureus*	MG63 osteoblast-like cells	2016	[166]
Zinc Cross-Linked Nanocomposite Scaffolds	Crosslinking	*E. coli and B. subtilis*	MG63 osteoblast-like cells	2016	[167]
Nano-HAp/polyamide 66 (nHP66)-based materials with silver ions and oxidized titanium	Thermal spraying technique	*E. coli and S. aureus*	Rabbit	2016	[168]
Poly (octanediol citrate)/gallium-containing bioglass composite scaffolds	Porogen-leaching technique	*E. coli and S. aureus*	Bovine bone specimens	2016	[169]
PVA/Ag scaffolds	Sponge replication	*S. aureus*	SBF fluid	2016	[170]
SiO2–Na2O–Al2O3–CaO–B2O3 Glass	Foam replication method	*E. coli, S. aureus and C. krusei*	Not studied	2016	[171]
Porous titanium with nanotubular surfaces releasing silver ions	3D printing	*S. aureus*	Human MSCs	2016	[172]
AgNPs- PEEK	3D printing	*E. coli and S. aureus*	MG-63 osteoblast-like cells	2017	[173]
Ag octahedral nanoparticle containing PCL scaffolds	Cryomilling	*P. aeruginosa*	Human MSCs	2017	[174]
Silver Doped HAp scaffolds	Wet precipitation method	S*. epidermis and P. aeruginosa*	SBF, Saos-2 human osteosarcoma cells	2017	[175]
Ag-GO nanocomposites on β-TCP bioceramic	3D printing and soaking method	*E. coli*	Rabbit bone marrow stomal cells	2017	[176]
Strontium/zinc-codoped HAp porous scaffolds	Ion-exchange and a foaming method	*S. epidermis*	MSCs	2018	[177]
PCL/TiO2	Electrospinning	*S. aureus*	hFOB human osteoblast cells	2018	[178]
Poly (l-lactic acid) (PLLA)/nano-Ag composite fibers	Electrospinning	*E. coli and S. aureus*	MC3T3-E1 preosteoblast cells	2018	[179]
PEEK/PGA/TiO_2_ scaffolds	Selective laser sintering	*E. coli and S. aureus*	Human osteoblast-like cells	2018	[180]
TiO2 scaffolds	Dark catalysis	*S. epidermis*	MC3T3 preosteoblast cells	2018	[181]
PCL/HAp/ZnO scaffold	Electrospinning	*S. aureus*	HFOb 1.19 human osteoblast cells	2018	[182]
Silver-doping of bioactive glass scaffolds	Sol-gel method	*E. coli and S. aureus*	MG-63 osteoblast-like cells	2018	[183]
Polyvinyl alcohol-starch/silver HAp	Freezing thawing	*E. coli and Bacillus* sp.	L-529 fibroblast cells	2019	[184]
PCL/CPO Coating on BCP	3D printing (robocasting)	*E. coli and S. aureus*	Not studied	2019	[185]
Biomimetic triphase pTi/CS/HAp-Se composite scaffolds	Wet-chemical method	*E. coli and S. aureus*	MDA-MB-231 breast cancer cells	2019	[186]
Ag- zincosilicate zeolite scaffolds	3D printing	*E. coli and S. aureus*	MC3T3-E1 preosteoblast cells	2019	[187]
Silver ​HAp ​based scaffolds of gelatin/alginate/PVA scaffolds	Cryogelation technique	*E. coli and B. subtillus*	MC3T3-E1 preosteoblast cells	2019	[188]
Silk fibroin/AgNPs scaffolds	Solvent casting	*E. coli*	Human MSCs	2019	[189]
Antibacterial degummed silk fiber/nano HAp/PLA with AgNPs	Cast molding method	*E. coli and S. aureus*	MC3T3-E1 preosteoblast cells	2019	[190]
Silver-doped nano HAp scaffolds	Electrospinning	*E. coli and S. aureus*	MSCs	2020	[191]
Bierarchically-structured brushite/Ag3PO4-coated Mg-based scaffoldst	Template replication method	*S. aureus, E.coli and S. epidermis*	MC3T3-E1 preosteoblast cells	2020	[192]
Ag pure scaffolds	3D printing	*S. aureus*	Not studied	2020	[193]
PLGA/Cu(I)@ZIF-8	3D printing	*S. aureus*	Murine MSCs/Rat	2020	[194]
PLA and halloysite nanotubes (HNTs) loaded with zinc nanoparticles	3D printed	*S. aureus*	MC3T3-E1 preosteoblast cells	2020	[195]
Calcium phosphate	3D printing (direct extrusion) and crosslinking	*S. aureus*	L929 fibroblast cells	2020	[196]
Phosphate-free glass–ceramic scaffolds	Freeze-drying	*E. coli*	Adipose MSCs	2020	[197]
PHBV Scaffolds Incorporated with Zinc Oxide	Selective laser sintering	*E. coli*	MG-63 osteoblast-like cells	2020	[198]
Forsterite scaffolds	3D printing and polymer-derived ceramics (PDCs) strategy	*E. coli and S. aureus*	Not studied	2020	[199]
Silver-coated grafted beta-glucan/hydroxyapatite ​nanocomposite scaffolds	Freeze-drying	*DH5 alpha ​E. coli*	MC3T3-E1 cell line	2020	[200]
Clinoenstatite-metronidazole scaffolds	Space holder method and subsequent sintering	*F. nucleatum and A. actinomycetemcomitans*	MG-63 osteoblast-like cells	2021	[201]
PCL/AgNPs scaffolds	3D printing	*E. coli*	hFOB human osteoblast cells	2021	[202]
Carbonate apatite-silver phosphate	Disolution-precipitation reactions	*S. aureus*	MC3T3-E1 and Femoral defect rabbits	2022	[203]
3D-printed scaffolds based on calcium-deficient hydroxyapatite with gold nanoparticles	3D printing	*Micrococcus luteus*	MG-63 osteoblast-like cells	2022	[204]
**Scaffolds produced by combined and alternative strategies**					
Microsphere-integrated gelatin-siloxane hybrid scaffolds	Freeze drying	*E. coli*	SBF	2008	[205]
Nano-HAp/CS/konjac glucomannan scaffolds loaded with cationic liposomal vancomycin	Freeze drying	*S. aureus*	Not studied	2011	[206]
HACC- and HACC–Zein-modified mesoporous bioactive glass scaffolds	Solvent casting and calcination	*E. coli*	Human MSCs	2013	[207]
Porous Si-nano HAp scaffolds containing vancomycin and rhBMP2	Freeze- drying method	*S. aureus*	Rat osteoblast cells/Rat	2014	[208]
HAp coatings with Ag ions and BMP-2	Electrochemical deposition (ED) and electrostatic immobilization	*E. coli and S. epidermidis*	BMSCs; osteoblasts/Rabbit	2014	[209]
45S5 Bioglass®-based scaffolds reinforced with genipin cross-linked gelatin	GCG coating	*B. subtilis and E. coli*	MG-63 osteoblast-like cells	2015	[210]
Ag-loaded SrHAp/CS porous scaffold	Freeze-drying fabrication	*S. aureus*	Human BMSCs	2016	[211]
TCP/SA with silver nanoparticles	3D printing (rapid prototyping)	*S. aureus*	Osteoblast cells	2016	[212]
Titanium Ch ​+ ​Gel ​+ ​Ag and Ch ​+ ​Gel ​+ ​Vanco.	3D printing	*S. aureus*	MG-63 osteoblast-like cells	2017	[213]
Nanostructured bredigite–amoxicillin scaffolds	Sol–gel method	*E. coli and S. aureus*	MG-63 osteoblast cells	2018	[214]
Poly-ε-caprolactone containing CS and vancomycin scaffolds	Supercritical Foaming	*E. coli and S. aureus*	MSCs	2018	[215]
Chlorhexidine-doped-PLGA/PCL (PPC) and β-TCP-doped-PLGA/PCL	Electrospinning	*S. aureus and S. mutans*	MC3T3-E1preosteoblast cells	2018	[216]
PLA-PGA matrix and silver/GO	Self-developed selective system laser sintering (SLS) system Selective laser sintering	*E. coli*	MG-63 osteoblast-like cells	2018	[217]
Doxycycline loaded Mg–Ca–TiO2 ​composite scaffold	Compactation, sintering and heating	*S. aureus and E. coli*	MG-63 osteoblast-like cells	2018	[218]
Ultrahigh-molecular-weight polyethylene reinforced by titanium with amoxycillin impregnation	3D printing and supercritical fluid impregnation	*S. aureus, S. epidermidis and E. coli*	Not studied	2019	[219]
Monticellite-CPFX scaffold	Space holder method	*E. coli and S. aureus*	MG-63 osteoblast-like cells	2019	[220]
Magnesium–Zinc scaffold containing tetracycline	Space holder technique	*E. coli and S. aureus*	Osteoblasts	2019	[221]
Xyloglucan-co-methacrylic acid/hydroxyapatite/SiO_2_ scaffold	Freeze-drying	*E. coli, ​S. aureus ​and ​P. aeruginosa*	Pre-osteoblast (MC3T3-E1) ​cell line	2020	[222]
Biomimetic scaffold composited with berberine, Ag nanoparticles and silk fibroin	Wet chemical method	*S. aureus*	MC3T3-E1 preosteoblast cells	2020	[223]
Zn-doped HAp and rGO	Mechanochemical process	*E. coli and S. aureus*	MSCs	2021	[224]
CS, carboxymethyl cellulose and Zn and Fe ions	Co-precipitation method and reeze-drying	*E. coli, S paratyphy, L monocytogenes, S. aureus*	MG-63 osteoblast-like cells	2021	[225]
Cu ions and cetyltrimethylammonium bromide loaded into montmorillonite	Cation exchange and intercalation	*E. coli*	Not studied	2022	[226]
PLA, AgNPs and GO	SLS technique	*S. aureus*	MG-63 osteoblast-like cells	2022	[227]
Cellulose and co-dispersed nanosystem (Fe3O4/GO) by free radical polymerization	Freeze-drying	*E. coli, ​S. aureus ​and ​P. aeruginosa*	Pre-osteoblast (MC3T3-E1) cell line	2022	[228]
PCLA scaffold with nano-hydroxyapatite coating doped green tea epigallocatechin-3-gallate	3D printing and coating	*S. aureus (MRSA)*	Mouse osteoblasts (MC3T3-E1)	2022	[229]

However, the antibacterial properties of recent promising scaffolds proposed for bone tissue engineering have not been studied to date. Some examples of these scaffolds include an arabinoxylan-co-acrylic acid/HAp/TiO_2_ nanocomposite scaffold [230] and a carrageenan/acrylic-acid/graphene/hydroxyapatite hybrid nanocomposite scaffold [231], both produced by freeze-drying. Arabinoxylan (ARX) and carrageenan are natural biological ​macromolecule ​with promising applications in biomedicine [230,231]. The antimicrobial properties of freeze-dried silver coated biocompatible scaffolds containing acrylic acid/guar gum, nano-hydroxyapatite, titanium nanoparticles and graphene oxide has so far not been tested [232]. These scaffolds showed promising results against mouse pre-osteoblast (*MC3T3-E1*) cell lines and increasing the amount of TiO_2_ ​in combination with GO improved physicochemical and microstructural properties, mechanical properties (compressive strength and Young's modulus), and porous properties (pore size and porosity). Another scaffold with a nacre-mimetic architecture and consisting of SrFe_12_O_19_-doped nano-layered double hydroxide/chitosan has recently been developed for bone tissue engineering [233]. The slow release of Mg^2+^ ​and Sr^2+^ ​of these scaffolds can maintain bone homeostasis and promote the formation of new blood vessels. However, their antibacterial performance has not yet been evaluated, so that it should be noted that a complete antimicrobial evaluation of developed scaffolds is essential for tissue engineering applications.

#### 3.2.1 Antibacterial scaffolds with antibiotics

The use of scaffolds for controlled localized drug release is one of the most promising techniques in tissue engineering. The aim of this method is to act on the focus of the problem and so avoid using large concentrations of possibly toxic antimicrobials to the organism or even produce microbial resistance [234]. Vancomycin (VAN) [124,125] is one of the most commonly used antibiotic with this release technique as an antibacterial agent [127,128]. VAN-laden mesoporous bioglass/PLGA composite scaffolds have been developed for this purpose [125]. These scaffolds showed a sustained release of the antibacterial drug for more than eight weeks *in vitro* producing inhibition of *S. aureus* growth and biofilm formation. These results, along with the ability to promote osteoinduction, make these scaffolds a very promising biomaterial for bone tissue engineering. VAN-PCL scaffolds maintained their antibacterial effect for more than 4 weeks [126] and showed complete inhibition of *S. aureus* [215]. A new scaffold composed of hydroxyapatite (HAp), SA and CS loaded with vascular endothelial growth factor (VEGF) and vancomycin was recently reported [130]. Gentamicin [121] and tetracycline hydrochloride (TCH) [235] are two other antibiotics used as antibacterial agents in PCL scaffolds, which revealed a significant antibacterial effect, although their toxicity in cells or animal models has not been assessed.

Some studies have developed an antibacterial bone graft by immobilizing levofloxacin hydrochloride-loaded mesoporous silica microspheres on the surface of a nano-HAp/polyurethane (PU) bioactive composite scaffold. The results show considerable antibacterial activity against both Gram-positive (*S. aureus*) and Gram-negative (*E. coli*) bacteria with a drug release for up to 42 days [127]. This approach could be a very promising strategy against chronic osteomyelitis, whose mainstay treatment is the aggressive excision of necrotic bone and infected soft tissue and prolonged local antibiotic delivery [236]. 3D scaffolds for bone regeneration based on agarose, nanocrystalline apatite, VEGF, and the antibiotic cephalexin were also capable of inhibiting the growth of *S. aureus* bacteria [128].

Krishnan et al. developed porous gelatin-hydroxyapatite (G-HAp) scaffolds loaded with various amounts of ciprofloxacin (CPFX). They observed a reduction in the growth of *S. aureus* and concluded that it has the potential to be used as a local drug delivery system. This scaffold can release effective antibiotics for reducing *S. aureus* for 60 days, with no detrimental effects on human adipose-derived mesenchymal stem cell (ADMSCs) viability or osteogenic potential [122]. 3D printed PLA/collagen/nano HAp loaded with minocycline showed increased osteogenic activity and reduced *S. aureus* biofilm formation [37]. Alkaloids from Semen Strychine, which possess antibacterial, anti-inflammation and analgesic effects, were incorporated into polyetheretherketone/polyglycolide acid (PEEK/PG) scaffolds to provide a sustained release of the antimicrobial compound against *E. coli* and *S. aureus*, as well as biocompatibility [129].

A multidrug sequential release of antibiotic agents from a hierarchical 3D scaffold was reported by García-Alvarez et al. [123] and scaffolds based on nanocomposite bioceramic and PVA with three antibiotics were produced by rapid prototyping. These three antibiotics (rifampin, levofloxacin and vancomycin) were located in different compartments of the scaffold to obtain different release kinetics. The scaffolds showed good bioactivity in preosteoblasts and were able to inhibit bacteria growth and destroy Gram-positive and Gram-negative bacteria biofilms.

Laponite nanoplates/amoxicillin-functionalized PLA nanofibrous scaffolds with osteoinductive and antibacterial activity have recently been developed by electrospinning [131].

#### 3.2.2 Scaffolds with antibacterial polymers/peptides

The intrinsic antibacterial properties of chitin, CS, cellulose and several polysaccharides of microbial origin are well known [[237], [238], [239]]. Hu et al. reported a vanillin-bioglass crosslinked 3D CS scaffold with good biocompatibility, strong antibacterial activity and capable of promoting osteoblastic differentiation prepared using a novel crosslinking technique with vanillin [139]. In other bone regeneration studies, biocomposite scaffolds containing CS were synthesized to obtain bioactive and antibacterial scaffolds [133,136]. Scaffolds based on nano-HA, starch, CS, alginate and S-nitroso-*N*-acetyl-penicillamine were manufactured by freeze-drying, obtaining porous scaffolds and an interconnected structure favorable to cell attachment and the growth of new tissue. Zhou et al. prepared a scaffold from a CS derivative (with an acrylamidomethyl group) with good prolonged antibacterial ability against *S. aureus* and *E. coli* [133].

Tissue regeneration, osseointegration, and bacterial accumulation in biomedical implants can be improved by surface modification [240]. For example, the surface modification of 3D printed PCL/HAp scaffolds has been performed with an antimicrobic polypeptide [138], providing favorable biocompatibility, osteoconductivity and antibacterial activity.

Electrostatic deposition of cationic oligopeptides in a PCL/CS nanofiber scaffold inhibited *S. aureus* while promoting osteoblast adhesion, spread, and proliferation [132]. Another strategy consists of either incorporating antibacterial peptides into the scaffolds or coating the scaffolds with them [137,140,141]. A mineralized collagen scaffold containing PLGA microspheres loaded with two antibacterial synthetic peptides was found to promote osteogenic capacity and antibacterial properties [137]. 3D printed scaffolds based on PLA/gelatin/nano HAp and the peptide ponericin showed that *E. coli* and *S. aureus* were inhibited for up to 24 ​h, and the inhibition could remain for up to 72 ​h [135]. Karamat-Ullah et al. developed a 3D hybrid aerogel-based scaffold combining an antibacterial peptide-modified silk fibroin (SF) with silica using micro-extrusion-based printing and directional freeze-casting/drying. This hybrid scaffold was found to be bactericidal against both Gram-positive and Gram-negative bacteria, and to be biocompatible with mouse embryonic pre-osteoblast (MC3T3-E1) cells [140]. 3D printed technology has also produced hydroxypropyl trimethylammonium chloride chitosan (HACC) grafted PLGA/HAp scaffolds that showed antibacterial activity against *S. aureus* and bone regeneration in infected bone defect models [134].

A novel flax/silk protein-based nanofibrous scaffold has recently been developed for bone regeneration [142]. This scaffold showed biocompatibility in MG-63 osteoblast cells and long-term antibacterial activity against *E. coli* and *S. aureus*. Flax holds bioactive peptides, which could promote antioxidant activity, antibacterial performance and anti-inflammation capacity [142].

Smart electroactive polymers have been developed to produce changes in electric charge distribution. These biomaterials, particularly conductive polymers, can deliver electrical signals by controlling the electric field applied to promote cell proliferation and differentiation, stimulating the regeneration of muscles, organs, and bones [[241], [242], [243]]. Electrostimulation applied to material surfaces appears to have an effective antibacterial activity against biofilm formation [244]. Electroactive polymers are promising materials for exploration in microbiology to develop novel strategies for fighting antibacterial resistance [241]. These materials can be useful as scaffolds for tissue regeneration to prevent infections associated with biofilm formation in implants, such as osteomyelitis in bone regeneration.

#### 3.2.3 Scaffolds with carbon nanomaterials

Carbon nanomaterials (CBNs) are one-of-a-kind carbon-based materials with unique physical and biological properties such as antibacterial activity [245] and the ability to express many genes involved in tissue regeneration [246,247]. A small amount of CBNs can improve the physical and biological properties of polymers, including mechanical performance, wettability, thermal and electrical behavior, water diffusion, cell adhesion and proliferation, antimicrobial activity and degradation [7,25,61,62,[248], [249], [250], [251], [252], [253], [254], [255]].

**Fig. 7:**
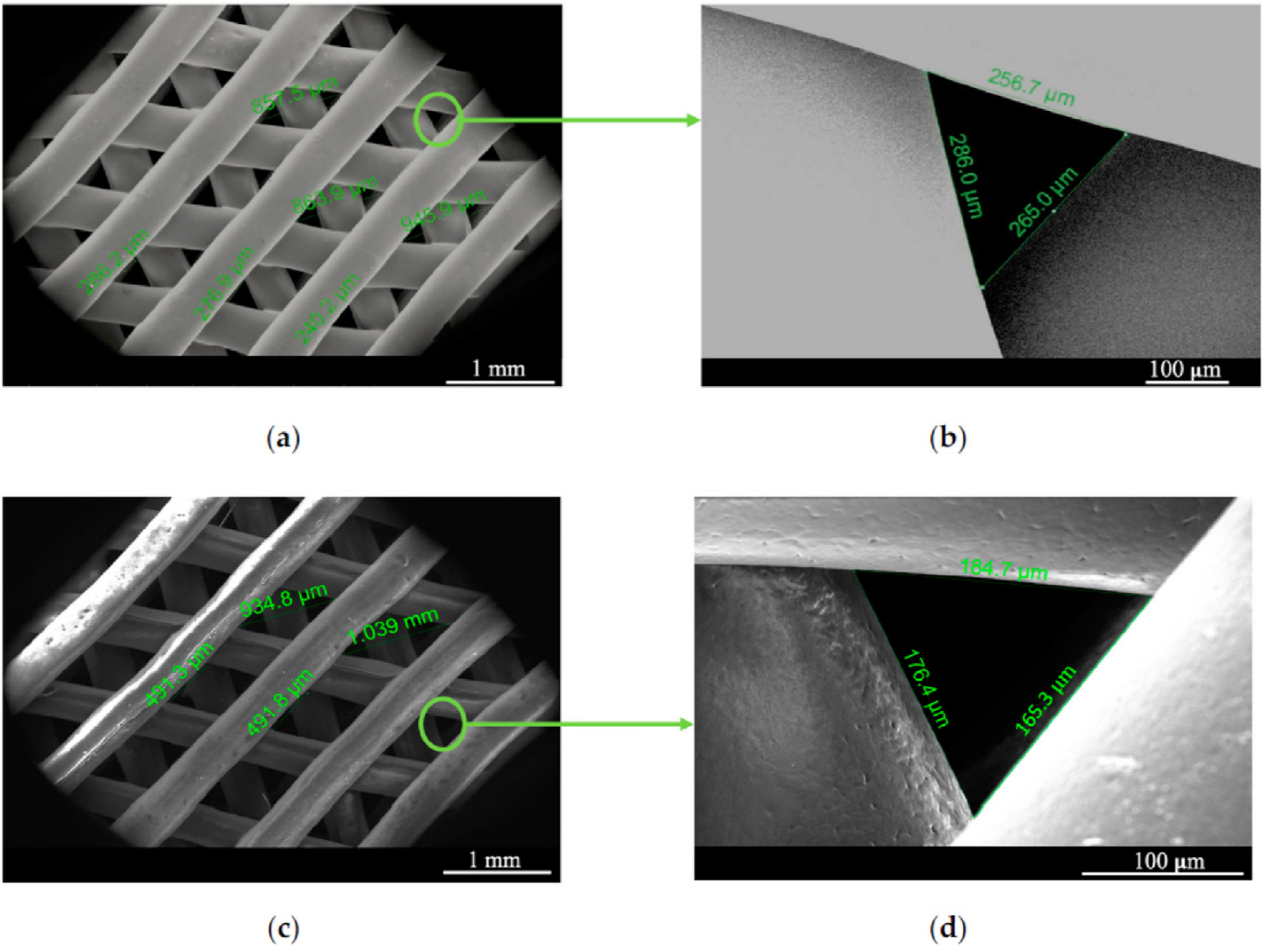
(**a**) PCL scaffold (top view); (**b**) details and pore size; (**c**) scaffold with conductive TrGO particles (top view); (**d**) detailed image of a scaffold pore. Reprinted with permission under a Creative Commons CC BY 4.0 License from Ref. [145]. Copyright 2020 MDPI.

Composites containing carbon nanomaterials with antibacterial and osteogenic activity have recently been reported [256]. Some researchers have developed 3D printed scaffolds with electroactive properties that are composed of percolated PCL composites with thermally reduced graphene oxide (TrGO), whose antibacterial activity has been tested for use in tissue engineering applications [145] (Fig. 7).

The results showed that applying an electrical stimulus of 30 ​V for 3 ​h to the surface of the 3D-printed electroactive scaffolds containing GO completely eradicated bacterial growth (*S. aureus*) on the scaffold surface. However pure scaffolds without GO possessed bacterial attachment after electrostimulation [145]. In addition to the greater bactericidal effect, the presence of highly conductive rGO linked to electrostimulation seems to increase cell viability. Recently, Lu et al. reported 3D printed scaffolds based on rGO, gelatin, CS and tricalcium phosphate (TCP) with good antibacterial activity (against *S. aureus* and *E. coli*) and without adverse effects on osteoblast viability and proliferation [147].

GO, which has low electrical conductivity, has also demonstrated antimicrobial properties. Melo et al. prepared a layer-by-layer PCL-3D printed fibrous scaffold with GO at different concentrations. The results showed good antibacterial behavior against Gram-positive and Gram-negative bacteria, and the ability to promote cell adhesion [146]. In another study, antibacterial capacity and *in vitro* osteogenesis were demonstrated by a GO-decorated microporous scaffold prepared with polyetheretherketone (PEEK), a semi-crystalline polymer used for orthopedic and spinal implants [144]. Khan et al. fabricated a composite hydrogel based on the polysaccharide ARX, GO nanosheets, HAp and PVA with bonding interactions between the components. The seeded preosteoblasts showed significant proliferation with no significant toxicity, while antibacterial activity against selected Gram-positive and Gram-negative bacteria was confirmed [148]. In a second study, the authors used GO and HAp to prepare nanocomposite scaffolds with bacterial cellulose and β-glucan. It was found that increasing the amount of GO has a positive impact on antibacterial activity and cell behavior [149].

A combination of different carbon nanomaterials has also been reported. For example, polymeric matrices of PLA and high concentrations of GO/carbon nanotubes (50/50% *w/w* of filler) were prepared by solvent casting and treated with oxygen plasma to enhance wettability [143]. The scaffolds had significant cell adhesion, showed no cytotoxicity and reduced bacterial proliferation. Several approaches using carbon nanomaterials in combination with silver nanoparticles have also given good results both as antibacterial and osteogenic biomaterials [176,217]. It should be noted that particle size is the most important characteristic that affects the antimicrobial behavior of carbon nanomaterials. The high surface-to-volume ratio of the nanoparticles (NPs) can adhere to microbial cells and affects cell membrane integrity, structural components and metabolic processes [257], which makes these new materials very promising in the field of tissue engineering, both as bioactive and biocide agents.

1% GO encapsulated forsterite (Mg_2_SiO_4_) scaffolds recently showed a porosity of 76%–78% with pore size of 300–450 ​μm, good cell biocompatibility, enhanced cell proliferation and potent antibacterial performance for bone tissue engineering [150].

#### 3.2.4 Scaffolds with metals/ceramics/glass

Several studies in the field of antimicrobial scaffolds are based on silver (Ag), zinc (Zn), magnesium (Mg) or strontium (Sr) ions, due to their known antibacterial properties. Nanocomposite antibacterial scaffolds were prepared by loading AgNPs with an adsorption process. Overall, the results show that AgNPs confer good antibacterial properties on composite scaffolds to impede early infections [158]. Scaffolds composed of TiO_2_ nanotubes manufactured by 3D printing and silver ions were subsequently incorporated into their surface. The antimicrobial effect against *S. aureus* was found to continue for two weeks [172].

Some researchers have developed AgNPs containing antibacterial scaffolds composed of pentaerythritol triacrylate-co-trimethylolpropane tris (3-mercaptopropionate) (PETA) and HAp. These scaffolds showed osteoinductive and degradable properties capable of stimulating the proliferation of bone progenitor cells, did not affect cell viability and inhibited the proliferation of *S. aureus* and *E. coli* [159]. Gelatin/bioactive glass/AgNP scaffolds showed good cytocompatibility to human mesenchymal stem cells (hMSCs) and antibacterial capacity against *E. coli* and *S. aureus* [160]. Researchers investigated the antimicrobial activity of Ag octahedral nanoparticles containing PCL scaffolds, which showed antibacterial activity, osteogenic differentiation and no adverse effects on hFOB and hMSCs cells [174,202].

Polymeric PLA scaffolds with metals such as silver, which give a final result of antibacterial activity, showed osteogenic differentiation and no cytotoxicity to human cells [179]. A continuous Ag^+^ release can last more than 3 weeks, which can be useful in long-term bone implants. Zhang et al. synthesized a brushite/Ag_3_PO_4_-coated Mg-Nd-Zn-Zr scaffold to substitute bone [192]. This new scaffold demonstrated high antibacterial activity against Gram-positive and Gram-negative bacteria, with appropriate degradation characteristics and cytocompatibility. Arjunan et al. manufactured a pure Ag scaffold and demonstrated its antibacterial efficacy against *S. aureus* [193]. SF films with AgNPs have also been developed, since silk fibroin is a suitable biomaterial for bone tissue engineering. SF/AgNPs scaffolds showed cytocompatibility and an effective antibacterial effect against Gram-negative and antibiotic-resistant bacteria [189]. Silver-coated bioactive nanocomposite scaffolds have been developed using a polymeric matrix of beta-glucan biopolymer, acrylic acid, and nano-hydroxyapatite through free radical polymerization and freeze drying [200]. These scaffolds showed an antibacterial effect against DH5 alpha *E. coli* with no cytotoxicity in MC3T3-E1 cells.

Some researchers have combined silver with other materials to prepare antibacterial scaffolds. For example, silver-doped HAp scaffolds (HAp/Ag) reduced *E. coli, S. aureus* and *S. epidermis* bacterial populations while maintaining cytocompatibility with mammalian cells [175,191,258]. PVA-starch/HAp/Ag scaffolds prepared by freezing-thawing also demonstrated antibacterial activity against Gram-positive *Bacillus* and Gram-negative *E. coli* [184]. In another study, Deng et al. developed Ag-decorated 3D printed PEEK scaffolds *via* catecholamine chemistry. The antibacterial tests performed indicated that these Ag-PEEK scaffolds showed significant antibacterial effects against Gram-negative and Gram-positive bacteria and could support the proliferation of MG-63 osteoblast cells [173]. In another study, Wang et at [187]. proposed Ag-incorporated zincosilicate zeolite scaffolds with compressive strength and a Young's modulus similar to human cancellous bone. The scaffolds showed good antibacterial and bioactivity, indicating their potential as antimicrobial materials for bone substitutes. Antibacterial degummed silk fibers (ADSF) in combination with nano-HAp and PLA have been prepared, including Ag-nanoparticles as a reinforcing material [190]. The biological and antibacterial assessments showed that the ADSF/nano HAp/PLA composites had good bioactivity and antibacterial properties.

Bioactive glass-ceramic scaffolds [152,156] or coral hydroxyapatites [155] combined with Ag ions have been reported as a good approach for preparing scaffolds with antibacterial properties. In a similar approach, gelatin composite scaffolds were made by gelatin, alginate, PVA, nano-silver and HAp. These reinforced scaffolds showed antibacterial activity against Gram-positive and Gram-negative bacteria, as well as good biocompatibility in MC3T3-E1 preosteoblast cells [188].

Scaffolds with TiO_2_ have shown strong antibacterial activity [180,181]. PCL/TiO_2_ nanocomposite coatings were developed with a good bioactive performance against osteoblast cell lines and excellent antimicrobial behavior against *S. aureus* [178]. TCP/silver/PLGA scaffolds (TCP/Ag/PLGA) with a proven prolonged antibacterial effect against *E. coli* [151] have also been reported. Some researchers developed HAp scaffolds with Ag/TiO_2_/PA66 [154], Sr, Zn [164,177], or Ti6Al4V (pTi), CS and selenium (Se) (pTi/CS/HAp-Se) [186]. They observed osteoblast proliferation, tumor cell growth inhibition and bacterial viability with pTi/CS/HAp-Se scaffolds. Jiang et al. developed nano HAp/PU scaffolds with varying concentrations of Ag_3_PO_4_ particles for the repair of infectious bone defects [166]. The incorporation of Ag_3_PO_4_ in nano HAp/PU scaffolds increased their antibacterial potential against both Gram-positive and Gram-negative bacteria. The antibacterial tests and cytocompatibility evaluation revealed that nano HAp/PU scaffolds with 3% *w/w* Ag_3_PO_4_ had stronger antimicrobial effects and satisfactory cytocompatibility.

A novel porous nano HAp/polyamide 66 (nHP66)-based nanoscaffold material containing varying concentrations of silver ions (Ag^+^) (TA-nHP66) and oxidized titanium (TiO_2_) was developed successfully in an experimental osteomyelitis study in rabbits [168]. Porous osteoinductive TA2-nHP66 scaffolds with a composition of 0.64% *w/w* of Ag^+^ and 2.35% *w/w* of TiO_2_, were shown to have strong antibacterial activity against *S. aureus* and *E. coli in vitro* and *S. aureus in vivo*.

A new biomaterial composed of PLA, halloysite nanotubes loaded with ZnO nanoparticles was prepared by 3D printing [195]. The scaffolds so prepared showed osteoinductive potential. The external coating with gentamicin preserved the osteogenic properties and reduced bacterial growth. Zhu et al. prepared a forsterite scaffold by combining 3D printing and polymer-derived ceramics that contain biometal Mg [199]. The scaffolds showed efficient photothermal-induced antibacterial activity.

Bioactive glass scaffolds have many advantages such as osteoconductivity and osteoinductivity, making them an ideal scaffold for bone tissue engineering applications [259]. The development of multifunctional bioactive scaffolds that combine angiogenesis activity, a capacity, and antibacterial performance for regenerating lost bone tissues is of great importance in this field [260]. The antimicrobial activity of selenium nanoparticles (SeNPs) has also been reported [261,262]. Adding Ag^+^ to bioactive glasses has been investigated to produce antibacterial glasses [263]. Bioactive glass scaffolds have also been developed with delivery systems. Poly (octanediol citrate) bioactive glass scaffold containing zinc and gallium ions demonstrated antibacterial activity against Gram-positive and Gram-negative bacteria as well as cytocompatibility with human cells [169]. Silver-doped bioactive glass scaffolds showed antibacterial activity against *S. aureus* and *E. coli.* The scaffolds mimicked cancellous bone in terms of architecture and mechanical properties [170,183]. Some researchers obtained bioactive glass scaffolds from a soda-lime glass powder consisting of microspheres belonging to the SiO_2_–Na_2_O–Al_2_O_3_–CaO–B_2_O_3_ system [171] and compared this new scaffold with the 45S5 Bioglass® scaffold and found its antibacterial activity to be higher against *C. krusei*. Other researchers modified the 45S5 Bioglass® scaffold to improve its characteristics. For example, Gorriti et al. added free boron to 45S5 Bioglass® scaffold and the bactericidal effect increased by 55% [153]. A new 45S5Bioglass®/PLGA/SeNPs scaffold was fabricated to combine the antimicrobial properties of SeNPs with the osteoinductive capacity of bioactive glass to achieve bone regeneration [163].

Scaffolds made of borosilicate bioactive glass doped with varying amounts of Ag_2_O showed a sustained release of Ag ​^+^ ​over more than 8 weeks and resistance against colonization by the bacterial strains *E. coli* and *S. aureus* [165]*.* Phosphate-free glass-ceramic porous scaffold is another example of antibacterial bioglass scaffolds. This can be synthesized by a three-step method involving slurry preparation, induction of porosity by surfactant-assisted foaming, followed by freeze-drying and sintering [197]. Hayashi et at. fabricated antibacterial honeycomb scaffolds by a procedure consisting of the replacement of their principal component (carbonate apatite) for silver phosphate on their surface [203]. Scaffolds containing 9.9·10^−4^% *w/w* silver phosphate showed antibacterial activity against *S. aureus* and allowed MC3T3-E1 preosteoblast proliferation and differentiation. They also prevented bacteria from growing in a rabbit with a femoral defect, which had *S. aureus* in it and new bone started to grow two weeks after surgery.

**Fig. 8:**
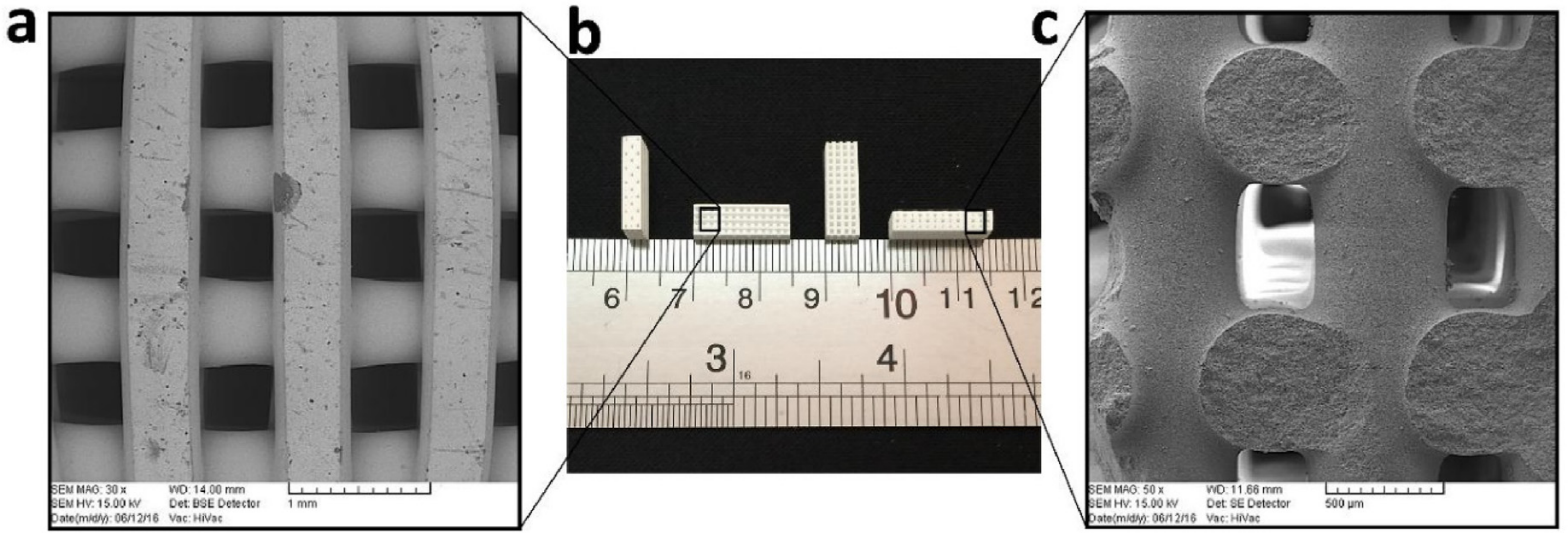
View of sintered robocast biphasic calcium phosphate scaffolds for bone tissue engineering. Optical (**b**) and scanning electron microscope view from the top (**a**) and cross-section **(c**) of the ceramic scaffold. Reprinted with permission from Ref. [185]. Copyright 2019 Elsevier.

Hypoxia is one of the key factors that can affect scaffold implantation and lead to cell necrosis and microbial infection [264]. To solve this problem, oxygen-releasing bioceramic scaffolds were fabricated from biphasic calcium phosphate (BCP) powder [185] (Fig. 8).

**Fig. 9:**
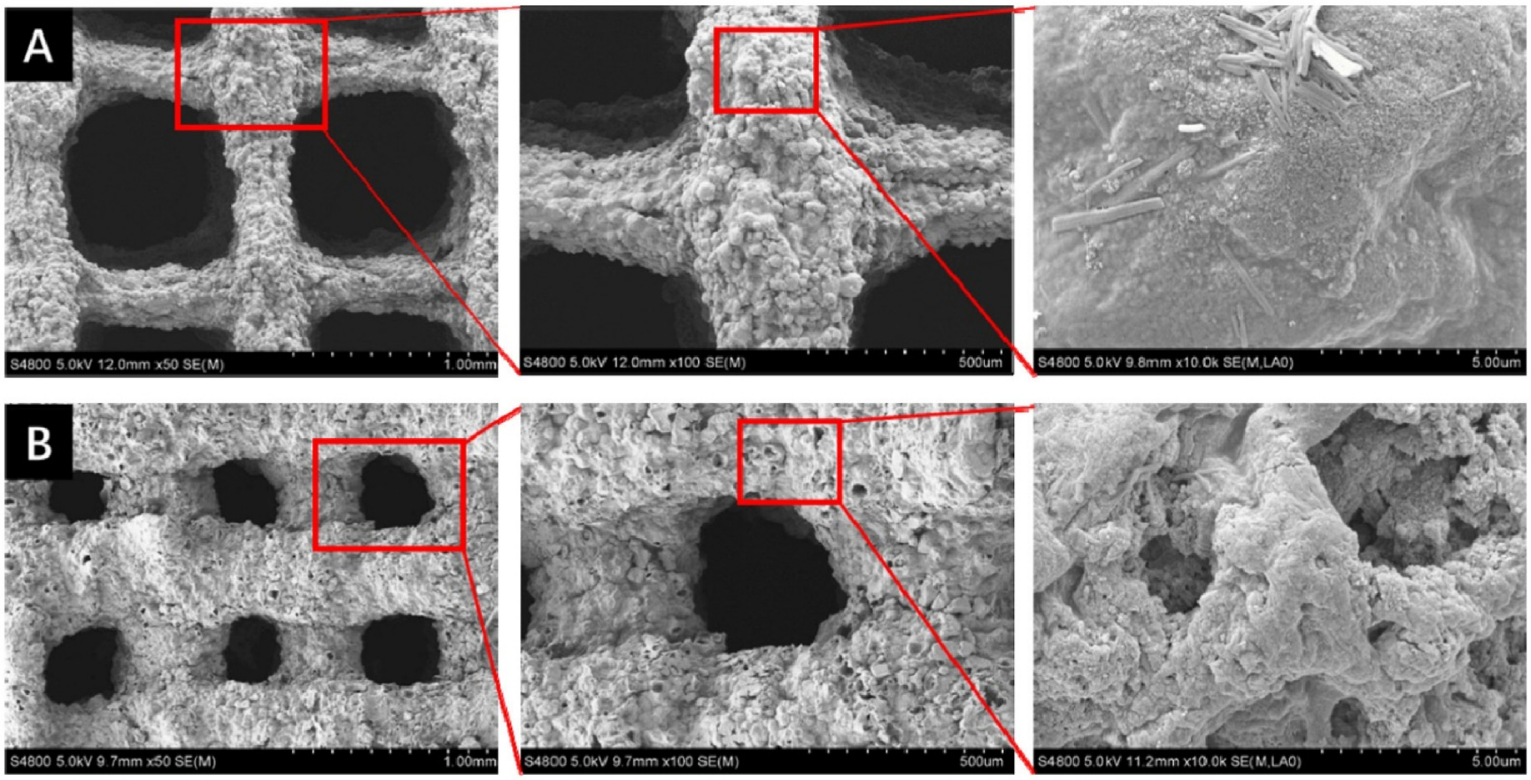
(**A**) Uncrosslinked scaffold after freeze-drying, the printed struts shrunk sharply and showed one-level macroporous structures. (**B**) Crosslinked scaffold after freeze drying showed multi-level porous structures. Reprinted with permission from Ref. [196]. Copyright 2020 Elsevier.

Calcium phosphate scaffolds with specific designs in terms of pore size, shape, and porosity can be precisely produced by AM technology [196]. *In situ*, the porous 3D printed bioceramic material was crosslinked with SA and freeze-dried. Scanning electron microscope (SEM) images revealed that the crosslinked scaffold has a multi-level porous structure compared to the uncross-linked one (Fig. 9).

At the same time the scaffolds were loaded with berberine, a quaternary ammonium compound with antibacterial activity and showed both antibacterial and bone-promoting functions. *In vitro* studies indicated that the 3DP scaffolds had low cytotoxicity with a beneficial effect on MC3T3 cell adhesion and proliferation [196].

Copper-containing mesoporous bioactive glass (Cu-MBG) scaffolds stimulate the hypoxia-inducible factor (HIF)-1a and VEGF expression in human bone marrow-derived mesenchymal stromal cells (BMSCs). Antibacterial Cu-MBG scaffolds promoted the osteogenic differentiation of human BMSCs and maintained a sustained release of ibuprofen [157]. Magnesium (Mg) has also been reported to possess antibacterial activity [265]. For example, a PLGA/Mg scaffold fabricated by a low-temperature rapid-prototyping technique showed an ability to inhibit bacterial growth and biofilm formation [162].

Zinc is an essential element with intrinsic antibacterial and osteoinductive capacity [266]. Zinc cross-linked scaffolds significantly reduced the growth of *Bacillus subtilis* and *E. coli* by 70 and 81%, respectively [167]. PCL-ZnO nanofibrous scaffolds have been developed with antibacterial activity against *S. aureus* and are also capable of inducing early mineralization with ZnO concentration-dependent degradation [182]. ZnO-enriched *meso*-macroporous glass scaffolds were prepared by Sánchez-Salcedo et al. The results showed that the porous structure was suitable for osteoblast growth and that the Zn ions released exhibited antibacterial properties against *S. aureus* [161]. ZnO nanoparticles have also been incorporated into PHBV to produce antibacterial porous scaffolds [198].

**Fig. 10:**
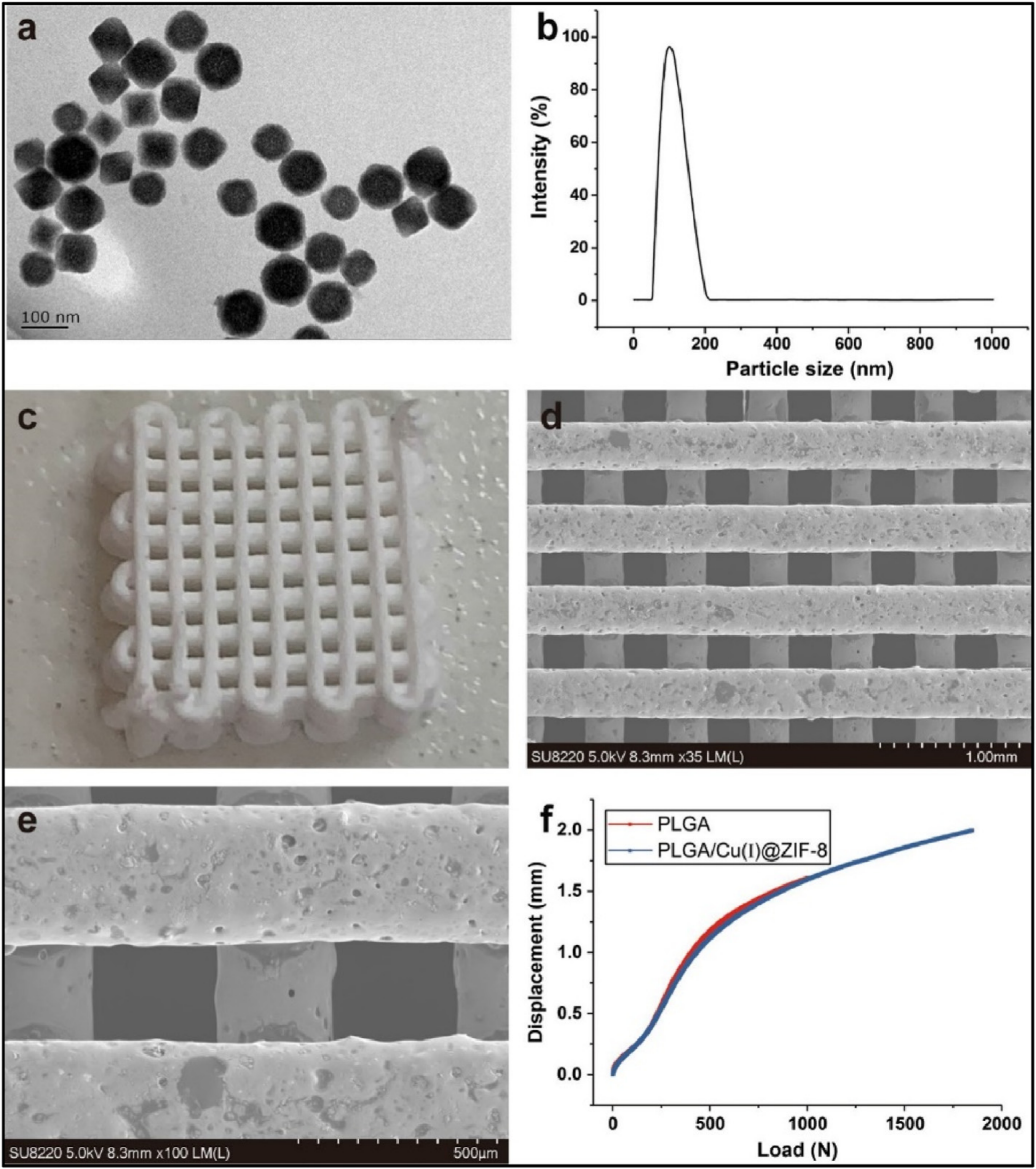
Scaffolds composed of copper-loaded-zeolitic-imidazolate-frameworks (ZIF-8) and PLGA (PLGA/Cu(I)@ZIF-8): (a) Transmission electron microscope (TEM) image of Cu(I)@ZIF-8 nanoparticles; (b) Particle size distribution of Cu(I)@ZIF-8 nanoparticles; (c) digital image; (d, e) TEM images of PLGA/Cu(I)@ZIF-8 scaffolds; (f) Load–displacement curve of PLGA and PLGA/Cu(I)@ZIF-8 scaffolds. Reprinted with permission under a Creative Commons CC BY 4.0 License from Ref. [194]. Copyright 2020 Springer Nature.

PLGA is one of the most commonly used polymer biomaterials for producing bone tissue engineering scaffolds, since this biodegradable copolymer does not have any side effects when used as a medical material [267,268]. A novel PLGA/Cu(I)@ZIF-8 scaffold for infected bone repair was created by combining antibacterial copper-loaded-zeolitic-imidazolate-frameworks (ZIF-8) and PLGA [194], as shown in Fig. 10.

3D-printed biocompatible scaffolds based on calcium-deficient hydroxyapatite (CDHA) with gold nanoparticles showed effective antibacterial activity against *Micrococcus luteus* for bone tissue engineering [204].

Bio-ceramic clinoenstatite (MgSiO_3_) scaffolds of different micropore sizes were fabricated by the space holder method and subsequent sintering [201]. They showed good mechanical strength as well as biocompatibility in MG63 ​cells and controlled drug-release potential of metronidazole (MET) towards the *Fusobacterium nucleatum* and *Aggregatibacter actinomycetemcomitans* bacteria.

#### 3.2.5 Antibacterial scaffolds produced by combined and alternative strategies

Biological and antibacterial properties of titanium implants are required to prevent implant-associated infections and promote cell attachment of orthopedic devices. Several antimicrobial scaffold delivery systems have been developed in this research line as an emerging technology for the reconstruction of bone and cartilage tissue defects [269]. A reinforced hybrid scaffold consisting of continuous and porous layers of titanium and ultrahigh-molecular-weight polyethylene (UHMWPE), a polymer with good compatibility, and a subsequent amoxycillin impregnation were prepared to prevent the appearance of opportunistic infections [219]. As a result, the contact of Gram-positive and Gram-negative bacterial cultures (*S. aureus*, *S. epidermidis* and *E. coli)* with the hybrid polymeric scaffolds suppressed microorganism growth and colony formation. A new bioactive monticellite-ciprofloxacin (Mon-CPFX) scaffold was created by the same researchers using the space holder method [220]. This scaffold showed good cell attachment and growth, suitable compression performance and drug release and an excellent antibacterial capacity [220]. In another study, Thanyaphoo reported on Si-nano HAp scaffolds loaded with vancomycin or recombinant human bone morphogenetic proteins (rhBMP-2) which showed potential to be used as a drug delivery system to kill *S. aureus* [208]*.*

Ceramic porous scaffolds loaded with antibiotics have also been proposed as an alternative approach. Bakhsheshi-Rad et al. prepared biocompatible bredigite–amoxicillin scaffolds with good antibacterial activity against both *S. aureus* and *E. coli* bacteria [214]. Doxycycline in a low concentration in a Mg–Ca–TiO_2_ composite scaffold showed no cytotoxic behavior against MG63 ​cells but did have efficient antibacterial activity against Gram-positive and Gram-negative pathogens [218].

Layer-by-layer electrospinning was used to construct chlorhexidine-doped PLGA/PCL (PPC), PLGA/PCL (PP), and β-tricalcium phosphate-doped-PLGA/PCL (PPβ) [216]. The three-layer electrospun membranes showed high strength, good cell adhesion, promoted osteoconductive properties and enhanced antimicrobial properties. Other researchers have engineered scaffolds that combine several strategies. For example, Xie et al. studied the antibacterial activity of AgNPs combined with Hap, CS, and bone morphogenic protein-2 (BMP-2) [209]. Ti bars with BMP/CS/Ag/HAp coatings were implanted into rabbit femurs [209]. In another study, an Ag-loaded strontium hydroxyapatite (SrHAp)/CS scaffold (Ag-SrHAp/CS) was prepared to analyze its biocompatibility, osteoinductivity, and antibacterial activity [211]. The Ag ions released from the scaffold inhibited the growth and attachment of *S. aureus.* In another study, Mg and Zn ions were combined with the antibiotic tetracycline to prepare a scaffold by the space-holder technique [221]. The results indicated that the engineered Mg–Zn scaffolds containing 1–5% of tetracycline had good potential for bone tissue healing due to their good biocompatibility and antibacterial activity.

The biodegradable polyester PLA, approved by the Food and Drug Administration (FDA) for direct contact with biological fluids, is a promising biodegradable polymer for the fabrication of biocompatible scaffolds [270]. However, it is not antibacterial and needs to be combined with antibacterial agents to provide protection against infections. These combinations can be very simple; for example, mixing, polymeric PLA scaffolds with metals such as silver or carbon nanomaterials, which provide antibacterial activity, osteogenic differentiation and no cytotoxicity to human cells [179,217]. PLA is a relatively hydrophobic polymer that can be combined with hydrophilic polymers such as collagen (COL), minocycline hydrochloride (MH) and citrate-hydroxyapatite nanoparticles (cHApNPs) to reduce bacterial adhesion and biofilm formation [37]. The presence of minocycline hydrochloride also enhances the biological properties of the composite material. These scaffolds can inhibit *S. aureus,* which is a major pathogen in bone-associated infections because of its ability to adhere and form biofilms on bone and/or implants [271].

The gelatin-siloxane hybrid scaffold with gentamicin sulfate is another example of a scaffold with excellent bioactivity and antibacterial capacity [205]. Li et al. coated the 45S5 Bioglass® scaffold with genipin cross-linked gelatin (GCG) and further incorporated it with poly (*p*-xylyleneguanidine) hydrochloride (PPXG) to produce a biocompatible scaffold with antibacterial activity against Gram-positive and Gram-negative bacteria [210].

The combination of antimicrobial polymers (particularly CS) and antibiotics is another strategy to treat bone biofilm infection or provide antibacterial activity. Nano HAp/CS/Konjac glucomannan scaffolds [206] and PCL/CS [215] loaded with vancomycin have demonstrated antimicrobial activity. Another strategy is the combination of multiple antibacterial agents to prevent the use of toxic levels. The synergistic effects of silver ions and the antibiotic vancomycin, together with the antimicrobial CS, were assessed in a scaffold prepared from CS/gelatin/Ag and loaded with vancomycin [213]. A CS/carboxymethyl cellulose with Zn and Fe integrated hydroxyapatite (ZFHAp) scaffold has also been proposed [225]. The combination of the antibacterial properties of CS and the release of Zn ions (5% of ZFHAp) resulted in a robust antibacterial activity and good biocompatibility with bone cells.

Some researchers have developed different mesoporous bioactive glass (MBG) scaffolds [207], which are very brittle and lack antibacterial activity. To avoid these disadvantages, a novel modified MBG scaffold was developed with prolonged antibacterial activity and demonstrated biocompatibility with hMSCs [207]. Scaffolds produced by other alternative strategies, such as baghdadite-vancomycin scaffolds reloaded with a drug for 6 ​h presented antibacterial activity against *S. aureus* [124]*.* Hu et al. demonstrated that berberine/Ag nanoparticle embedded biomimetic calcium phosphate scaffolds showed enhanced antibacterial performance [223].

Xyloglucan-co-Methacrylic Acid/Hydroxyapatite/SiO_2_ nanocomposite scaffolds showed important properties for bone tissue engineering such as potent antimicrobial activity against several Gram-positive and Gram-negative strains, porosity with substantial mechanical strength, biodegradability, biocompatibility and cytocompatible behavior [222].

Recently, Yu et al. developed a novel antibacterial PGA-based scaffold produced by cation exchange of montmorillonite (MMT) with Cu^+2^ and the intercalation of cetyltrimethylammonium bromide (CTAB) into the interlayer of MMT [226] that showed superior antibacterial activity.

An antibacterial metal in combination with carbon nanomaterials has been proposed as a new approach. Ag-GO nanohybrids, prepared by AgNPs *in situ* grown on GO, were introduced into PLA to produce biocompatible and antibacterial scaffolds using the SLS technique [227].

In another study, Zn-doped HAp/rGO nanocomposites were prepared using a mechanochemical process [224]. Zn doping in combination with rGO promoted alkaline phosphatase (ALP) activity and proliferation of MSCs as well as antibacterial performance.

Polymeric nanocomposite scaffolds composed of cellulose and co-dispersed nanosystem (Fe_3_O_4_/GO) were very recently produced by free radical polymerization and freeze drying [228]. These electroactive scaffolds showed good biocompatibility in a pre-osteoblast (MC3T3-E1) cell line and potent antibacterial activity against Gram-positive *S. aureus* and Gram-negative *E. coli* and *Pseudomonas aeruginosa*. Using another approach combining different strategies, a 3D-printed PCLA scaffold with nano-hydroxyapatite coating doped green tea epigallocatechin-3-gallate promoted bone growth and inhibited multidrug-resistant bacteria colonization [229].

### 3.3 Antibacterial scaffolds for skin regeneration

Soft tissue infections in open fractures, burns or diabetic complications are some of the main causes of high morbidity [272]. Functional antibacterial skin tissue scaffolds are being developed to treat large and deep skin defects (see Table 2). Several strategies are being investigated to treat and promote wound healing, such as the release of antibiotic drugs or antibacterial biometals, the use of polymers, biopolymers or peptides with intrinsic antibacterial properties, the incorporation of nanomaterials with antibacterial properties, such as carbon nanomaterials, or the combination of different strategies. This section describes the different approaches developed in recent years.

**Table 2:** Antibacterial scaffolds for skin tissue engineering applications.

Material	Fabrication method	Bacteria	Non-toxicity: cell line/animal model	Year	Ref
**Scaffolds with antibiotics**					
PLA, PCL and CPFX	Jet spraying	*Bacillus subtilis and E. coli*	Dermal fibroblast	2017	[273]
PLGA electrospun fibers containing CPFX	Electrospinning	*P. aeruginosa, S. aureus and S. epidermis*	Not studied	2018	[274]
CPFX-modified degradable hybrid PU-PLA porous scaffolds	Polymerization	*E. coli, S. aureus and P. aeruginosa*	Not studied	2020	[275]
Alginate, methylcellulose and Laponite	3D printing	*S. aureus and S. epidermidis*	Not studied	2021	[272]
Porcine acellular dermal matrix hydrogel blended with vancomycin	Decellularization, digestion and load	*S. aureus and Enterococcus*	Mouse embryonic cells (NIH3T3 cells)/Rat	2021	[276]
Microstructural nanofibrous mats/gentamicin-loaded hydrogel scaffold	Electrospinning	*S. aureus and P. aeruginosa*	Human dermal fibroblast cells/rat	2022	[277]
**Scaffolds with metals/glass**					
PCL nanofibers containing different ratios of calcium peroxide with or without ascorbic acid	Electrospinning	*E. coli and S. epidermis*	hFOB human osteoblast cells	2011	[278]
Cellulose–polymer–Ag nanocomposite fibers	Rotating the preweighed and washed cellulose fibers	*E. coli*	Not studied	2013	[279]
PCL/gelatin nanofibrous scaffolds coated with silver	Electrospinning	*B. cereus and E.coli*	HSF human splenic fibroblast cells	2016	[280]
Silver nanoclusters/nanoparticles hemostatic material	3D printing	*E. coli, P. aeruginosa, S. aureus and MRSA*	A549, U251, HepG2, HBE/Rabbit	2018	[281]
Radio sterilized pig skin ​+ ​AgNPs	Impregnation	*S. aureus and S. maltophilia*	MSCs	2018	[282]
SiO_2_–CaO mesoporous bioactive glass NPs with silver	Microemulsion-assisted sol-gel method	*S. aureus and P. aeruginosa*	3T3 fibroblast cells	2019	[283]
S-AgNPs loaded PVA nanofiber	Electrospinning and Cross-lining	*E. coli and S. aureus*	Not studied	2019	[284]
PVA/Starch cryogel scaffold combined with AgNPs	Cryogelation technique	*P. aeruginosa*	Not studied	2019	[285]
PGS/PCL nanofibers with calcium peroxide	Electrospinning	*S. aureus*	BMSCs	2020	[286]
PCL nanofibrous mat with silver sulfadiazine	Electrospinning	*S. aureus and P. aeruginosa*	Human dermal fibroblasts/Rat	2020	[287]
Bioglass-based scaffolds coated with AgNPs	Sponge replication technique	*S. aureus, P. aeruginosa and C. albicans*	Not studied	2020	[288]
Silica-based nanocomposites hydrogel scaffolds	Crosslinking	*E. coli and S. aureus*	Endothelial progenitor cells/Mice	2020	[289]
PCL and Ag-magnetite NPs	Co-precipitation and electrospinning	*E. coli and S. aureus*	Human melanocytes/Rats	2021	[290]
Lignin-agarose hydrogel-silk fibroin and zinc chromide NPs	Crosslinking	*P. aeruginosa*	Hu02 fibroblast cells/Mice	2021	[291]
Gelatin-based and Zn^2+^-incorporated composite hydrogels	Polymerization in solution	*E. ​coli ​and ​S. ​aureus*	NIH-3T3 cells/Mice	2022	[292]
**Scaffolds with antibacterial polymers/peptides**					
Quaternary chitin/partially deacetylated chitin nanofibers	Freeze-shaping and drying	*E. coli and S. aureus*	L929 mouse fibroblast/Rat	2017	[293]
PCL/CS scaffold	3D printing	*S. aureus and S epidermis*	L929 mouse fibroblast cells	2018	[294]
CS/aminoacid hydrogels	Dissolution	*Not specified*	SBF fluid	2018	[295]
Biomimetic Composite Nanfibrous Scaffolds	Electrospinning	*S. aureus*	Human immortalized epidermal cells	2019	[296]
ECM from decellularized mammalian tissue and ECM (CS)	Decellularized	*E. coli and S. aureus*	HMEC-1 endothelial cells	2020	[297]
Micro/nanostructured poly (butylene-succinate-*co*-adipate)	Phase separation	*S. epidermidis*	HaCaT keratinocyte cells	2020	[298]
Silk fibroin and vitamin K3 carnosine peptide	Electrospinning	*S. aureus, E. coli and P. aeruginosa*	HGF1, NIH 3T3 fibroblast cells/Rat	2021	[299]
Silk fibroin/Gelatin and CM11 peptide	Freese-drying	*S. aureus, E. coli, P aeruginosa*	Hu02 fibroblast cells	2022	[300]
**Scaffolds with carbon nanomaterials**					
PHBV, collagen and rGO	Electrospinning	*E. coli and S. aureus*	3 T3-L fibroblast-like cells	2017	[301]
Isabgol and rGO	Freeze-drying	*E. coli and S. aureus*	NIH 3T3 fibroblast cells/Winstar rats	2018	[302]
PU, polyhexamethylene guanidine hydrochloride and GO	Freeze-drying	*E. coli and S. aureus*	HaCaT keratinocyte cells/Micet	2020	[303]
Cellulose, graphene quantum dots	Solvent casting	*S. aureus, E. coli, P. aeruginosa*	Human fibroblast	2022	[304]
Calcium alginate, PHBV and graphene nanoplatelets	Solvent casting	Not studied	Human keratinocyte (HaCaT) cells	2022	[305]
**Scaffolds produced by combined strategies and alternative methods**					
Quercetin-Containing PLGA Nanofibrous Scaffolds	Electrospinning	*S. aureus and K. pneumoniae*	KB epithelial cells	2012	[306]
Honey/CS nanofibrous scaffolds loaded with natural materials	Electrospinning	*E. coli, S. aureus, MRSA and P. aeruginosa*	Human fibroblast cells/Mice	2016	[307]
Porous CS-selenium scaffolds and porous CS-silver scaffolds	Deposition method	*E. coli and S. aureus*	Fibroblasts	2018	[308]
CS 2D film scaffolds and nanoparticles enriched with royal jelly and grape seed extract	Mixing	*B. subtilis, S. aureus, E. aerogenes, and P. aeruginosa*	Human lung fibroblast cells	2018	[309]
Polyhydroxyalkanoate/graphene silver nanocomposite	Electrospinning	*E. coli and S. aureus*	Not studied	2018	[310]
Bilayered silk fibroin-based scaffolds	Freeze drying	*S. aureus*	Not studied	2018	[311]
Quaternary ammonium organosilane cross-linked nanofibrous collagen scaffolds	Electrospinning	*S. aureus and S. epidermis*	hFOB osteoblast, hDF fibroblasts cells	2018	[312]
PCL/gelatin/Lawsone Nano Fiber Scaffolds	Electrospinning	*S. aureus, MRSA, P. aeruginosa and P. mirabilis*	Not studied	2018	[313]
Halloysite nanotube (HNT)-reinforced alginate-based nanofibrous scaffolds	Electrospinning	*S. aureus and S. epidermidis,* *P. aeruginosa and E. coli*	L929 mouse fibroblast cells	2018	[314]
Cryogel, Hydrogel, and electrospun scaffolds	Electrospinning	*S. aureus*	Not specified	2019	[315]
PLA and cellulose acetate with thymoquinone	Electrospinning	*E. coli and S. aureus*	3T3-L1 fibroblast-like cells/Mouse	2019	[316]
CS based collagen/gelatin composite scaffolds	Freeze drying	*E. coli and S. aureus*	Not studied	2020	[41]
PLA scaffolds with ascorbic and fumaric acids	Electrospinning	*E. coli and S. aureus*	Not studied	2020	[317]
RSF/HACC-BAMG scaffolds	Electrospinning	*E. coli and S. aureus*	Schwann cells/Rabbit	2020	[318]
CS cryogel microspheres decorated with silver nanoparticles	Emulsification method and crosslinking	*E. hirae, B. cereus,* *S. aureus, L. pneumophila, E. coli,* *P. aeruginosa and C. albicans*	Not studied	2020	[319]
Collagen/CS and CPFX	Freeze-drying	*E. coli and S. aureus*	Fibroblast	2021	[320]
Collagen/CS, calcium peroxide and CPFX	Freeze-drying	*E. coli and S. aureus*	Fibroblast/Rat	2021	[321]
Polylysine, rGO and Ag ions	Functionalization	*S. aureus*	3 T6 fibroblasts, red blood cells/Rat	2021	[322]
Graphene and ion metals	Drop casting coating method	*A. baumannii, S. aureus, K. pneumoniae and P. aeruginosa*	Not studied	2021	[323]
ARX, CMARX, TEOS ​loaded with 5FU onto rGO	Cast into glass Petri dishes & dry at 55 ​°C in an oven.	*S. aureus and P. aeruginosa*	Not study/Anticancer against U87	2021	[324]
CS/guar gum/PVA blended hydrogels with different crosslinking amounts of TEOS	Vacuum dried at 55 ​°C	*S. aureus, ​Bacillus cereus, P. aeruginosa ​and ​E. coli*	Not studied	2021	[325]
CS/PVA/GO based pH-responsive composite hydrogels crosslinked with TEOS	Solution casting method	*E. coli and S. aureus*	MC3T3-E1	2021	[326]
ARX, CS and rGO sheets were combined and crosslinked using TEOS as a crosslinker	Cast into glass Petri dishes & dry at 50 ​°C in an oven.	*P. argenosa, S. aureus, E. faecalis, and E. coli*	MC3T3-E1	2021	[327]
ARX, CG, and rGO composites cross-linked them with TEOS	Cast into glass Petri dishes & dry at 55 ​°C in an oven.	*S. aureus, E. coli and P. aeruginosa*	Human red blood cells	2021	[328]
Arabinoxylan-functionalized-GO ​hydrogel with PVA and TEOS	Hydrothermal method	*S. aureus, E. coli and P. aeruginosa*	MC3T3-E1/Mouse	2022	[329]
Bacterial cellulose-functionalized-GO ​hydrogel with PVA, TEOS and curcumin release	Hydrothermal method	*S. aureus, E. coli and P. aeruginosa*	Not study/Anticancer against U87	2022	[330]
SA and GO covalently linked and crosslinked with TEOS	Solvothermal method	*E. coli, ​S. aureus ​and ​P. aeruginosa*	Pre-osteoblast (MC3T3-E1) cell line	2022	[331]
PDA-based platform composed of polyethyleneimine, pectin and PDA@Cu nanoparticles	One-step blended method	*E. coli and S. aureus*	L929 mouse fibroblast/Rat	2022	[332]

#### 3.3.1 Antibacterial scaffolds with antibiotics

**Fig. 11:**
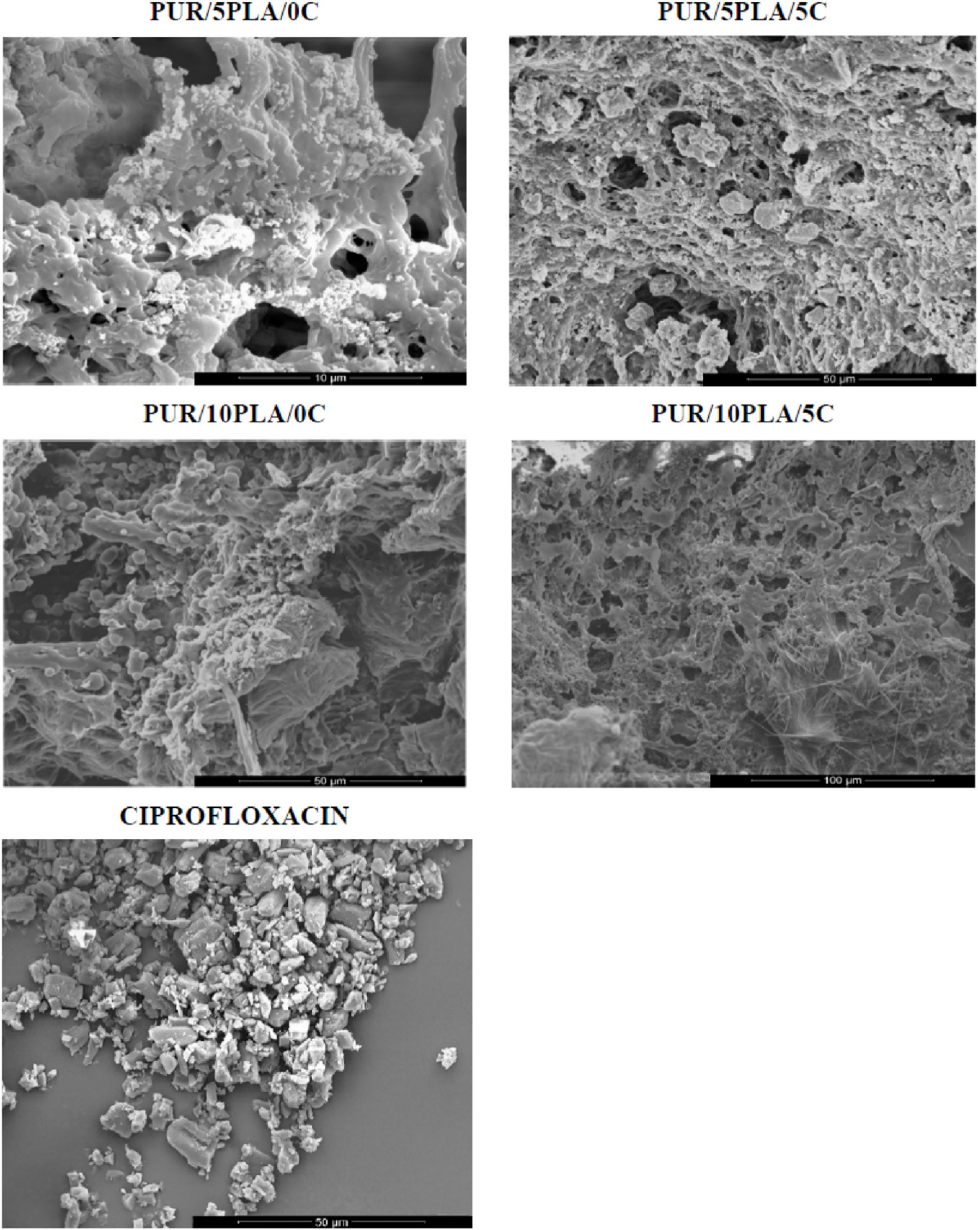
Scanning electron microscope (SEM) images of unmodified and Cipro-modified (2 or 5% *w/w*) HPPS, obtained by using 5 or 10% *w/w* of PLA and a SEM image of ciprofloxacin used for the scaffolds modification Reprinted with permission under a Creative Commons CC BY 4.0 License from Ref. [275]. Copyright 2020 MDPI.

Sustained, long-term and localized release of antibiotics loaded into scaffolds during fabrication is another strategy used to provide antibacterial activity for the early eradication of skin infections. PLGA electrospun scaffold containing CPFX delayed drug delivery by 24 ​h and showed an antibacterial effect toward *P. aeruginosa*, *S. aureus* and *S. epidermidis* [274]*.* The results indicated that physically adsorbed CPFX provided more antibacterial properties than CPFX blended with PLGA in the first 6 ​h, indicating that physisorption is a simple approach for a strong short-term antibacterial effect. In another study, Iga et al. developed a fast degradable hybrid porous scaffold modified with CPFX with different PU/PLA rates (Fig. 11). The resulting antibacterial scaffold showed suitable mechanical characteristics, morphology and degradation rate [275], while the antibacterial properties against *S. aureus* depended on the amount of ciprofloxacin added to the hybrid scaffolds but was not dependent on the PLA content.

Biodegradable scaffolds in the form of non-woven nanofibrillar matrices made of mixtures containing PCL and PLA and loaded with CPFX were obtained by jet-spraying [273]. The antibiotic release was efficient, inhibiting *E. coli* and *B. subtilis* growth, while showing good biocompatibility with dermal fibroblasts.

Many approaches to antibiotic delivery from scaffolds produce a burst release, but maintaining long-term inhibitory concentration is still a problem. Akkineni et al. prepared scaffolds based on alginate and methyl cellulose or alginate methylcellulose and Laponite by 3D printing to modulate the antibiotic release kinetics [272].

A biocompatible porcine acellular dermal matrix hydrogel blended with vancomycin has been developed for hemorrhage control, antibacterial action, and tissue repair in infected trauma wounds [276]. A patterned microstructural nanofibrous mats/gentamicin-loaded hydrogel composite scaffold has recently been proposed for skin tissue engineering [277]. The biocompatibility of the scaffold was proven by cytotoxicity and haemolysis studies.

#### 3.3.2 Scaffolds with metals/glass

**Fig. 12:**
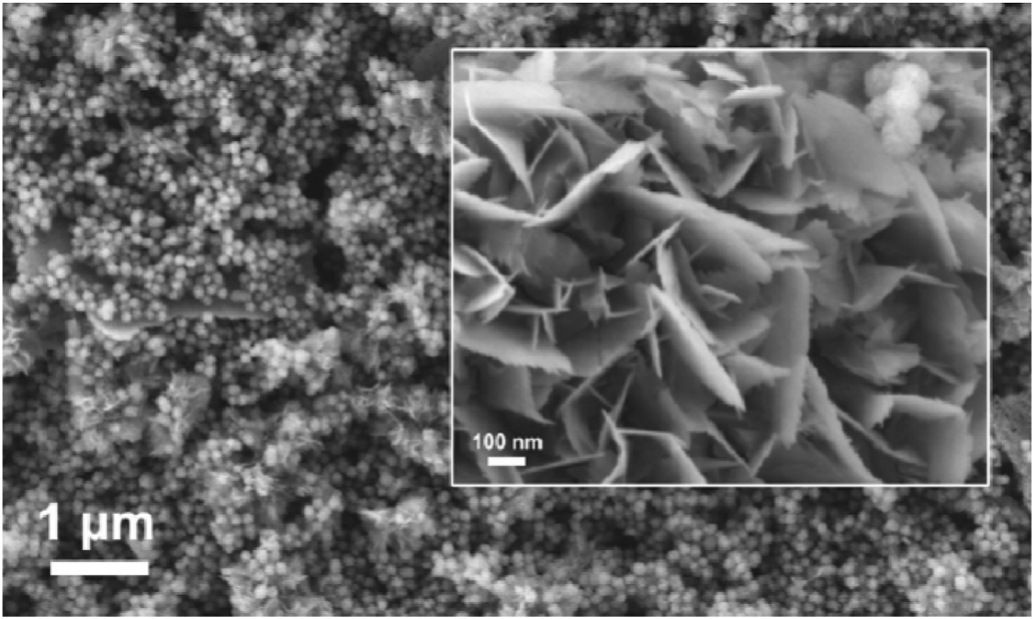
Scanning electron microscope images of Ag-MBGN after soaking in SBF for 14 days at different magnifications. Reprinted with permission under a Creative Commons CC BY License from Ref. [283]. Copyright 2019 Elsevier.

Scaffolds made of mesoporous bioactive glass nanoparticles modified with Ag (Ag-MBGN) were tested to check their antibacterial activity *in vitro* and in a 3D skin model for potential use in wound dressing [283] (Fig. 12).

Despite the good antibacterial activity obtained in *in vitro* tests, Ag-MBGN could not effectively inhibit *P. aeruginosa* in the 3D model that invaded deeper into the dermis, so that further research is necessary. Nonetheless, this is a promising scaffold for wound dressing due to its cytocompatibility and partial antibacterial capacity [283]. In another study, bioactive self-healing antibacterial injectable hydrogels based on bioactive glasses containing Cu^+2^ (Cu-BGs) as antibacterial agent were reported [289]. A double network of poly (ethylene glycol diacrylate) and SA containing Cu-BGs exhibited strong antibacterial activity over a wide range of bacteria. *In vitro* experiments showed that the self-healing hydrogels stimulated the viability, proliferation, and angiogenic capability of endothelial progenitor cells. *In vivo* studies demonstrated their efficiency in restoring blood vessels.

Aktürk et al. reported starch-coated silver nanoparticles (S-AgNPs) incorporated into a PVA matrix to fabricate nanofibers crosslinked with glutaraldehyde. These materials demonstrated an antibacterial effect against *E. coli* and *S. aureus* due to the release of silver nanoparticles, which were not toxic to HaCat keratinocytes and human epidermal keratinocytes and so were promising for wound dressing applications [284]. Another study with PVA/Starch scaffolds containing AgNPs confirmed the results and indicated good properties such as biodegradability, biocompatibility, and antibacterial activity against *P. aeruginosa* [285]. Alternative Ag-based scaffolds for tissue engineering with good antibacterial activity against *E. coli, S. aureus, P. aeruginosa*, and *C. albicans* have also been developed [279,288]*.* PCL/gelatin (Ge) nanofibrous scaffolds coated with silver (PCL/Ge/Ag) were non-toxic to cells and demonstrated antibacterial capacity against *B. cereus* and *E. coli* [280]. PCL scaffolds containing different ratios of calcium peroxide with or without ascorbic acid exhibited antimicrobial capacity and were not toxic [278]. In another study, a PCL/poly (glycerol sebacate) (PGS) scaffold with calcium peroxide provided sustainable oxygen release for from several days to a week and showed good antibacterial activity [286]. *In vivo* experiments have demonstrated that PCL nanofibrous mat containing silver sulfadiazine as an antibacterial compound can be considered a powerful wound dressing because of its effects on skin tissue repair and remodeling, plus its antibacterial capacity against both Gram-positive and Gram-negative bacteria [287]. Fibrous PCL scaffolds containing Ag-doped magnetite nanoparticles were reported by Anhmed et al. The roughness and hydrophilicity of the polymeric nanofibers were modified by the Ag-doped nanoparticles, which showed positive results on cell adhesion and growth. Both the viability of human melanocytes and the antibacterial performance (against *E. Coli* and *S. aureus*) increased with the concentration of Ag in the magnetite nanoparticles. *In vivo* results demonstrated that skin wound healing in rats also increases monotonically with the concentration of Ag in the magnetite phase [290].

In a recent study, biocompatible nanobiocomposite scaffolds were engineered based on crosslinked lignin-agarose hydrogel, silk fibroin, and zinc chromite (ZnCr_2_O_4_) nanoparticles as antibacterial agents [291]. Toxicity was less than 13% with a good antibacterial activity, preventing the formation of *P. aeruginosa* biofilm. *In vivo* experiments showed that wounds in mice treated with these nanobiocomposite hydrogels were completely healed in five days.

Effective hemostasis and antibacterial activity are the urgent challenges for deep, narrow, irregular or non-compressible wounds. Ai et al. prepared a 3D printed injectable wound-cooling hemostatic system based on SA/SiO_2_ with the addition of Ag nanoparticles [281]. The hydrogel showed good biocompatibility and a robust antibacterial capacity against *E. coli, P. aeruginosa, S. aureus* and MRSA*.* The *in vivo* test on a femoral artery injury model showed a rapid hemostatic response.

In a new approach, Pérez-Diaz et al. developed a biomatrix based on radiosterilized pig skin (RPS) as a carrier to deliver MSCs into wound beds. In addition, AgNPs were incorporated into the biomatrix at different concentrations [282]. The nanocomposites showed antibiofilm properties with samples containing 250 and 1000 ​ppm of AgNPs, although MSCs survived and proliferated on the nanocomposites impregnated with up to 250 ​ppm of AgNPs.

Even though zinc-based material scaffolds have been studied much less than silver-based ones, these alternative approaches with zinc are very promising for skin tissue engineering applications. For example, gelatin-based and Zn^2+^-incorporated composite hydrogels have been developed for accelerated infected wound healing [292]. This hydrogel exhibited sustainable release behavior of Zn^2+^ with good biocompatibility toward NIH-3T3 cells and strong antibacterial abilities against *E. coli* and *S. aureus.*

#### 3.3.3 Scaffolds with antibacterial polymers/peptides

Chitosan is an ideal biopolymer for tissue engineering because of its antibacterial properties, biocompatibility, control of inflammatory mediators, ability to aid in faster healing and ability to regulate coagulation [295,297,333,334]. PCL scaffolds with CS coverage demonstrated cell adhesion and viability as well as a slower bacterial growth rate toward *S. aureus* and *S. epidermis* [294]*.* When treating wounds in rat liver, 3D porous sponges with quaternary chitin/partially deacetylated chitin nanofibers as their skeleton (QCNS) outperformed traditional hemostatic agents (gauze, gelatin sponge, and Celox™). QCNS were shown to be an excellent hemostatic dressing for noncompressible wounds because of its excellent cytocompatibility, hemocompatibility, and antibacterial activity [293,296].

Scaffolds that include peptides as antibacterial agents have also been reported for skin tissue engineering and wound healing. Kandhasamy et al. developed silk fibroin electrospun fiber mats containing antibacterial Vitamin K3 carnosine peptides for diabetic wound healing applications [299]. The fiber mats presented good biodegradability, adhesiveness and sustained drug release. Human HFF1 and NIH 3T3 fibroblasts were tested for cell viability and antibacterial performance against *S. aureus*, *E. coli* and *P. aeruginosa*. The mats’ diabetic wound curative capacity *in vivo* was tested in male diabetic Sprague Dawley rats. The results showed that the fiber mats with peptides promoted wound healing in a shorter time than those without peptides. In a similar approach, a cationic antimicrobial peptide (CM11 peptide) was loaded into silk fibroin/gelatin bilayer sponges as wound dressing [300]. The sponges loaded with the peptide showed a controlled release without cytotoxicity on human foreskin fibroblasts (Hu02 ​cell line) and a significant antibacterial performance against Gram-positive and Gram-negative bacteria.

#### 3.3.4 Scaffolds with carbon nanomaterial

Graphene nanosheets can be used as reinforcement and cell-instructive materials in soft tissue scaffolds [335]. Scaffolds with carbon nanomaterials embedded within the polymeric matrix have also been proposed for skin tissue engineering and wound healing. Nanofibrous PHBV/collagen with GO (0.3% *w/w*) as an antibacterial agent was prepared for wound coverage [301]. The incorporation of collagen and GO reduced the diameter of the nanofibers and increased porosity. The nanofibers showed enhanced cell proliferation (3 T3-L cell line) and antibacterial capacity against *E. coli* and *S. aureus*. In another study, Thangavel et al. prepared a nanocomposite dressing based on isabgol, a natural carbohydrate polymer, and rGO nanoparticles for enhanced vascularization and wound healing using normal and diabetic rats as models [302]. Isabgol/rGO scaffold dressing showed good biocompatibility and antibacterial activity. rGO made the wounds in the Wistar rats shrink and cut down on the time it took for the wounds to heal, which means that this method could speed up the healing of both normal and diabetic wounds.

In another approach, an antibacterial PU-modified GO composite was developed with a skin-like bilayer structure for wound healing applications [303]. The results of tests on a mouse model showed there was a big difference in how quickly the wounds healed. This could be because of the free-bacteria environment and re-epithelization during healing, both brought about by the engineered membrane.

Zmejkoski et al. recently reported a novel hydrogel composite based on bacterial cellulose impregnated with graphene quantum dots (GQDs) for wound healing treatment [304]. The cellulose polymeric matrix was loaded with ca. 12% of GQDs. The hydrogel composites were biocompatible and showed robust antibacterial performance against Gram-positive and Gram-negative bacteria, also good wound fluid absorption and water retention, which indicate their potential in wound healing applications.

Hurtado et al. recently reported a new biodegradable semi-interpenetrated polymer network of PHBV and calcium alginate to provide an alternative strategy to enhance the poor adhesion properties of calcium alginate [305]. These hydrogels were also synthesized with the addition of 10% *w*/*w* of graphene nanoplatelets (GNPs), which had no cytotoxic effect on human keratinocytes and provided superior antiviral activity against a surrogate viral model of SARS-CoV-2. However, the antibacterial activity of these hydrophilic materials has so far not been studied.

#### 3.3.5 Scaffolds produce by combined strategies and alternative methods

Several studies explored the combination of antibacterial polymers and antibiotics. Collagen/CS scaffolds loaded with CPFX were prepared by freeze-drying. The scaffolds were highly biocompatible (fibroblast cells) and efficient against *E. coli* and *S. aureus* [320]. In a new approach, collagen/CS oxygenating scaffolds loaded with the same antibiotic were prepared with calcium peroxide as a chemical oxygen source [321]. Oxygen-producing biomaterials have been shown to promote wound healing. In this study, both oxygen and drugs showed a sustained release. *In vitro* cultures indicated that the scaffold had suitable cell adhesion and fibroblast migration and robust antibacterial activity. *In vivo* studies performed on a rat skin flip model showed better wound healing and less necrosis, indicating the promising potential of this strategy.

Some antibacterial scaffolds are produced by combining antibacterial polymers with metals. A chitosan scaffold covered with different metals such as selenium or silver demonstrated antibacterial activity against *S. aureus, E. coli* and MRSA, while Ag-CS scaffolds showed also cytocompatibility towards fibroblasts [308,319].

Halloysite nanotube (HNT)-reinforced alginate-based nanofibrous scaffolds loaded with cephalexin (CEF) delayed drug delivery by 7 days and showed antibacterial effects against Gram-positive and Gram-negative bacteria [314].

Other strategies consist of developing scaffolds with natural antimicrobial extracts. For example, Radhika et al. extracted collagen and gelatin from *Priacanthus humrur* skin [41]. They created an antibacterial collagen/gelatin/CS porous novel scaffold by freeze drying and subsequent crosslinking of polymers. In another study, CS matrices were loaded with grape seed extract or royal jelly to produce scaffolds with antimicrobial, anti-inflammatory and wound healing capabilities [309] cytocompatible with human lung fibroblast. It should be noted that honey in hydrogels and cryogels also reduces bacterial adhesion [307,315]. Biocompatible PU and PLA polymer scaffolds are commonly proposed for skin tissue engineering [336]. Other polymer scaffolds such as PU, PLA, PLGA, PEG-DA or PHA have been investigated with natural extracts for tissue engineering, particularly for skin, oral tissue, and cardiac regeneration [289,306,337]. The incorporation of cellulose acetate (CA) within the PLA matrix enhanced the physicochemical properties of the scaffolds. The scaffold exhibited promising results in *in vivo* wound healing assays and antibacterial activity against both *S. aureus* and *E. coli* [316]. Çakir et al. demonstrated that silk fibroin sponges with heparin and silver sulphadiazine can mimic the physical structure of natural skin tissue. These scaffolds showed an antibacterial effect against Gram-positive bacteria [311]. Silk fibroin/CS scaffolds exhibited excellent biocompatibility and antibacterial capacity against *S. aureus* and *E. coli* [318]*.*

Antibacterial scaffolds made of quaternary ammonium organosilane (QOS) collagen nanofibers increased the flexibility of rigid collagen nanofibers and had good properties like thermal stability, antibacterial activity and stimulated cellular growth and proliferation, and are therefore very promising for effective use as an interactive wound dressing material [312]. PLA modified with l-ascorbic acid or fumaric acid *via* a plasma treatment method changed the surface morphology and topography, so that the scaffold showed antibacterial capacity against Gram-positive and Gram-negative bacteria [317]. In another combined strategy, PHA with graphene-decorated silver nanoparticles have shown antibacterial properties against Gram-positive and Gram-negative bacteria [310]. The antibacterial activity lasts for up to 24 ​h of incubation, which is a factor to consider for effective wound dressings. Graphene-silver nanocomposites (rGO-Ag) with an antimicrobial peptide (polylysine) have been reported. The peptide functionalization of the rGO-Ag nanocomposites increased the antibacterial efficacy against *S. aureus* biofilm and reduced the dissolution of Ag ions and *in vitro* toxicity. The *ex vivo* rat disinfection model was shown to be capable of eliminating biofilm formation and disruption [322].

Graphene foams have also been proposed as carriers of metal ions against antibiotic-resistant bacteria, with a special potential for wound dressing applications [323]. Another strategy consists of CS/PVA/GO based pH-responsive composite hydrogels crosslinked with TEOS and produced by solution casting for wound healing [326], showing antibacterial activity against Gram-positive and Gram-negative pathogens and good biocompatibility. Biopolymer-based composite hydrogels with antibacterial and antitumor properties were made from sodium alginate (SA) and GO covalently linked and crosslinked with tetraethyl orthosilicate (TEOS) by the solvothermal method [331].

Antibacterial, degradable and pH-responsive CS/guar gum/PVA blended hydrogels with different crosslinking amounts of TEOS for wound dressing [325]. The antimicrobial study revealed that these composites are highly antibacterial against Gram-positive (*S. aureus* and *Bacillus cereus*) and Gram-negative (*P. aeruginosa and E. coli*) bacterial strains. ARX-based nanocomposite hydrogels functionalized into carboxymethylarabinoxylan (CMARX) with different amounts of TEOS loaded with the chemotherapeutic agent Fluorouracil (5FU) onto rGO showed antibacterial activity against *S. aureus* and *P. aeruginosa* and anticancer activity against Uppsala 87 Malignant Glioma (*U-87*) cells [324]. ARX, CG, and rGO composites cross-linked with the optimal amount of TEOS were shown to be hemocompatible, pH-responsive and broad spectrum antibacterial, thus very promising for sustained drug release for skin wound care and treatment [328]. ARX, CS and rGO sheets were combined and crosslinked using TEOS as a crosslinker to produce smart pH-sensitive biocompatible composite hydrogels with broad-spectrum antibacterial activity for wound healing [327]. Multifunctional hydrogels based on ARX-functionalized-GO and bacterial cellulose-functionalized-GO using the hydrothermal method through cross-linking GO-arabinoxylan and PVA with TEOS recently prevented infections (*E. coli*, *S. aureus*, and *P. aeruginosa*) and were thus shown to be promising for wound healing [329,330]. Advanced hydrogels based on arabinoxylan-functionalized-GO promoted wound healing *in vitro* and *in vivo* [329]. The advanced hydrogels based on bacterial cellulose-functionalized-GO showed potential anticancer activity against U87 ​cells and could be used for the controlled release of curcumin [330].

A simple and safe PDA-based photothermal platform has recently been developed for photothermal antibacterial therapy in wound healing [332]. This biocompatible platform composed of polyethyleneimine, pectin and polydopamine@Cu nanoparticles showed a highly efficient bacteria-killing ability.

### 3.4 Antibacterial scaffolds for oral regeneration

Maxillofacial defect restoration is a great challenge due to the complicated pre-existing anatomy of the skull [338], for which new strategies for oral tissue regeneration using antimicrobial scaffolds have been developed (See Table 3).

**Table 3:** Antibacterial Scaffolds for oral tissue regeneration.

Material	Fabrication method	Bacteria	Non-toxicity: cell line	Year	Ref
Scaffolds with antibiotics/antiseptics					
PDS scaffold loaded with metronidazole	Root canal space	*P. gingivalis*	Not studied	2012	[339]
Bimix antibiotic-containing polydioxanone-based polymer scaffolds	Electrospinning	*E. faecalis P. gingivalis and F. nucleatum*	Human dental pulp stem cells	2014	[340]
PDS ​+ ​MET/CPFX scaffolds	Electrospinning	*E. faecalis*	Human dental pulp stem cells	2015	[341]
TAP-mimic polymer nanofibrous	Electrospinning	*P. gingivalis*	Not studied	2016	[342]
Chlorhexidine-releasing HAp scaffold incorporated with human serum albumin nanoparticles	Desolvation method	*S.mutans*	Not studied	2020	[343]
**Scaffolds with antibacterial polymers**					
Chitosan-based polyelectrolyte complex scaffolds	Electrostatic crosslinking	*S. aureus and E. coli*	MC3T3-E1preosteoblast cells	2012	[344]
Chitosan based scaffold	Freeze drying	*P. gingivalis and S. mutans*	human gingival epithelial cells	2020	[40]
**Scaffolds with metals**					
PCL scaffold loaded with Ag_3_PO_4_ and lidocaine	3D printing	*S. aureus and E. coli*	MC3T3-E1preosteoblast cells	2019	[345]
Barium titanate reinforced polyvinyl-siloxane scaffolds	Commercial	*S. epidermis*	Not studied	2020	[346]
COL, chondroitin 4-sulfate, fibronectin and silver NP	Freeze-drying	*F. nucleatum, P. gingivalis*	Gingival fibroblasts, THP-1 monocytes/Chicken eggs	2021	[347]
**Scaffolds with bioglass and antiseptics/metals**					
Bioglass/chitosan scaffolds with chlorhexidine gluconate	Freeze-drying	*E. faecalis*	Wistar-Furth rat	2020	[348]
Nanometric Zinc doped bioactive glass	Sol-gel method	*A. actinomycetemcomitans, P. gingivalis and P. intermedia*	Not studied	2020	[349]
**Antibacterial scaffolds by other strategies**					
Epigallocatechin gallate scaffold	Crosslinking	*Not specified*	Human dental pulp stem cells	2017	[350]
Chitosan, calcium phosphate and GO	Blending	*E. faecalis*	Human dental pulp stem cells	2021	[351]
Carrageenan Based Injectable Hydrogel with *Cissus quadrangularis* extract	Solvent casting	Not studied	Not studied	2022	[352]

Scaffolds with antibiotics have been proposed for oral tissue regeneration using a combination of metronidazole and CPFX [340]. Their antimicrobial activity has been confirmed against *Enterococcus faecalis, Porphyromonas gingivalis*, and *Fusobacterium nucleatum.* MET/CPFX scaffolds enhanced the viability and proliferation of dental pulp stem cells [339,341]. Albuquerque et al. reported an electrospun antibacterial scaffold prepared with polydioxanone (PDS) nanofibers loaded with TAP (CPFX, metronidazole, and minocycline) against P. *gingivalis*-infected dentin biofilm. The results indicated the potential of these nanofibrous scaffolds for intracanal disinfection before regenerative endodontics [342]. A novel antibacterial HAp scaffold against *S. mutans* has been developed by immobilizing chlorhexidine (CHX)-loaded human serum albumin (HSA) nanoparticles on its surface *via* surface charge interaction [343].

A biopolymer made of a polyelectrolyte complex (PEC) composed of CS, γ-polyglutamic acid (γ-PGA) and carboxy-methyl-cellulose (CMC) was developed to fabricate dental scaffolds [344]. These PEC scaffolds showed biocompatibility and antibacterial activity against *E. coli* and *S. aureus*. In another study, Li et al. reported the antibacterial activity of a non-cross-linked CS scaffold against typical oral pathogens such as *Porphyromonas gingivalis* and *Streptococcus mutans* [40]*.* These scaffolds had good physical and biological properties such as biodegradability, physical stability and biocompatibility. Polyvinyl-siloxane (PVS) is a suitable material to prepare dental scaffolds because it is biocompatible, it can be modeled and can be produced with higher resistance to colonization to prevent bacterial infections by adding BaTiO_3_ to it [346]. The engineered scaffolds based on this approach increased the beneficial antibacterial capacity against *S. epidermis* by 25%.

Other researchers have developed scaffolds using metal ions, particularly silver, for example, Ag_3_PO_4_-lidocaine-loaded-PCL scaffolds using pneumatic extrusion-based 3D printing were developed by Shao et al. [345]. The scaffolds demonstrated both antibacterial and analgesic activity in addition to cytocompatibility, which depended on the lidocaine and Ag concentrations. Following this strategy, 3D hybrid scaffolds consisting of extracellular matrix components, collagen, chondroitin 4-sulfate, and fibronectin, functionalized with AgNPs were prepared to improve periodontitis treatments [347].

Dental scaffolds with drug delivery capability, such as hematite-doped bioglass/CS scaffolds with CHX were investigated for the repair of infected root canals. They were also found to have osteoinduction capacity [348] and bacterial growth of *E. faecalis* was eliminated after 14 days. In a different strategy, epigallocatechin gallate (EGCG) was used as an antibacterial cross-linking agent in hydrogel collagen scaffolds to promote proliferation and differentiation of human dental pulp cells (hDPCs) while impeding bacterial infections [350].

Novel approaches based on carbon nanomaterials have also been reported. Wu et al. proposed a new antibacterial scaffold with GO and calcium phosphate incorporated in a CS hydrogel [351]. The antimicrobial scaffold proved to be effective in preventing *E. faecalis* biofilm and also had good biocompatibility to support human dental stem cell attachment.

Novel carrageenan-based injectable hydrogel scaffolds containing *Cissus quadrangularis* extract have shown biocompatibility and antioxidant activity for facilitating dentin-pulp complex regeneration [352].

### 3.5 Antibacterial scaffolds for muscle, nerve, trachea, cardiac and other tissue engineering applications

A variety of scaffolds (sometimes combined with stem cells) have been developed and optimized for muscle, nerve, trachea, cardiac and other tissue engineering applications [[353], [354], [355], [356]]. However, introducing foreign bodies into the human body increases the risk of bacterial infection. Despite disinfection procedures, there is a risk of contamination by pathogens that can cause infections during surgical interventions. Bacterial infections can appear long after surgery and can be responsible for implant failure and distress to patients, reducing their quality of life [357].

Research on antimicrobial scaffolds is focused mainly on bone, skin, and oral tissue applications. However, different approaches to developing antimicrobial scaffolds for other tissues such as muscle, nerve, cardiac or trachea have been proposed. This section reports on general strategies with antimicrobial scaffolds or other strategies for other types of tissue not included in the previous sections (Table 4).

**Table 4:** Antibacterial Scaffolds for muscle, nerve, trachea and other tissue engineering applications.

Material	Fabrication method	Application	Bacteria	Non-toxicity: cell line/animal model	Year	Ref
**Scaffolds with antibiotics**						
TCH-loaded PLLA/PLLA-poly (ethylene glycol)-NH2	Electrospinning	Tissue engineering	*S. aureus*	Not studied	2011	[358]
PCL and Cefazolin	3D printing, salt-leaching	Tissue engineering	*S. aureus*	3T3 fibroblast-like cells	2018	[359]
Recombinant spider silk proteins, silica NPs, gentamicin, neomycin, kanamycin	Casting, 3D printing	Tissue engineering	*E. coli*	BALB/3T3 fibroblast-like cells	2020	[360]
**Scaffolds with metals/ceramics/glass**						
Nanofiber webs of CS/poly (vinyl alcohol) blends incorporated with silver nanoparticles	Electrospinning	Tissue engineering	*E. coli*	Not studied	2011	[361]
PCLA-nAg Nanofibrous Composite	Electrospinning	Tissue engineering	*S. aureus*	Human MSCs	2012	[115]
CuO-nanofibrillar cellulose/glycerol based hyperbranched epoxy nanocomposite	Electrospinning	Muscle	*S. aureus and E. coli*	L6 muscle cells	2015	[353]
Poly (methyl methacrylate) coating modified with silver nanoparticles to an aluminium alloy	*In situ* polymerization	Tissue engineering	*P. aeruginosa*	Not studied	2018	[362]
Silver-zeolite coatings on 3D printed porous stainless steels	3D printing (selective laser melting)	Tissue engineering	*E. coli and S. aureus.*	BMSCs	2020	[363]
PVA-Ag and CS-Ag nanocomposites	Augmentation technique	Tissue engineering	*E. coli, S. aureus, S. epidermidis and K. pneumoniae*	Huh-7 liver cells	2020	[364]
F127–CHO micelle crosslinked by polydopamine NPs and gold nanoparticles	Freeze drying	Muscle	*E. coli, S. aureus*	C2C12 myoblast cells/Rat	2021	[365]
**Scaffolds with antibacterial polymers/peptides**						
Hydrogels based on CS-graft-aniline tetramer and dibenzaldehyde-terminated poly (ethylene glycol)	sol-gel technique	Tissue engineering	*E. coli, P. aeruginosa S. aureus*	L929 mouse fibroblast cells	2010	[366]
PHMB/polyacrylic acid/PHMB-coated scaffold	Layer by layer assembly	Tissue engineering	*E. coli*	Fibroblast cells	2012	[367]
Quaternized CS-graft-polyaniline/oxidized Dextras	Crosslinking	Muscle, cardio, nerve	*E. coli and S. aureus*	ADMSCs/Rat	2015	[354]
CS Poly (lactic acid) nanofibers	Electrospinning	Tissue engineering	*E. coli*	L-929 mouse fibroblast cells	2015	[118]
CS-graf t-aniline tetramer	Electrospinning and self-healing	Muscle	*S. aureus and E. coli*	ADMSCs and C2C12 myoblasts/Rat	2016	[368]
PS-b-Polyacrylic acid and PS-*b*-PDMAEMA	3D printing	Tissue engineering	*S. aureus*	Not studied	2017	[369]
PCL scaffold	electrospinning, rotary jet spinning and AB	Tissue engineering	*S. aureus and P. aeruginosa*	hFOB osteoblast cells	2018	[370]
MWCNT/PPy/Pd nanocomposite	Chemical oxidation polymerization	Tissue engineering	*B. subtilis, P. aeruginosa, K. pneumoniae and E. coli*	Human osteosarcoma cells	2018	[371]
NO-Releasing Alginates	Chemical modification	Tissue engineering	*P. aeruginosa, S. aureus, B. cepacia and MRSA*	Not studied	2019	[372]
RGD-based hydrogelator and polyaniline	Gelation	Cardiac	*E. coli, S. epidermidis*	3T3 fibroblast-like cells	2021	[373]
**Scaffolds with carbon nanomaterials**						
Electroactive collagen with reduced graphene oxide	Lyophilization	Cardiac	*E. coli, S. aureus and S. pyongenes*	HUVEC human endothelial cells	2019	[374]
PLA/GO and IL	Electrospinning and 3D printing	Trachea	*E. coli and S. aureus*	L929 mouse fibroblasts/Rabbit	2019	[355]
Pd/PPy/rGO nanocomposite	Oxidative polymerization method	Tissue engineering	*E. coli, B. subtilis, P. aeruginosa, and K. pneumoniae*	Saos-2 osteoblast-like cells	2020	[375]
**Scaffolds produce by combined strategies and alternative methods**						
Urinary bladder (UBM-ECM) and liver (L-ECM)	No	Tissue engineering	*S. aureus and E. coli*	Not studied	2006	[116]
Boron Nitride Doped Polyhydroxyalkanoate/CS Nanocomposite	Solvent casting	Tissue engineering	*E. coli and S. aureus (MRSA)*	HaCat keratinocyte cells	2019	[117]
Two-dimensional molybdenum disulphide nanoparticles encapsulated in polyhydroxyalkanoate and CS	Solvent casting	Tissue engineering	*E. coli and methicillin-resistant Staphylococcus aureus*	HaCat keratinocyte cells	2020	[114]
Polylactic acid/cellulose acetate with 1-chloro-2,2,5,5-tetramethyl-4-imidazolidinone	3D printing	Tissue engineering	*E. coli and S. aureus*	Not studied	2020	[376]
HAp and essential oil (Nigella sativa)	Grafting	Muscle	*S. aureus*	C2C12 mouse myoblast cells	2021	[377]
3D-printed HDPE scaffolds with bioactive and antibacterial layer-by-layer	3D Printing and surface modification	Auricle reconstruction	*E. coli and S. aureus*	L-929 mouse fibroblast cells/Rat	2022	[378]

Different strategies have been reported based on scaffolds loaded with antibiotics for general tissue engineering applications. Chen et al. developed polylactic acid/poly (ethylene glycol) (PLA/PEG) scaffolds to deliver multiple biomolecules (including growth factors) and drugs for wound dressing, periodontal membranes, or more complicated tissues in which growth factors and anti-infection precautions are critical. The scaffolds were assessed by loading the model drug TCH. These scaffolds reduced the activity of *S. aureus* [358]. Visscher et al. reported dual macro/micro porous scaffolds prepared by combining 3D printing with the traditional salt-leaching technique [359]. This antibacterial platform was evaluated for the local release of the antibiotic Cefazolin, loaded *via* a solution drop-loading technique, had no cytotoxic effects on 3 T3 fibroblasts and did not cause *in vitro* blood clots. In another recent approach, composites consisting of recombinant spider silk proteins and mesoporous silica nanoparticles loaded with specific antibiotics and antimycotics showed antimicrobial activity over 15 days. 2D films and scaffolds, prepared by 3D printing, exhibited good biocompatibility, promoting cell adhesion and proliferation [360].

Scaffolds with antibacterial metals have also been proposed as a general approach for tissue engineering [115,[361], [362], [363], [364]] or for specific applications, such as muscle [353,365]. A CuO nanoparticle decorated biobased hyperbranched epoxy/CuO-nanofibrillar cellulose nanocomposite scaffold was prepared to acquire efficient antimicrobial activity for smooth muscle cell regeneration [353]. *In situ* injectable hydrogel has the advantage of being able to match the shape of the damaged tissue and reduce patients’ distress with a minimally invasive method [379]. Ge et al. engineered a conductive, antioxidative, and antibacterial hydrogel with oriented channels to enhance skeletal-muscle regeneration [365]. Biometal gold@dopanime nanoparticles were incorporated as an antibacterial agent. *In vitro* experiments in C2C12 murine myoblasts showed that these advanced materials could promote myotube formation. *In vivo* assessment, performed on a rat tibialis anterior muscle defect model, showed that these scaffolds facilitated skeletal muscle regeneration.

**Fig. 13:**
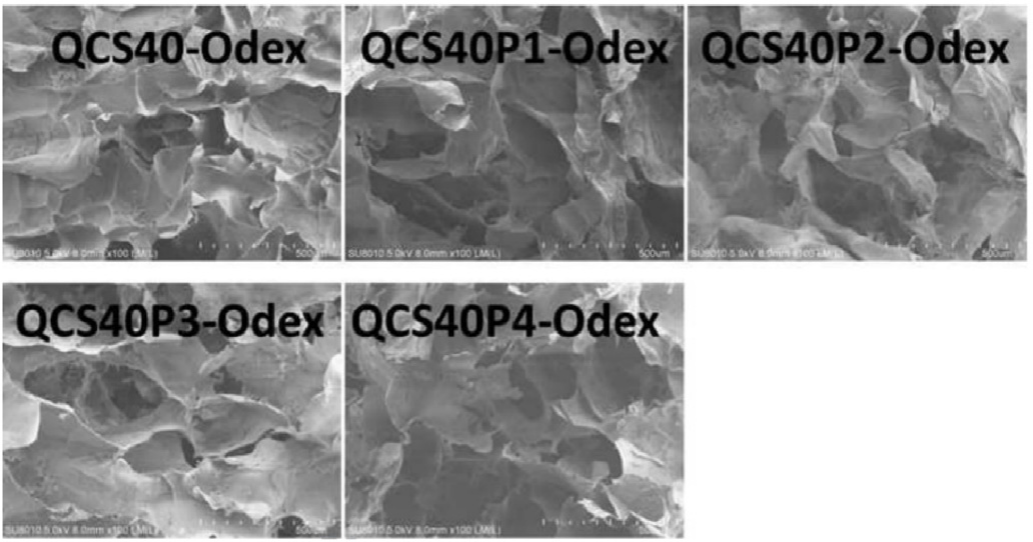
Hydrogel morphologies after swelling in phosphate buffered saline. Scale bar was 500 ​μm. Reprinted with permission from Ref. [354]. Copyright 2015 Elsevier.

Scaffolds with antibacterial polymers have been proposed as a general strategy for tissue engineering [118,366,367,[369], [370], [371], [372]] or for specific applications [354,368]. Zhao et al. proposed an *in situ* forming antibacterial conductive degradable hydrogel employing quaternized chitosan (QCS) and grafted polyaniline (PANI) with oxidized dextran as a crosslinker for electrical signal-sensitive tissues, such as muscle, cardiovascular, and nerve [354] (Fig. 13).

The introduction of PANI into the QCS copolymer significantly reduced its cytotoxicity, greatly stimulated the proliferation of C2C12 ​cells and improved its antibacterial activity, especially the QCS40P3-Odex hydrogel with a killing percentage of up to 95% [354].

Injectable hydrogels have also been studied for cell delivery therapy in myocardial [368] and bladder regeneration [5]. A self-healable conductive injectable hydrogel made of chitosan-graft-aniline tetramer (CS-AT) and dibenzaldehyde terminated poly (ethylene glycol) (PEG-DA) as cell delivery platform showed very promising results for myocardial infarction [368]. The resulting hydrogel not only does not cause cytotoxicity but also shows antibacterial activity against *E. coli* and *S. aureus* and is also capable of producing good targeted cell release *in vivo* [368]. A conductive peptide-PANI composite hydrogel with antimicrobial activity that can bind to deoxyribonucleic acid (DNA) was recently reported [373]. The hydrogel supported the organization of cardiomyocytes into a spontaneously contracting system and demonstrated antibacterial activity against *E. coli* and *S. epidermidis*.

In another approach, Ghannadian et al. compared different fabrication techniques to prepare PCL scaffolds by electrospinning, rotary jet spinning, and airbrushing (AB) for the treatment of musculoskeletal defects without infections. The products of AB significantly reduced bacterial surface colonization of Gram-positive and Gram-negative bacteria [370].

**Fig. 14:**
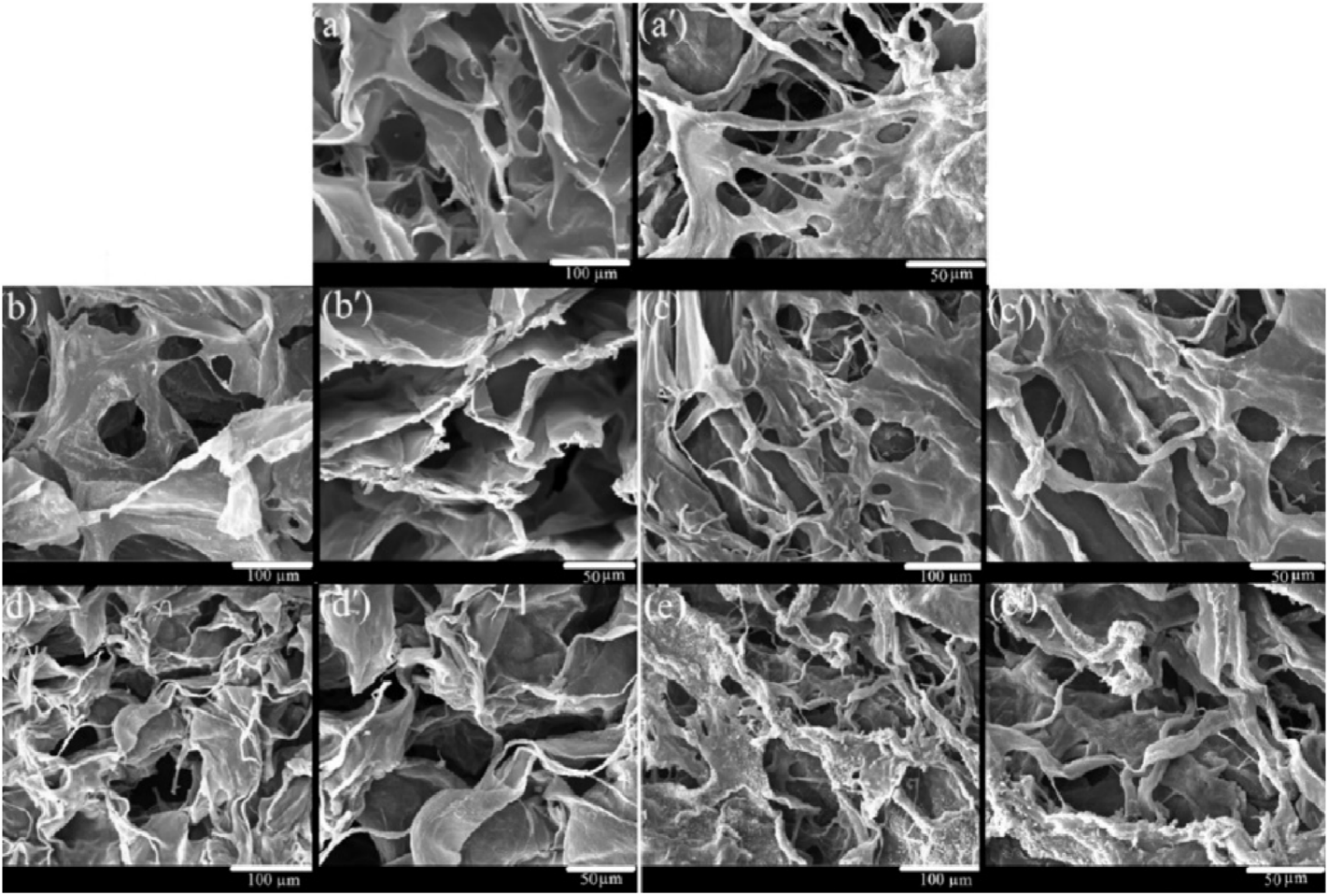
Scanning electron microscope images of scaffolds of different magnifications: (**a**) and (**a′**) Collagen, (**b**) and (**b′**) Collagen-rGO-200, (**c**) and (**c′**) Collagen-rGO-400, (**d**) and (**d′**) Collagen-rGO-600 and (**e**) and (**e′**) Collagen-rGO-800. Reprinted with permission from Ref. [374]. Copyright 2019 Elsevier.

Graphene-based nanomaterials are promising compounds for cardiac tissue engineering due to their excellent electrical and mechanical properties [380]. Collagen patches charged with different concentrations of rGO have been developed to achieve good long-term cardiac regeneration [374] (Fig. 14**)**.

The results showed that the rGO coating significantly improved the mechanical properties, electroactivity of the collagen scaffolds and the viability of human umbilical vein endothelial cells (HUVEC) in a concentration-dependent manner. The antibacterial properties of the Collagen-rGO scaffolds against *Escherichia coli*, *S. aureus*, and *Streptococcus pyogenes* were confirmed by field emission scanning electron microscopy [374].

These results indicate that the rGO coating has promising properties for collagen scaffolds that provide a desirable microenvironment for the regeneration of vascular tissue. In another approach, a biocompatible tissue-engineered trachea was developed with electrospun patterned PLA/GO and IL fibrous membranes with synergistic antibacterial properties [355].

Scaffolds produced by combining strategies and alternative methods have also been reported for several tissue engineering applications [114,116,117,376,377]. Zuo et al. prepared a stable 3-dimensional printed polylactic/cellulose acetate scaffold with the antimicrobial agent 1-chloro-2,2,5,5-tetramethyl-4-imidazolidinone (MC) for biomedical applications and food packaging [376]. Essential oils of aromatic foliage have also been proposed both as bioactive and biocide agents. Amma et al. incorporated Nigella sativa essential oil into a biogenic scaffold [377]. The quinine constituent of N. sativa has been reported to stop microbial growth. The scaffold, prepared by grafting HAp and the essential oil, enhanced myoblast differentiation and antibacterial activity against *S. aureus*.

**Fig. 15:**
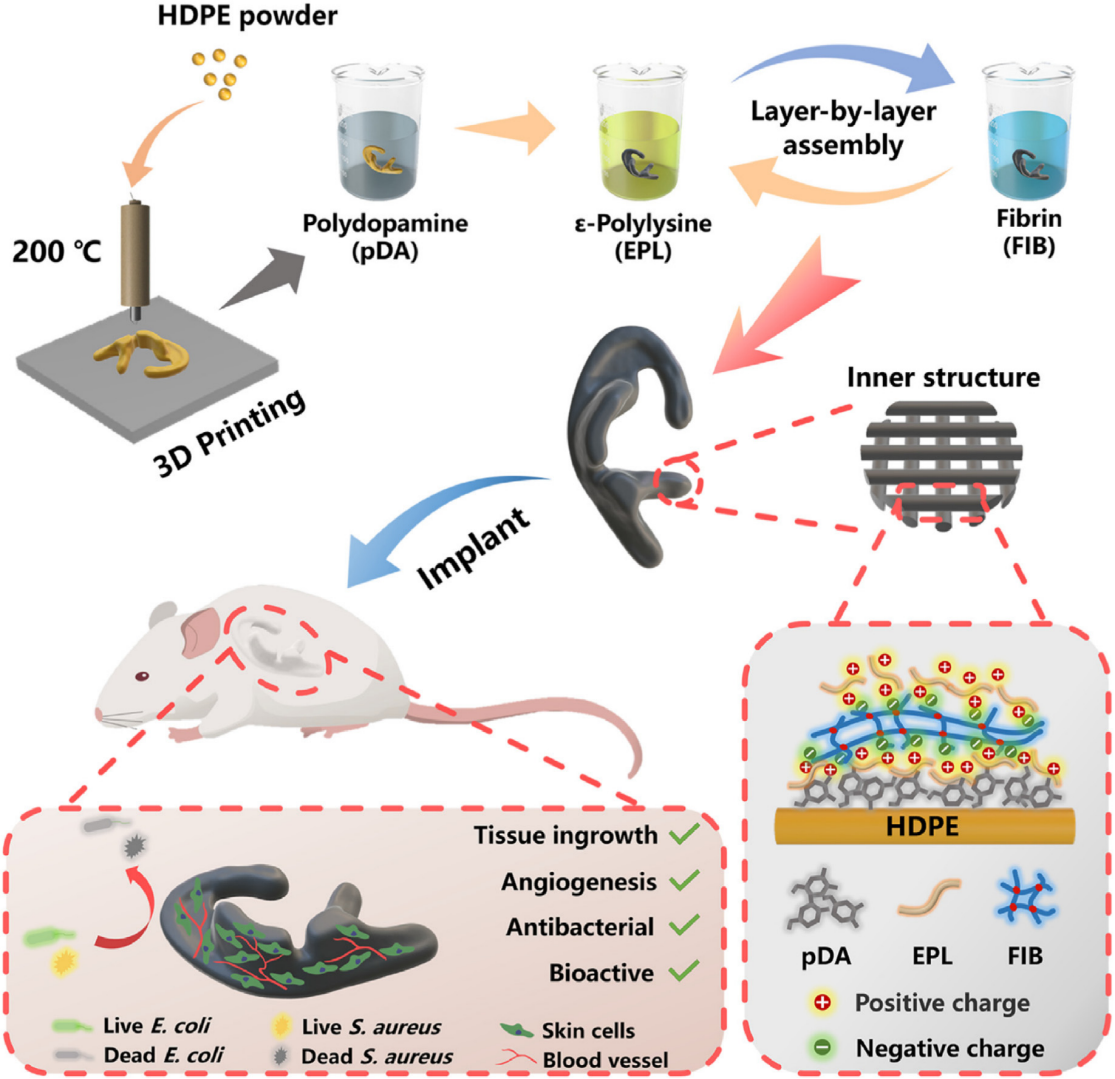
Preparation scheme of the multifunctional auricle scaffold by 3D printing and subsequent activation by polydopamine (pDA) and coated layer-by-layer with EPL and FIB. The pDA-EFE auricle scaffold obtained showed bioactive, antibacterial, angiogenesi enhancing, and tissue ingrowth-promoting properties. Reprinted with permission from Ref. [378]. Copyright 2022 Elsevier.

3D-printed high-density polyethylene (HDPE) scaffolds with bioactive and antibacterial layer-by-layer (LBL) modification have recently been developed for auricle reconstruction [378] (Fig. 15).

The polydopamine (pDA) coating method was used to construct a multilayer ε-polylysine and fibrin (FIB) modification on the surface of the 3D HDPE scaffold *via* the LBL self-assembly approach. The LBL strategy enhanced bioactive and antibacterial properties against Gram-positive *S. aureus* and Gram-negative *E. coli*.

### 3.6 Antifungal scaffolds

As the incidence of infections caused by invasive fungal pathogens has increased dramatically in the last twenty years, the fabrication of new antifungal scaffolds with antimicrobial properties is becoming more important [381,382]. Table 5 shows the antifungal scaffolds developed so far for tissue engineering applications.

**Table 5:** Antifungal scaffolds.

Material	Fabrication method	Application	Fungi	Non-toxicity: cell line/animal model	Year	Ref
Scaffolds with antibiotics						
Polyene gelatin fiber mats with antibiotics	Electrospinning	Skin	*Wide range of pathogenic yeasts and fungi*	Human corneal fibroblasts and human sclera fibroblasts	2014	[383]
Amphotericin-B and vancomycin-loaded CS nanofiber	Electrospinning	Skin	*C. albicans*	Not studied	2019	[384]
Recombinant spider silk proteins, silica NPs, antibiotics (gentamicin, AMB, …)	Casting, 3D printing	Tissue engineering	*P. pastoris yeast*	BALB/3T3 fibroblast cells	2020	[360]
PHA, nystatin and AMB	Solvent casting	Tissue engineering	*C. albicans, C. parapsilosis, filamentous fungi*	Not studied	2021	[385]
Silk sericin/PVA hydrogel loaded with azithromycin	A freeze/thaw process	Skin	*C. albicans*	NIH-3T3 fibroblasts and HaCaT ​cell lines	2022	[386]
**Scaffolds with metals**						
Ag-Loaded Cellulosic Fiber	Commercial fibers specified	Skin	*C. albicans, C. tropicalis and C. krusei*	Not studied	2006	[387]
PU membranes modified by zinc oxide nanoparticles	Precipitation and drying	Tissue engineering	*Aspergillus brasiliensis*	Not studied	2012	[388]
Ag:HA/Ti and Ag:HAp/TiO2 nanotubes	Pulsed laser deposition	Bone	*C. albicans and A. niger*	HEp2 human epidermoid carcinoma cells	2014	[389]
Genipin-crosslinked Gelatin/Nano Ag scaffolds	Lyophilization technique	Tissue engineering	*C. albicans*	MSCs	2014	[390]
Borophosphate glasses with antimicrobial oxides	Melt quenching technique	Tissue engineering	*C. albicans and F. solani*	Not studied	2018	[391]
PCL titanium dioxide and cefuroxime scaffolds	Electrospinning	Cornea	*C. albicans*	Human limbal stem cells	2020	[392]
Chitosan-AgNPs	Colloid	Skin	*C- albicans and other Candida species*	NIH/3T3 fibroblast cells	2021	[393]
**Scaffolds with antifungal polymers/peptides**						
*Cm*-p1 encapsulated nanofibers	Electrospinning	Skin	*C. albicans*	HUVEC human endothelial cells	2015	[394]
Halomonas-levan hydrogels	Crosslinking	Skin	*C. albicans*	L929 mouse fibroblast cells.	2020	[383]
**Scaffolds produced by combined strategies and alternative antifungal compounds**						
PCL-clotrimazole fibers	Melt co-extrusion process	Skin	*A. fumigatus, C. albicans, and T. mentagrophytes*	Mice	2017	[395]
Polymethacrylate polymer matrix, graphene and tolnaftate	Electrospinning	Skin	*T. rubrum and M. canis*	Not studied	2018	[396]
PU/PVP/SF nanofibers with sertaconazole nitrate	Electrospinning	Skin	*C. albicans*	3T3 fibroblast cells	2020	[397]
Gellan/PVA and eucalyptol, β-cyclodextrin	Electrospinning	Coating	*C. albicans and C. glabrata*	Not studied	2021	[398]
10-undecenoic acid based polyurethane/PCL fibers	Electrospinning	Skin	*C. albicans ​and ​C. tropicalis*	Not studied	2022	[399]

Incorporating bioactive materials into biodegradable polymers can provide drug-releasing bioactive scaffolds for potential use in novel controlled drug delivery, wound dressing, tissue engineering, stem cell regeneration and differentiation [400]. *In vitro* efficacy and toxicity of three classes of US Food and Drug Administration-approved antifungal-loaded fiber mats produced by electrospinning have been compared [401]. New chitosan-based mucoadhesive nanofiber mats were simultaneously loaded with VAN and Amphotericin B (AMB) as antibacterial and antifungal agents for the treatment of oral aphthous ulceration [384]. Films and scaffolds based on recombinant spider silk proteins with silica NPs (see antibacterial scaffolds in Section 3.4) were also loaded with the antibiotic and antimycotic AMB (in addition to specific antibiotics) to assess their antimycotic potential [360]. The derived composite materials showed good antimicrobial properties against the *E. coli* bacteria and *P. pastoris* yeast. AMB was also incorporated into PHA matrices combined with the antifungal agent nystatin at different concentrations [385]. The formulations, tested against different pathogenic fungi (*C. albicans* species and *C. parapsilosis*) as well as filamentous fungi, demonstrated a robust antifungal effect. The antifungal PHA composite inhibited the formation of *C. albicant* biofilm, although it was not efficient in the eradication of mature biofilms. Very recently, silk sericin/PVA hydrogel loaded with azithromycin was synthesized using a freeze/thaw process [386]. The hydrogel exhibited antimicrobial activity against *S. aureus*, ​*P. aeruginosa*, ​*E. coli*, and ​*C. albicans* and excellent cytocompatibility. accelerating the healing of infected burns while reducing systemic burn effects.

Metals (particularly Ag) and antimicrobial oxides have also been studied as antifungal agents in recent years. Hipler et al. prepared several textile commercial fibers (Sea-Cell fibers) from brown, red, green and blue algae loaded with Ag ions for potential application as antifungal and antibacterial textiles for skin conditions [387]. The fibers demonstrated antifungal activity against three Candida species and antibacterial activity against *S. aureus* and *E. coli* in a dose-dependent manner*.* A potential treatment for superficial candidiasis proposed the use of nanocomposites based on AgNPs in combination with CS. Inhibitory effects against several Candida species were found for concentrations between 0.06 and 1 ​μg/mL. When the antifungal fluconazole and the antibiotic and antifungal AMB were combined with the AgNPs, the composite showed an additive antifungal effect. The AgNPs/CS composites exhibited low cytotoxicity in mammalian cells [387].

Oxide metals are widely used compounds in scaffolds due to their broad-spectrum antimicrobial capacity [402]. PU membranes modified by nano-ZnO have exhibited important antifungal activity and can be successfully employed in biomedicine [388]. In another study, Trcin et al. prepared antimicrobial PCL/titanium dioxide (TiO_2_) and PCL/cefuroxime scaffolds by electrospinning. The scaffolds supported cell growth and differentiation of limbal stem cells and provided antimicrobial activity, particularly against the bacteria *P. aeruginosa*, *S. aureus* and the fungi *P. albicans*. These fiber mats would be suitable for the management of superficial fungal infections of the cornea and corneal tissue regeneration [392]. Borophosphate glasses doped individually with a few antimicrobial oxides such as CeO_2_, ZnO and CuO were prepared by the melt quenching technique [391]. The results revealed antimicrobial activity against some fungi and bacteria, that the addition of the antimicrobial oxides had a positive effect on the glass bioactivity and could play a part in biomedical applications [391]. In another study, the deposition of Ag/HAp thin films on Ti modified with TiO_2_ nanotubes substrates, followed by a heat treatment at 500 ​°C in water vapor for 6 ​h, produced efficient antifungal shield barriers for treating bone defects [389]. Yazdimamaghani et al. developed hybrid scaffolds consisting of gelatin and AgNPs produced by a green method and investigated their antimicrobial properties against Gram-positive *S. aureus*, Gram-negative *E. coli* and *C. albicans* [390]. The scaffolds interfered with the virulence factor of the *C. albicans* for invasion into the tissue and prevented hyphae-formation. The *in vitro* hMSC cell culture study on the samples revealed appropriate cytocompatibility [390].

Polymers with antimicrobial behavior in combination with antimicrobial agents have also been reported. Dermirci et at. developed hydrogels from Halomonas levan polysaccharide that possess antimicrobial activity and are loaded with AMB as antifungal agent with different crosslinking densities. The hydrogels showed good cytocompatibility with mouse fibroblasts in addition to exhibiting high antifungal activity against *C. albicans* due to the release of AMB [383].

Another strategy consists of producing scaffolds with antimicrobial peptides as an alternative approach for fungal control [403]. Antifungal *Cm*-p1 (Cencritchis muricatus peptide 1) was electrospun into a nanofiber scaffold for drug delivery to reduce the growth of *C. albicans* [394].

Other strategies have been developed based on antifungal agents not included in the previous categories or employing combined strategies. Thus, new PCL-based fibers useful for wound dressing were produced by the melt co-extrusion process with the clotrimazole antifungal and showed high antifungal capacity for 3 weeks, which was more than the same scaffolds manufactured by electrospinning [395]. Silk fibroin/PU/polyvinylpyrrolidone (SF/PU/PVP) nanofibers were prepared by electrospinning with the incorporation of the antifungal drug sertaconazole nitrate. The resulting nanofiber material exhibited fungicide activity against *C. albicans* from both silk fibroin (SF) incorporated into the PU/PVP nanofibers and PU/PVP nanofibers coated with SF as well as good biocompatibility. SF as an efficient polymer to sustain or control the release of antifungal agents can be considered a potential topical drug delivery system for the treatment of fungal infections as a topically applied scaffold [397]. Essential oils have also been proposed as antimicrobial agents. Mishra et. Al proposed a gelan/PVA nanofiber mat loaded with eucalyptol/β-cyclodextrin with antifungal capacity against *C. glabrata* and *C. albicans* that inhibited biofilm formation by 70% [398].

Finally, it is worth noting the development of combined strategies based on antifungal drugs and carbon nanomaterials. Misra et al. proposed a new approach combining the antifungal drug Tolnaftate (Tf) and graphene nanoplatelets, with polymethacrylate as a polymeric matrix, to prepare nanofibrous scaffolds for the treatment of topical infections [396]. They found superior antifungal activity of the Tf-graphene-loaded nanofibers as compared to Tf-nanofibers without graphene, demonstrating the efficacy of this strategy. 10-undecenoic acid based polyurethane/PCL fibers have recently been proposed as wound dressing materials to treat fungal diseases [399]. These scaffolds showed antifungal activity against *C. albicans* and *C. tropicalis*.

### 3.7 Antibiofilm scaffolds

Biofilms are the principal source of persistent infection and can become a serious problem in medical devices [404] because they protect microorganisms against drugs [405]. New antibiofilm scaffolds based on different strategies have been developed to solve this problem.

A novel three-dimensional scaffold containing multiple antibiotics (rifampin, levofloxacin, and vancomycin) has been designed to treat bone infections by rapid prototyping of a mixture of nanocomposite bioceramic and PVA with a coating of gelatin glutaraldehyde [123]. These antibacterial 3D scaffolds rapidly release rifampin, followed by the sustained and prolonged release of vancomycin and levofloxacin to destroy bacterial biofilms and inhibit bacteria growth in very short periods.

Antimicrobial metal Ag has also been reported as a tool to prevent or destroy microbial biofilms. PVA-Ag and CS-Ag nanocomposites obtained from AgNPs mixed with PVA or CS showed higher thermal stability than pure PVA and CS and enhanced AgNP antimicrobial and antibiofilm activities, which resulted in the efficient eradication of bacterial and biofilm growth of multi-drug resistant clinical isolates [364]. The presence of antimicrobial Ag showed significantly low cytotoxicity against liver cells. In another study, impregnated silver nanoparticles on radiosterilized pig skin nanocomposites inhibited bacterial growth and prevented biofilm while allowing non-cytotoxicity in mesenchymal stem cell culture at low AgNPs concentrations [282]. MRSA is considered a common colonizer of burn wounds and accounts for high morbidity and mortality rates all over the world [406]. Two formulations containing moxifloxacin for topical delivery were prepared and confirmed their efficacy in an MRSA-infected burn wound in BALB/c mice [407]. *In vivo* studies showed that the two gels have the same efficacy in eradicating bacteria from the wound site when treatment was started during the early stages of infection. On the other hand, in a delayed treatment, a new gel was more efficient than a traditional gel in burn wounds infected with *S. aureus*, both planktonic and biofilm [407]. Colonization of the lungs by biofilm-forming pathogenic microorganisms is a major cause of mortality in cystic fibrosis (CF) due to the difficulty of dealing with the biofilm exopolysaccharide matrix produced by the pathogens and the viscous mucus [408]. The use of alginate in combination with NO has shown improved activity compared to common antibiotics for chronic CF infections [372].

Another strategy to fight bacteria and achieve osteo proliferation is to use palladium nanoparticles (PdNPs) that are anchored to polypyrrole-functionalized rGO nanocomposite (Pd/βy/rGO NC). These PdNPs were able to prevent the bacterial biofilm formation caused by common human pathogens such as *P. aeruginosa, K. pneumoniae, B. subtilis* and *E. coli* [375]. Proper wound healing is often affected by bacterial infection. Murugesan et al. prepared a nanocomposite that combined another carbon nanomaterial, multi-walled carbon nanotubes (MWCNT) modified with a polypyrrole (PPy) matrix with the incorporation of PdNPs [371]. This MWCNT/PPy/Pd hybrid composite prevented the formation of biofilms *in B. subtilis, P. aeruginosa, E. coli* and *K. pneumoniae* bacteria and showed a significant dose-dependent toxicity in Vero and HeLa cells.

Adeli-Sardou et al. reported that lawsone incorporated into PCL/gelatin nanofibers *via* electrospinning exhibited potential antibacterial and antibiofilm activity [313]. All lawsone-containing scaffolds showed antibacterial activity against *S. aureus* and MRSA and PCL/Ge/lawsone 10% prevented the growth of *P. mirabilis*. In conclusion, significant anti-biofilm activity was observed in all biofilm strains [313].

Bioactive glasses (BGs) have been proposed as promising materials for the reconstruction of periodontal and peri-implant bone defects due to their favorable structural and antimicrobial properties [409]. Porous novel complex drug carrier porous nano-HAp/CS/konjac glucomannan scaffolds were combined with liposomes containing vancomycin to provide sustained release and impede biofilm formation [206].

## 4 Antimicrobial characterization of scaffold materials

**Fig. 16:**
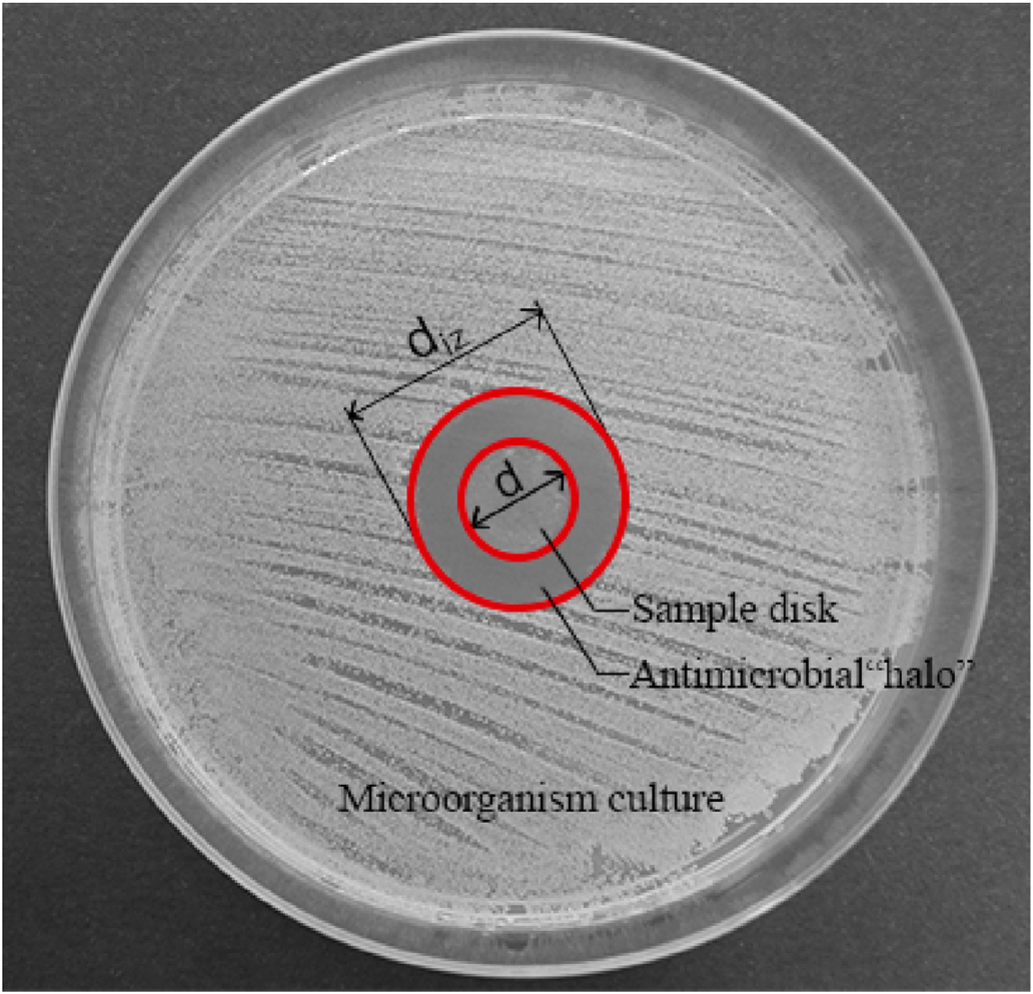
Normalized width of the antimicrobial “halo” of a scaffold calculated by the inhibition zone (d_iz_) and the scaffold diameter (d). Reprinted by kind permission of ref. [410]. Copyright 2018 MyJoVE Corporation.

The agar disk diffusion test is one of the most useful antimicrobial tests in the field of biomaterials, which is based on incubating the bacteria on a plate in the presence of a disk of the scaffold material [410]. This test is recommended for a broad range of microorganisms such as Gram-positive bacteria (e.g., *S. aureus*), Gram-negative bacteria (e.g., *E. coli*) and yeast (e.g. *Candida albicans*). If the material shows antimicrobial activity, an inhibition halo forms around it where the bacteria will not be able to grow. The antimicrobial activity of the scaffolds can be analyze by measuring the diameter of the halo and comparing it with the positive and the negative control [37,410] (Fig. 16).

The normalized halo must be determined by applying Equation (1) to compare the antimicrobial activity of several scaffold materials [410].

(1) $$mw_{halo}=\frac{\frac{d_{iz}-d}{2}}{d}$$

The antimicrobial action of the materials tested for the growth of microorganisms is expressed by the normalized width of the antimicrobial “halo” (*nw*_*halo*_), determined by the inhibition zone diameter (*d*_*iz*_) and scaffold disk diameter (*d*). The diameters can be measured by image analysis software (e.g. the recently developed Image J or Antibiogramj free open source software [411]) from a photograph of the microbial culture. To ensure reproducibility, each antimicrobial test is carried out at least three times in quadruplicate on different days. This test is similar to the antibiogram test [412], in which disks impregnated with different antibiotics or the same antibiotic in different concentrations are used to test their antibacterial capacity.

Another method commonly used to characterize scaffold antimicrobial properties is based on the ISO standard 22,196:2007 to measure the antimicrobial activity on material surfaces (contact method). In this method, the microorganisms are placed directly on the scaffold and their growth inhibition can be determined by the colony counting procedure after a certain amount of contact time [410].

**Fig. 17:**
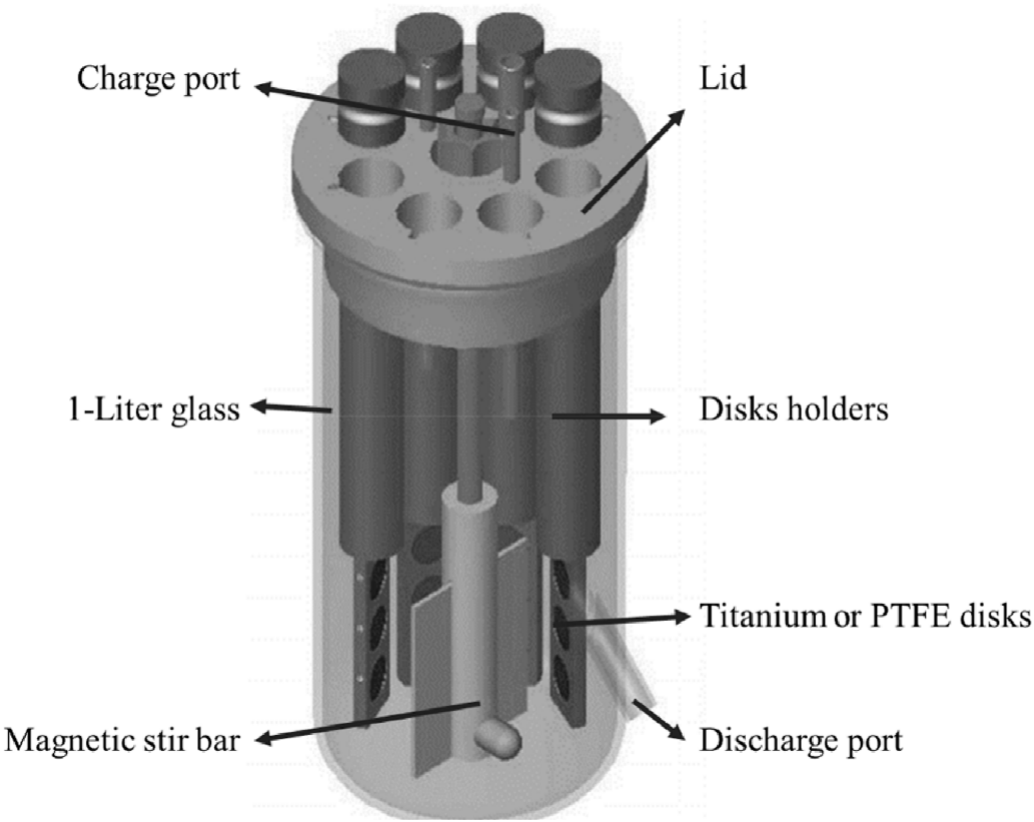
Schematic representation of a CDC Biofilm Reactor used to study biofilm formation on scaffold prepared with in the form of disks. Bioreactor fabricated by BioSurface Technologies Corporation (http://biofilms.biz/). Reprinted with permission under a Creative Commons CC BY 4.0 License from Ref. [416]. Copyright 2020 MDPI.

However, as bacteria and fungi can resist antimicrobials by forming biofilms [413], the study of biofilm formation on scaffolds is an important issue in tissue engineering. The capacity of a scaffold to impede biofilm formation can be studied by putting it in contact with a bacterial culture on well culture plates [414] or in bioreactors [415] (Fig. 17).

The presence of bacterial biofilm can also be analyzed by SEM [37,414], confocal microscopy [417,418] and/or atomic force microscopy [419,420].

The antimicrobial capacity of a material can also be measured by putting it in contact with a bacterial suspension in its culture medium and measuring optical density at different times [209]. Rising absorbance indicates increased bacterial activity.

## 5 Antimicrobial mechanisms

Microbial infections on an implanted scaffold surface can eventually lead to biofilm formation and thus impede the use of the scaffolds in humans. Despite an effective host immune system, the scaffold surface can be rapidly occupied by microorganisms, resulting in persistent infection, implanted scaffold failure and can even cause the patient's death [421]. These problems are difficult to solve because microorganisms such as bacteria and fungi possess complex mechanisms to adhere to scaffolds that vary according to the microbial strain. Several antimicrobial scaffolds have been developed by incorporating antibiotics in the scaffold material matrix. However, as bacterial resistance is increasing at an alarming rate [13], this strategy will probably not provide long-lasting solutions to tissue engineering. In this regard, other antimicrobial strategies consisted of scaffolds capable of releasing other types of antimicrobial agents such as antiseptics, antimicrobial polymers, peptides, metals, carbon nanomaterials and combinatorial strategies. Another strategy consists of developing scaffolds made of smart materials, i.e. stimuli-responsive biomaterials, such as toxin-triggered, pH-responsive or dual stimulus-responsive adaptive antimicrobial materials [[422], [423], [424]]. Biofilm can also be combated by modifying the scaffold surface by diverse strategies to produce an antifouling (superhydrophobic, non-charged or highly hydrated) surface that prevents the bacteria adhering to the implant or a bactericidal surface that kills the bacteria in contact with the implants [425,426]. The mechanisms of bacterial adhesion, biofilm formation and the released substances are discussed in detail in Ref. [421].

**Fig. 18:**
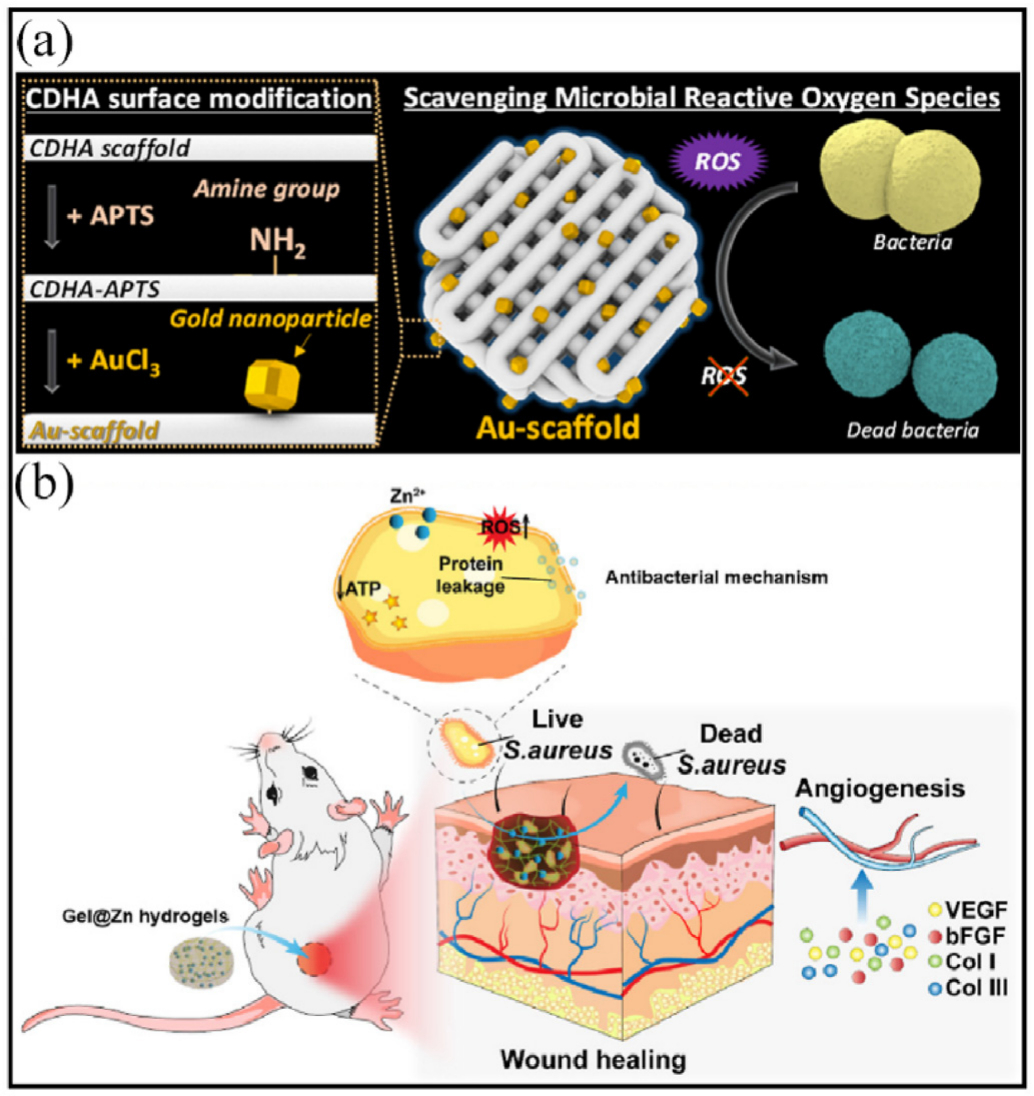
Schematic illustration of antimicrobial mechanism in: (a) 3D-printed biocompatible scaffolds based on calcium-deficient hydroxyapatite (CDHA) with gold nanoparticles. Reprinted with permission from Ref. [204]. Copyright 2019 Elsevier; (b) gelatin-based and Zn2+-incorporated composite hydrogel (Gel@Zn) for bacterial elimination to promote infected wound healing. Reprinted with permission from Ref. [292]. Copyright 2022 Elsevier.

The exact antimicrobial mechanism of nanoparticles and nanomaterials is not yet clearly understood, but it may be attributed to the production of reactive oxygen species (ROS) that damage the cell membrane [425]. It is well-known that when the production of excessive ROS exceeds the bacteria's scavenging ability it will cause fatal damage to the microorganism [229]. 2D MoS_2_ exhibits broad antibacterial activity associated with the production of ROS in polyhydroxyalkanoate/chitosan (PHA/CS) and 2D molybdenum disulfide–doped (2D MoS_2_) scaffolds [114]. Scaffolds with TiO_2_ also possess effective antibacterial activity because TiO_2_ damages the bacteria by generating ROS and destroying their structure and functions [180]. The bactericidal effect of metals such as silver or gold is also attributed to their ROS-scavenging properties [427]. 3D-printed biocompatible scaffolds based on calcium-deficient hydroxyapatite (CDHA) with gold nanoparticles were able to produceROS effective against *Micrococcus luteus* (Fig. 18 (a)) [204]**.**

The potential antibacterial mechanism of Zn-embedded biomaterials is also mainly related to the production of ROS [428]. For example, the antibacterial mechanism of a gelatin-based and Zn^2+^-incorporated composite hydrogel (Gel@Zn) for rapid infected wound healing consisted of reducing the ATP level, generating ROS and leakage of protein [292] (Fig. 18(b)). PLGA/Cu(I)@ZIF-8 scaffolds produced by combining antibacterial copper-loaded-zeolitic-imidazolate-frameworks (ZIF-8) and PLGA [194] generate ROS in the presence of H_2_O_2_, which contributes to their superior antibacterial activity *in vitro* and *in vivo*. PGA-based scaffolds produced by cation exchange of MMT with Cu^+2^ and the introduction of CTAB into the MMT interlayer showed strong antibacterial activity due to the high level of ROS release [226].

The study of the antibacterial mechanism of a PCLA scaffold with nano-hydroxyapatite coating doped green tea epigallocatechin-3-gallate against MRSA showed that the wall and membrane structure of the bacteria were seriously damaged [229] so that the intracellular components such as nucleic acid and proteins flowed out of the cell. It was also determined that the scaffold promoted the production of ROS in MRSA cells, which attack important macromolecules in bacterial cells (e.g., nucleic acid, proteins and lipids) and eventually cause cell death.

The antibacterial activity of xyloglucan-co-methacrylic acid/hydroxyapatite/SiO_2_ nanocomposite scaffolds was shown to be related to the penetration of silica and hydroxyapatite nano-particles into bacteria to interact with the cellular protein [222].

The intrinsic antimicrobial activity of dopamine is due to its ability to effectively eradicate bacteria, microbes, plankton, or biofilm and it has been used to produce antimicrobial scaffolds [429,430]. Small molecules of catechol and proteins of amine in the presence of alkaline condition (aqueous) and oxygen allow dopamine to undergo polymerization to form a thin adherent PDA film, which prevents the attachment and growth of bacteria by taking over the bacteria's nutrient supply [431]. In most cases, this antibacterial activity has been attributed to the benzene ring of the dopamine molecule and the formation of local toxic effects by active groups formed on the outer membrane of the bacteria cell, which affect the permeability of the cell membrane by obstructing the components required for the bacteria to survive.

The antimicrobial mode of action of quaternary ammonium compounds against pathogens is attributed to positively charged nitrogen atoms [18], which eradicate microorganisms by damaging their membranes. The antimicrobial mechanism of antimicrobial polymers such as chitosan is also attributed to their positively-charged linear structure [432].

Carbon nanomaterials prevent the formation of microbial biofilms mainly through preventing microbial adhesion by reducing surface free energy and increasing hydrophobicity and killing them mostly by oxidative stress and photothermal/photodynamic effects [433]. The release of AgNPs from the Ag-GO nanohybrids introduced into PLA promoted the generation of bacteria-inhibiting ROS [227].

Nonetheless, there are still many questions to answer regarding the antimicrobial mechanism of action of many of the scaffolds included in this review and further research is required to obtain a complete understanding of all these antimicrobial processes.

## 6 Toxicological aspects

Scaffolds with antibacterial and/or antifungal properties must not be toxic to human beings and should be subjected to toxicological assays to ensure their safe use in tissue engineering. The different approaches to developing antimicrobial scaffolds use antibacterial and antifungal agents that can be toxic at certain concentrations, so that it is crucial to find a balance between the scaffolds’ biocidal properties and cell biocompatibility. For example, the cell viability of PCL-AgNPs prepared by electrospinning revealed that cytotoxicity was highly dependent on the concentration of AgNPs [115].

Biocompatibility was considered and tested by *in vitro or in vivo* tests in most of the studies in this review. The cytotoxicity of antibacterial bone regeneration scaffolds was assessed using different cell lines such as osteoblasts, preosteoblasts, MSCs, osteosarcoma cells and fibroblasts (Table 1). In scaffolds that contain antibiotics, the biocompatibility of those loaded with levofloxacin hydrochloride was studied in a fibroblast cell line [127]. Toxicity assays performed with other cell lines like MSCs have been carried out on scaffolds loaded with CPFX [122], vancomycin [125,126,130], and minocycline [37]. MC3T3-E1 preosteoblasts were used to analyze bioceramic-PVA scaffolds loaded with several antibiotics (rifampin, levofloxacin and vancomycin) [123] and scaffolds containing VEGF and cephalexin [128]. MG-63 osteoblast cells were also used as cell lines in scaffolds containing vancomycin. Cytotoxicity assays with scaffolds loaded with strychnine were performed on the human osteoblast cell line hFOB1.19 [129]. However, the toxicological aspects of scaffolds developed with gentamicin [121] and TCH [235] have not yet been studied.

The biocompatibility of scaffolds prepared with antibacterial polymers or those that include peptides as antibacterial agent has been evaluated in MC3T3-E1murine preosteoblast [135,[137], [138], [139], [140]] and ET3 mouse fibroblasts [136]. Human osteoblasts [132] and MSCs [133,141] from rabbits have also been used in some studies. In the study performed by Li et al. in which HACC-grafted PLGA/HAp scaffolds were prepared by FDM 3D printing, cytotoxicity and *in vivo* performance were assessed by rabbit MSCs and New Zealand white rabbits, respectively [63]. Cytotoxicity assays on scaffolds that incorporate carbon nanomaterials have been performed using MC3T3-E1 preosteoblast cells [148,149], human osteoblasts [147], human MSCs [145], osteosarcoma cells [144] and fibroblasts from human [146] and murine [143] origin. In addition, antibacterial scaffolds with antibacterial metals have been assessed in several cell lines to prove their biocompatibility. In several studies, murine preosteoblasts MC3T3-E1 [162,165,179,181,187,188,190,192,195,203], MSCs [157,159,160,172,176,177,189,191,194], osteoblastic cell line [155,178,182,202], osteoblast-like cells [156,158,161,166,167,173,180,183,198], fibroblasts [184,196], and breast cancer cells [186] were used as models. *In vitro* bioactivity in an acellular simulated body fluid (SBF) was examined in some studies [154,169,170] and also metal ion penetration with bovine bone specimens [169]. Other studies also reported *in vivo* assessment of biocompatibility in rat [194] or rabbit [168,203] models. Finally, in scaffolds developed with combined or alternative strategies, biocompatibility was analyzed in MC3T3-E1 preosteoblasts [216,223], osteoblasts [208,212,221] and osteoblast-like cells [210,213,214,217,220,223,225], MSCs [176,207,209,211,215,224], although it was not assessed in other studies [206,219,226]. *In vivo* assays performed on rat [208] and rabbit models [209] were also reported.

The toxicity of antibacterial scaffolds developed for skin tissue engineering has been assessed in a wide variety of cells, such as fibroblasts, melanocytes, and keratinocytes (Table 2). Nanofibrilar scaffolds loaded with the antibiotic CPFX (concentration up to 0.025 ​g/mL) prepared by jet-spraying were assessed in dermal fibroblasts [273]. Cell colonization was complete after 12 days, suggesting that cells were proliferating within the nanofibers with no evident cytotoxicity. In other studies on antibiotic-loaded scaffolds, cytotoxicity was not analyzed [272,274,275]. Biocompatibility in scaffolds loaded with antibacterial metals was assessed in different cell lines. AgNPs or Ag ions incorporated into the polymer matrix were evaluated in fibroblast cells [280,283,287], MSCs [282] and human melanocytes [290]. Ai et al. prepared swellable hemostatic scaffolds by 3D printing, which incorporated AgNPs [281]. Besides a broad-spectrum antibacterial effect, the system also demonstrated good biocompatibility with several cell lines (A549 adenocarcinomic human alveolar basal epithelial cells, U251 fibroblast-like cells, and epithelial-like cells (HepG2 and HBE cell lines). The *in vivo assay* in a rabbit femoral vascular injury model also indicated a rapid hemostatic effect. The cytotoxicity of oxygen-generating nanofiber with calcium peroxide as an antibacterial agent was assessed in human osteoblasts [278], and BMCSs [286] were used as models to evaluate biocompatibility. Both studies found the scaffolds to have good biocompatibility. *In vitro,* the cytocompatibility of bioactive glass nanocomposite hydrogels containing Cu ions was analyzed with endothelial progenitor cells, and a rat model was used in the *in vivo* assay [289]. The hydrogels significantly promoted cell viability, proliferation and angiogenic ability, while accelerating wound healing and skin tissue regeneration in a diabetic wound. Finally, the biocompatibility of hydrogels containing silk fibroin and zinc chromide NPs as antibacterial agent was analyzed in Hu02 fibroblast and a mouse model to assess their efficacy in wound healing [291]. After five days, the wounds of mice treated with the nanocomposite scaffold were almost completely healed. Some studies, however, did not include biocompatibility assays [279,284,285,288]. Fibroblasts [293,294,299,300,302,304], fibroblast-like cells [301], epidermal [296], endothermal [297] and keratinocytes [298,303] were used as cell models to evaluate the cytotoxicity of antibacterial scaffolds prepared with antibacterial polymers, peptides and scaffolds incorporating carbon nanomaterials. The approaches reported by the *in vivo* assays also showed good wound healing efficacy [293,299,302,303]. Finally, biocompatibility of antibacterial scaffolds prepared by combining strategies and alternative methods has been evaluated using fibroblasts (both human and murine) [[307], [308], [309],^312^,^314^,^320^], fibroblast-like cells [316,321,322], epithelial cells [306], osteoblasts [312], and also Schwann cells [318] and red blood cells [322]. *In vivo* experiments showed the effectiveness of the technique in wound healing in the mouse model [307,316], absorbable sutures in a rabbit model [318] and a bacterial infection model performed on rats [322]. Nevertheless, some studies did not report on biocompatibility assessment [41,310,311,313,315,317,319,323].

The toxicity of antibacterial scaffolds developed for oral tissue regeneration (Table 3) has been studied mostly in hDPCs as a model. This cell line has been used in different strategies that involve the incorporation of antibiotics [340,341] and other antibacterial agents [350,351]. Gingival cells [40,347] and murine preosteoblasts MC3T3-E1 [344,345] have also been used as cell models to assess biocompatibility in antibacterial scaffolds for oral tissue regeneration. *In vivo* assessment was performed in bioglass/CS/chlorhexidine gluconate scaffolds developed for dental application, in which osteoinductive ability was proven using a Wistar-Furth rat model [348]. Conversely, toxicology assays were not included in several other studies [339,342,343,346].

Toxicological assays were performed on antibacterial scaffolds for muscle, nerve, trachea and other tissue engineering applications for oral regeneration (Table 4). Myoblast (C2C12 and L6 cell lines) were used as cell models to assess the biocompatibility of scaffolds for muscle regeneration using different strategies such as antibacterial metals incorporated in polymeric matrices [353,365] or essential oils as antibacterial agents [377]. Scaffolds based on chitosan-aniline tetramer showed good biocompatibility in C2C12 myoblasts and ADMSCs [368]. The biocompatibility of cardiac regeneration scaffolds that incorporated the PANI antibacterial polymer was shown using 3T3 fibroblast-like cell lines [373], while antibacterial scaffolds that incorporated rGO as antibacterial nanomaterial were proven with HUVEC [374]. The toxicity of antibacterial fibrous membranes based on PLA/GO/IL for trachea regeneration was assessed using L929 fibroblast cells, while they confirmed the favorable biocompatibility and promotion of tissue regeneration in a rabbit model. Antibacterial scaffolds for non-specific tissue-engineered applications were assessed using different cell lines such as MSCs [115,354,363], fibroblasts [118,366,367], fibroblast-like cells [359,360], osteoblasts [370], osteoblast-like cells [375], human osteosarcoma cell line [371], liver cells [364] or keratinocytes [114,117], although biocompatibility was not assessed in several studies [116,349,358,361,362,369,372,376].

The cytotoxicity studies carried out on antifungal scaffolds for tissue engineering are included in Table 5. Scaffolds for skin regeneration that include antifungal properties based on the incorporation of antibiotics, metals, peptides and also the use of antimicrobial polymers have been assessed using mainly human [383] or murine fibroblasts [383,393,397]. Biocompatibility studies of scaffolds based on PLC/TiO_2_/cefuroxime for cornea regeneration were performed with human limbal stem cells [392], which showed good behavior regarding cell adhesion, proliferation, and differentiation. Mofidfar et al. prepared polymeric nanofibers of PCL containing the antifungal compound clotrimazole by co-extrusion with poly (ethylene oxide), which was subsequently removed. The scaffold showed effective antifungal behavior in an *in vivo study* (mouse model) as well as good biocompatibility [395], indicating a good potential for wound healing. Cytotoxicity studies were carried out on general-purpose antifungal scaffolds for tissue engineering using several cell lines such as MSCs [390], fibroblasts [360,393], human endothelial cells, human carcinoma cells [389] and human endothelial cells [394], although biocompatibility assays were not reported in several studies [384,385,387,388,391,396,398].

## 7 Conclusions and future perspectives

A lot of progress has been made in the development of scaffolds with antimicrobial activity against bacteria and fungi for a broad range of tissue engineering applications, including bone, oral tissue, skin, muscle, nerve, trachea, cardiac and other applications. Scaffolds produced by different methods to provide antimicrobial activity are essential to avoid microbial infections, which can dramatically affect an implant's success. Antimicrobial activity against bacterial and fungal growth and biofilm formation can be achieved by combining scaffold materials with a broad range of antimicrobial agents such as antibiotics, antiseptics, antimicrobial polymers, peptides, metals, carbon nanomaterials and combined strategies. As multidrug-resistant infections are increasing at an alarming rate, alternative regenerative medical platforms are essential to ensure safe clinical treatments. This review has described the state of the art of antimicrobial scaffolds capable of impeding bacterial and fungal infections in tissue engineering. However, the antimicrobial mechanisms involved in these tissue engineering approaches capable of impeding infections and biofilm formation still need further investigation. The toxicological aspects of these antimicrobial scaffolds have been ensured in most of these studies for safe clinical transfer. There is now a broad range of antimicrobial characterization techniques available to study the antimicrobial behavior of a scaffold against bacterial and fungal growth and biofilm such as the agar disk diffusion test, contact method or biofilm formation in well culture plates or in bioreactor. A broad range of fabrication methods of antimicrobial scaffolds have been included in this review. The best method and materials for tissue engineering depend on the specific applications involved. The antimicrobial approaches now able to prevent infections, including those produced by multidrug-resistant strains, show great promise for future clinical tissue engineering applications.

## Author contributions

**Á.S.A**. conceived the idea for this work, wrote the draft manuscript, prepared the figures, performed major editing, reviewed and proofread the manuscript. **A.C–V**. and **R. SiS**, **M.E-T**, **A.A.A.A**, **M.M.T**. and **Y·K.M**. reviewed, edited and proofread the manuscript.

## Declaration of competing interest

The authors declare that they have no known competing financial interests or personal relationships that could have appeared to influence the work reported in this paper.

## Abbreviation list

**5FU**: Fluorouracil
**AB**: Airbrush
**ADMSCs**: Adipose-derived mesenchymal stem cells
**ADSF**: antibacterial degummed silk fibers
**Ag-MBGN**: bioactive glass nanoparticle modified with Ag
**AgNPs**: silver nanoparticles
**ALP**: Alkaline phosphatase activity
**AM**: Additive manufacturing
**AMB**: amphotericin B
**ARX**: Arabinoxylan
**BAMG**: bladder acellular matrix graft
**BCP**: Biphasic calcium phosphate
**BGs**: Bioactive glasses
**BMP-2**: Bone morphogenic protein-2
**BMSCs**: Bone marrow stromal cells
**CA**: Cellulose acetate
**CBNs**: Carbon nanomaterials
**CEF**: Cephalexin
**CF**: Cystic fibrosis
**cHApNPs**: citrate-hydroxyapatite nanoparticles
**CG**: Carrageenan
**Ch-hBN**: Boron nitride doped/chitosan
**CHX**: Chlorhexidine
**CMARX**: carboxymethylarabinoxylan
**CMC**: carboxy-methyl-cellulose
**COL**: Collagen
**CPFX**: Ciprofloxacin
**CPO**: Calcium peroxide
***Cm*-p1**: cencritchis muricatus peptide 1
**CS**: Chitosan
**CS-AT**: Chitosan-graft-aniline tetramer
**CTAB**: Cetyltrimethylammonium bromide
**Cu-BGs**: Bioactive glasses containing Cu^+2^
**DNA**: deoxyribonucleic acid
**ECM**: Extracellular matrix
**EGCG**: Epigallocatechin gallate
**EPL**: ε-poly-l-lysine
**FDA**: Food and Drug Administration
**FDM**: Fused deposition modeling
**FIB**: Fibrin
**Ge**: gelatin
**GCG**: genipin cross-linked gelatin
**GO**: Graphene oxide
**GQDs**: Graphene quantum dots
**HACC**: Hydroxypropyl trimethylammonium chloride chitosan
**HAp**: hydroxyapatite
**hDPCs**: Human dental pulp cells
**HDPE**: High-density polyethylene
**hFOB**: Human foetal osteoblastic cells
**HIF**: Hypoxia-inducible factor
**hMSCs**: human mesenchymal stem cells
**HNT**: Halloysite nanotubes
**HUVEC**: Human umbilical vein endothelial cells
**IL**: Ionic liquid
**LBL**: Layer-by-layer
**L-ECM**: Liver extracellular matrix
**L929**: mouse fibroblast cells
**MBG**: Mesoporous bioactive glass
**MC**: 1-chloro-2,2,5,5-tetramethyl-4-imidazolidinone
**MET**: Metronidazole
**MH**: Minocycline hydrochloride
**MMT**: Montmorillonite
**Mon-CPFX**: Monticellite-ciprofloxacin
**MRSA**: Methicillin-resistant *Staphylococcus Aureus*
**MSCs**: Mesenchymal stem cells
**MWCNT**: Multi-walled carbon nanotubes
**NASH**: nonalcoholic steatohepatitis
**nHP66**: nano HAp/polyamide 66
**NPs**: Nanoparticles
**PANI**: Polyaniline
**PCL**: Polycaprolactone
**pDA** or **PDA**: Polydopamine
**PDCs**: Polymer-derived ceramics
**PDS**: Polydioxanone
**PEC**: Polyelectrolyte complex
**PEEK**: Polyetheretherketone
**PEG**: Poly (ethylene glycol)
**PEG-DA**: dibenzaldehyde-terminated poly(ethylene glycol)
**PETA**: Pentaerythritol triacrylate-co-trimethylolpropane tris (3-mercaptopropionate)
**PDMAEMA**: Quaternized polystyrene-*b*-poly(dimethylaminoethyl methacrylate)
**PdNPs**: Palladium nanoparticles
**PGA**: Polyglycolic acid
**PGS**: Poly(clycerol sebacate)
**PHA**: Polyhydroxyalkanoate
**PHBV**: poly(3-hydroxybutyrate-*co*-3-hydroxyvalerate)
**PHEA**: Poly(2-hydroxyethyl acrylate)
**PHMB**: Polyhexamethylene biguanide
**PLA**: Polylactic acid
**PLGA**: Poly lactic-*co*-glycolic acid
**PMMA**: Poly (methyl methacrylate)
**PPβ**: β-tricalcium phosphate-doped-PLGA/PCL
**PPC**: Chlorhexidine-doped PLGA/PCL
**PPXG**: Poly (*p*-xylyleneguanidine) hydrochloride
**PPy**: Polypyrrole
**PS**: Polystyrene
**PU**: Polyurethane
**PVA**: Polyvinyl alcohol
**PVP**: Polyvinyl pyrrolidone
**PVS**: Polyvinyl-siloxane
**QCNS**: Quaternary chitin/partially deacetylated chitin nanofibers as their skeleton
**QCS**: Quaternized chitosan
**QOS**: Quaternary ammonium organosilane
**RGD**: Tripeptide Arg-Gly-Asp
**rGO**: Reduced graphene oxide
**rhBMP-2**: Recombinant human bone morphogenetic proteins
**ROS**: Reactive oxygen species
**RPS**: Radiosterilized pig skin
**RSF**: Regenerated silk fibroin
**SA**: Sodium alginate
**SBF**: Simulated body fluid
**SeNPs**: Selenium nanoparticles
**SF**: Silk fibroin
**SLS**: Selective laser sintering
**TAP**: Tripe antibiotic paste
**TCH**: Tetracycline hydrochloride
**TCP**: Tricalcium phosphate
**TEOS**: Tetraethyl orthosilicate
**Tf**: Tolnaftate
**TrGO**: Thermally reduced GO
**Ti6Al4V**: pTi
**UBM-ECM**: Urinary bladder extracellular matrix
**UHMWPE**: Ultrahigh-molecular-weight polyethylene
**VAN**: Vancomycin
**VEGF**: Vascular endothelial growth factor
**ZFHAp**: Zn and Fe integrated hydroxyapatite
**ZIF-8**: Zinc-based zeolitic-imidazolate-frameworks
**2D MoS**_**2**_: 2D molybdenum disulfide–doped
**γ-PGA**: γ-polyglutamic acid

## References

[1] Ambekar R.S., Kandasubramanian B. (2019). Progress in the advancement of porous biopolymer scaffold: tissue engineering application. Ind. Eng. Chem. Res..

[2] DE Boer J.B. (2022).

[3] Ratner B.D., Hoffman A.S., Schoen F.J., Lemons J.E. (2012).

[4] Ahmadian E., Dizaj S.M., Eftekhari A., Dalir E., Vahedi P., Hasanzadeh A., Samiei M. (2020). The potential applications of hyaluronic acid hydrogels in biomedicine. Drug Res..

[5] Moreno-Manzano V., Zaytseva-Zotova D., López-Mocholí E., Briz-Redón Á., Strand B.L., Serrano-Aroca Á. (2020). Injectable gel form of a decellularized bladder induces adipose-derived stem cell differentiation into smooth muscle cells in vitro. Int. J. Mol. Sci..

[6] Aparicio-Collado J.L., Novoa J.J., Molina-Mateo J., Torregrosa-Cabanilles C., Serrano-Aroca Á., Sabater I Serra R. (2021). Novel semi-interpenetrated polymer networks of poly(3-hydroxybutyrate-co-3-hydroxyvalerate)/poly (vinyl alcohol) with incorporated conductive polypyrrole nanoparticles. Polym.

[7] Rivera-Briso A.L., Aparicio-Collado J.L., Serra R.S.I., Serrano-Aroca Á. (2022). Graphene oxide versus carbon nanofibers in poly(3-hydroxybutyrate-co-3-hydroxyvalerate) films: degradation in simulated intestinal environments. Polymers.

[8] Rivera-Briso A.L., Serrano-Aroca Á. (2018). Poly(3-Hydroxybutyrate-co-3-Hydroxyvalerate): enhancement strategies for advanced applications. Polymers.

[9] Eftekhari A., Dizaj S.M., Sharifi S., Salatin S., Saadat Y.R., Vahed S.Z., Samiei M., Ardalan M., Rameshrad M., Ahmadian E., Cucchiarini M. (2020). The use of nanomaterials in tissue engineering for cartilage regeneration; current approaches and future perspectives. Int. J. Mol. Sci..

[10] Serrano-Aroca Á., Vera-Donoso C.D., Moreno-Manzano V. (2018). Bioengineering approaches for bladder regeneration. Int. J. Mol. Sci..

[11] Sill T.J., von Recum H.a. (2008). Electrospinning: applications in drug delivery and tissue engineering. Biomaterials.

[12] Kostarelos K., Prato M., Va E., Merino S., Martı C. (2015). Nanocomposite hydrogels: 3D polymer À nanoparticle synergies for on-demand drug delivery. ACS Nano.

[13] WHO (2018). http://www.who.int/mediacentre/news/releases/2018/antibiotic-resistance-found/en/.

[14] Pelgrift R.Y., Friedman A.J. (2013). Nanotechnology as a therapeutic tool to combat microbial resistance. Adv. Drug Deliv. Rev..

[15] Klinkajon W., Supaphol P. (2014). Novel copper (II) alginate hydrogels and their potential for use as anti-bacterial wound dressings. Biomed. Mater..

[16] Liu Y., Wang X., Yang F., Yang X. (2008). Excellent antimicrobial properties of mesoporous anatase TiO2 and Ag/TiO2 composite films. Microporous Mesoporous Mater..

[17] Jia Z., Shen D., Xu W. (2001). Synthesis and antibacterial activities of quaternary ammonium salt of chitosan. Carbohydr. Res..

[18] Martí M., Tuñón-Molina A., Aachmann F.L., Muramoto Y., Noda T., Takayama K., Serrano-Aroca Á. (2021). Protective face mask filter capable of inactivating SARS-CoV-2, and methicillin-resistant Staphylococcus aureus and Staphylococcus epidermidis. Polymers.

[19] Wang L., Chen J., Shi L., Shi Z., Ren L., Wang Y. (2014). The promotion of antimicrobial activity on silicon substrates using a “click” immobilized short peptide. Chem. Commun. (Camb)..

[20] Chongsiriwatana N.P., Patch J.A., Czyzewski A.M., Dohm M.T., Ivankin A., Gidalevitz D., Zuckermann R.N., Barron A.E. (2008). Peptoids that mimic the structure, function, and mechanism of helical antimicrobial peptides. Proc. Natl. Acad. Sci..

[21] Chen Y., Mant C.T., Farmer S.W., Hancock R.E.W., Vasil M.L., Hodges R.S. (2005). Rational design of alpha-helical antimicrobial peptides with enhanced activities and specificity/therapeutic index. J. Biol. Chem..

[22] Porter E.A., Wang X., Lee H.S., Weisblum B., Gellman S.H. (2000). Non-haemolytic beta-amino-acid oligomers. Nature.

[23] Salesa B., Martí M., Frígols B., Serrano-Aroca Á. (2019). Carbon nanofibers in pure form and in calcium alginate composites films: new cost-effective antibacterial biomaterials against the life-threatening multidrug-resistant Staphylococcus epidermidis. Polymers.

[24] Martí M., Frígols B., Salesa B., Serrano-Aroca Á. (2019). Calcium alginate/graphene oxide films: reinforced composites able to prevent Staphylococcus aureus and methicillin-resistant Staphylococcus epidermidis infections with no cytotoxicity for human keratinocyte HaCaT cells. Eur. Polym. J..

[25] Rivera-Briso A.L., Aachmann F.L., Moreno-Manzano V., Serrano-Aroca A. (2020). Graphene oxide nanosheets versus carbon nanofibers: enhancement of physical and biological properties of poly(3-hydroxybutyrate-co-3-hydroxyvalerate) films for biomedical applications. Int. J. Biol. Macromol..

[26] Frígols B., Martí M., Salesa B., Hernández-Oliver C., Aarstad O., Ulset A.S.T., Sætrom G.I., Aachmann F.L., Serrano-Aroca Á. (2019). Graphene oxide in zinc alginate films: antibacterial activity, cytotoxicity, zinc release, water sorption/diffusion, wettability and opacity. PLoS One.

[27] Liu J., Liu L., Wu X., Zhang X., Li T. (2015). Environmentally friendly synthesis of graphene-silver composites with surface-enhanced Raman scattering and antibacterial activity via reduction with l-ascorbic acid/water vapor. New J. Chem..

[28] Zhou L., Yang B., Sun C., Qiu X., Sun Z., Chen Y., Zhang Y., Dai Y. (2013). Coadministration of platelet-derived growth factor-BB and vascular endothelial growth factor with bladder acellular matrix enhances smooth muscle regeneration and vascularization for bladder augmentation in a rabbit model. Tissue Eng. Part A..

[29] Briquez P.S., Hubbell J.A., Martino M.M. (2015). Extracellular matrix-inspired growth factor delivery systems for skin wound healing. Adv. Wound Care.

[30] Med R. (2016). The role of small molecules in musculoskeletal regeneration. Regen. Med..

[31] O'Neill E., Awale G., Daneshmandi L., Umerah O., Lo K.W.H. (2018). The roles of ions on bone regeneration. Drug Discov. Today.

[32] Dorst K., Rammelkamp D., Hadjiargyrou M., Meng Y. (2014). The effect of exogenous zinc concentration on the responsiveness of MC3T3-E1 pre-osteoblasts to surface microtopography: Part II (differentiation). Materials.

[33] Wang T., Zhang J.C., Chen Y., Xiao P.G., Yang M.S. (2007). Effect of zinc ion on the osteogenic and adipogenic differentiation of mouse primary bone marrow stromal cells and the adipocytic trans-differentiation of mouse primary osteoblasts. J. Trace Elem. Med. Biol..

[34] Ma J., Zhao N., Zhu D. (2016). Bioabsorbable zinc ion induced biphasic cellular responses in vascular smooth muscle cells. Sci. Rep..

[35] Wu L., Feyerabend F., Schilling A.F., Willumeit-römer R., Luthringer B.J.C. (2015). Acta Biomaterialia Effects of extracellular magnesium extract on the proliferation and differentiation of human osteoblasts and osteoclasts in coculture. Acta Biomater..

[36] Zreiqat H., Howlett C.R., Zannettino A., Evans P., Knabe C., Shakibaei M. (2002). Mechanisms of magnesium-stimulated adhesion of osteoblastic cells to commonly used orthopaedic implants. J. Biomed. Mater. Res..

[37] Martin V., Ribeiro I.A., Alves M.M., Gonçalves L., Claudio R.A., Grenho L., Fernandes M.H., Gomes P., Santos C.F., Bettencourt A.F. (2019). Engineering a multifunctional 3D-printed PLA-collagen-minocycline-nanoHydroxyapatite scaffold with combined antimicrobial and osteogenic effects for bone regeneration. Mater. Sci. Eng. C..

[38] Ibrahim D.M., Sani E.S., Soliman A.M., Zandi N., Mostafavi E., Youssef A.M., Allam N.K., Annabi N. (2020). Bioactive and elastic nanocomposites with antimicrobial properties for bone tissue regeneration. ACS Appl. Bio Mater..

[39] Kargozar S., Montazerian M., Hamzehlou S., Kim H.W., Baino F. (2018). Mesoporous bioactive glasses: promising platforms for antibacterial strategies. Acta Biomater..

[40] Li Y., Chi Y.Q., Yu C.H., Xie Y., Xia M.Y., Zhang C.L., Han X., Peng Q. (2020). Drug-free and non-crosslinked chitosan scaffolds with efficient antibacterial activity against both Gram-negative and Gram-positive bacteria. Carbohydr. Polym..

[41] Radhika Rajasree S.R., Gobalakrishnan M., Aranganathan L., Karthih M.G. (2020). Fabrication and characterization of chitosan based collagen/gelatin composite scaffolds from big eye snapper Priacanthus hamrur skin for antimicrobial and anti oxidant applications. Mater. Sci. Eng. C..

[42] Liang W., Jiang M., Zhang J., Dou X., Zhou Y., Jiang Y., Zhao L., Lang M. (2021). Novel antibacterial cellulose diacetate-based composite 3D scaffold as potential wound dressing. J. Mater. Sci. Technol..

[43] Lanza R., Langer R., Vacanti J. (2014). Principles of Tissue Engineering.

[44] Smith L.A., Ma P.X. (2004). Nano-fibrous scaffolds for tissue engineering. Colloids Surfaces B Biointerfaces.

[45] Mikos A., Temenoff J. (2000). Formation of highly porous biodegradable scaffolds for tissue engineering, Electron. J. Biotechnol..

[46] El-Kady A.M., Rizk R.A., Abd El-Hady B.M., Shafaa M.W., Ahmed M.M. (2012). Characterization, and antibacterial properties of novel silver releasing nanocomposite scaffolds fabricated by the gas foaming/salt-leaching technique. J. Genet. Eng. Biotechnol..

[47] Rodríguez-Hernández J.C., Serrano-Aroca Á., Gómez-Ribelles J.L., Monleón-Pradas M. (2008). Three-dimensional nanocomposite scaffolds with ordered cylindrical orthogonal pores. J. Biomed. Mater. Res. Part B Appl. Biomater..

[48] Brígido-Diego R., Pérez-Olmedilla M., Serrano-Aroca Á., Gómez-Ribelles J.L., Monleón-Pradas M., Gallego-Ferrer G., Salmerón-Sánchez M. (2005). Acrylic scaffolds with interconnected spherical pores and controlled hydrophilicity for tissue engineering. J. Mater. Sci. Mater. Med..

[49] Wang N., Zhou Z., Xia L., Dai Y., Liu H. (2013). Fabrication and characterization of bioactive β-Ca2SiO4/PHBV composite scaffolds. Mater. Sci. Eng. C..

[50] Monleón-Pradas M., Gómez-Ribelles J.L., Serrano-Aroca Á., Gallego-Ferrer G., Suay-Antón J., Pissis P. (2001). Porous poly(2-hydroxyethyl acrylate) hydrogels. Polymer (Guildf).

[51] Serrano-Aroca Á., Monleón-Pradas M., Gómez-Ribelles J.L. (2007). Macroporous poly(methyl methacrylate) produced by phase separation during polymerisation in solution. Colloid Polym. Sci..

[52] Serrano-Aroca Á., Llorens-Gámez M. (2017). Dynamic mechanical analysis and water vapour sorption of highly porous poly(methyl methacrylate). Polymer (Guildf).

[53] Serrano-Aroca Á., Campillo-Fernández A.J., Gómez-Ribelles J.L., Monleón-Pradas M., Gallego-Ferrer G., Pissis P., Serrano Aroca A., Campillo Fernández A.J., Gómez Ribelles J.L., Monleón Pradas M., Gallego Ferrer G., Pissis P. (2004). Porous poly(2-hydroxyethyl acrylate) hydrogels prepared by radical polymerisation with methanol as diluent. Polymer (Guildf).

[54] Aroca A.S., Pradas M.M., Ribelles J.L.G. (2008). Effect of crosslinking on porous poly(methyl methacrylate) produced by phase separation. Colloid Polym. Sci..

[55] Martins A.M., Pham Q.P., Malafaya P.B., Sousa R.A., Gomes M.E., Raphael R.M., Kasper F.K., Reis R.L., Mikos A.G. (2009). The role of lipase and α-amylase in the degradation of starch/poly(Caprolactone) fiber meshes and the osteogenic differentiation of cultured marrow stromal cells. Tissue Eng. - Part A..

[56] Chen G., Ushida T., Tateishi T. (2001). Development of biodegradable porous scaffolds for tissue engineering. Mater. Sci. Eng. C..

[57] Zhang S. (2003). Fabrication of novel biomaterials through molecular self-assembly. Nat. Biotechnol..

[58] Shi X., Wang Y., Ren L., Zhao N., Gong Y., Wang D.A. (2009). Novel mesoporous silica-based antibiotic releasing scaffold for bone repair. Acta Biomater..

[59] Leong K.F., Cheah C.M., Chua C.K. (2003). Solid freeform fabrication of three-dimensional scaffolds for engineering replacement tissues and organs. Biomaterials.

[60] Maquet V., Jerome R. (1997). Design of macroporous biodegradable polymer scaffolds for cell transplantation. Mater. Sci. Forum.

[61] Serrano-Aroca Á., Ruiz-Pividal J.F.J.F., Llorens-Gámez M. (2017). Enhancement of water diffusion and compression performance of crosslinked alginate with a minuscule amount of graphene oxide. Sci. Rep..

[62] Llorens-Gámez M., Salesa B., Serrano-Aroca Á. (2020). Physical and biological properties of alginate/carbon nanofibers hydrogel films. Int. J. Biol. Macromol..

[63] Li J., Li L., Zhou J., Zhou Z., ling Wu X., Wang L., Yao Q. (2019). 3D printed dual-functional biomaterial with self-assembly micro-nano surface and enriched nano argentum for antibacterial and bone regeneration. Appl. Mater. Today.

[64] Lin L., Ju S., Cen L., Zhang H., Hu Q. (2008). Fabrication of porous β-TCP scaffolds by combination of rapid prototyping and freeze drying technology. IFMBE Proc.

[65] Garcia C., Gallardo A., López D., Elvira C., Azzahti A., Lopez-Martinez E., Cortajarena A.L., González-Henríquez C.M., Sarabia-Vallejos M.A., Rodríguez-Hernández J. (2018). Smart pH-responsive antimicrobial hydrogel scaffolds prepared by additive manufacturing. ACS Appl. Bio Mater..

[66] Malda J., Visser J., Melchels F.P., Jüngst T., Hennink W.E., Dhert W.J.A., Groll J., Hutmacher D.W. (2013). 25th anniversary article: engineering hydrogels for biofabrication. Adv. Mater..

[67] Vila-Parrondo C., García-Astrain C., Liz-Marzán L.M. (2020). Colloidal systems toward 3D cell culture scaffolds. Adv. Colloid Interface Sci..

[68] Leong M.F., Rasheed M.Z., Lim T.C., Chian K.S. (2009). In vitro cell infiltration and in vivo cell infiltration and vascularization in a fibrous, highly porous poly(D,L-lactide) scaffold fabricated by cryogenic electrospinning technique. J. Biomed. Mater. Res., Part A.

[69] Ohkawa K., Cha D., Kim H., Nishida A., Yamamoto H. (2004). Electrospinning of chitosan. Macromol. Rapid Commun..

[70] Rabea E.I., Badawy M.E.T., Stevens C.V., Smagghe G., Steurbaut W. (2003). Chitosan as antimicrobial agent: applications and mode of action. Biomacromolecules.

[71] Smith L.A., Beck J.A., Ma P.X. (2007). Nanofibrous scaffolds and their biological effects. Nanotechnologies Life Sci.

[72] Feng K., Sun H., Bradley M.A., Dupler E.J., Giannobile W.V., Ma P.X. (2010). Novel antibacterial nanofibrous PLLA scaffolds. J. Contr. Release.

[73] Mano J.F., Silva G.A., Azevedo H.S., Malafaya P.B., Sousa R.A., Silva S.S., Boesel L.F., Oliveira J.M., Santos T.C., Marques A.P., Neves N.M., Reis R.L. (2007). Natural origin biodegradable systems in tissue engineering and regenerative medicine: present status and some moving trends. J. R. Soc. Interface.

[74] Lee S.H., Kim B.S., Kim S.H., Kang S.W., Kim Y.H. (2004). Thermally produced biodegradable scaffolds for cartilage tissue engineering. Macromol. Biosci..

[75] Plikk P., Målberg S., Albertsson A.C. (2009). Design of resorbable porous tubular copolyester scaffolds for use in nerve regeneration. Biomacromolecules.

[76] Oh S.H., Kang S.G., Kim E.S., Cho S.H., Lee J.H. (2003). Fabrication and characterization of hydrophilic poly(lactic-co-glycolic acid)/poly(vinyl alcohol) blend cell scaffolds by melt-molding particulate-leaching method. Biomaterials.

[77] Se H.O., Soung G.K., Jin H.L. (2006). Degradation behavior of hydrophilized PLGA scaffolds prepared by melt-molding particulate-leaching method: comparison with control hydrophobic one. J. Mater. Sci. Mater. Med..

[78] Huang W., Shi X., Ren L., Du C., Wang Y. (2010). PHBV microspheres - PLGA matrix composite scaffold for bone tissue engineering. Biomaterials.

[79] Serrano-Aroca Á., Monleón-Pradas M., Gómez-Ribelles J.L. (2008). Effect of crosslinking on porous poly(methyl methacrylate) produced by phase separation. Colloid Polym. Sci..

[80] Monleón-Pradas M., Gómez-Ribelles J.L., Serrano-Aroca Á., Gallego-Ferrer G., Suay-Antón J., Pissis P. (2001). Interaction between water and polymer chains in poly(hydroxyethyl acrylate) hydrogels. Colloid Polym. Sci..

[81] Serrano-Aroca Á., Monleón-Pradas M., Gómez-Ribelles J.L. (2007). Plasma-induced polymerisation of hydrophilic coatings onto macroporous hydrophobic scaffolds. Polymer (Guildf).

[82] Serrano-Aroca Á., Gómez-Ribelles J.L., Monleón-Pradas M., Vidaurre-Garayo A., Suay-Antón J., Serrano Aroca A., Gómez Ribelles J.L., Monleón Pradas M., Vidaurre Garayo A., Suay Antón J. (2007). Characterisation of macroporous poly(methyl methacrylate) coated with plasma-polymerised poly(2-hydroxyethyl acrylate). Eur. Polym. J..

[83] Serrano-Aroca Á., Monleón-Pradas M., Gómez-Ribelles J.L., Rault J., Aroca A.S., Pradas M.M., Ribelles J.L.G., Rault J. (2015). Thermal analysis of water in reinforced plasma-polymerised poly(2-hydroxyethyl acrylate) hydrogels. Eur. Polym. J..

[84] Sánchez-Correa F., Vidaurre-Agut C., Serrano-Aroca A., Campillo-Fernández A.J. (2018). Poly(2-hydroxyethyl acrylate) hydrogels reinforced with graphene oxide: remarkable improvement of water diffusion and mechanical properties. J. Appl. Polym. Sci..

[85] Zhang S., Ellis-Behnke R., Zhao X. (2013). PuraMatrix: self-assembling peptide nanofiber scaffolds. A Chapter Scaffolding Tissue Eng.

[86] Mandal B.B., Kundu S.C. (2009). Cell proliferation and migration in silk fibroin 3D scaffolds. Biomaterials.

[87] Chen T.Y., Huang H.C., Cao J.L., Xin Y.J., Luo W.F., Ao N.J. (2016). Preparation and characterization of alginate/HACC/oyster shell powder biocomposite scaffolds for potential bone tissue engineering applications. RSC Adv..

[88] Cano-Vicent A., Tambuwala M.M., Hassan S.S., Barh D., Aljabali A.A.A., Birkett M., Arjunan A., Serrano-Aroca Á. (2021). Fused deposition modelling: current status, methodology, applications and future prospects. Addit. Manuf..

[89] Arjunan A., Robinson J., Baroutaji A., Tuñón-Molina A., Martí M., Serrano-Aroca Á. (2021). 3D printed cobalt-chromium-molybdenum porous superalloy with superior antiviral activity. Int. J. Mol. Sci..

[90] Robinson J., Arjunan A., Baroutaji A., Martí M., Tuñón Molina A., Serrano-Aroca Á., Pollard A. (2021). Additive manufacturing of anti-SARS-CoV-2 Copper-Tungsten-Silver alloy. Rapid Prototyp. J..

[91] Axpe E., Oyen M.L. (2016). Applications of alginate-based bioinks in 3D bioprinting. Int. J. Mol. Sci..

[92] Kang H.W., Lee S.J., Ko I.K., Kengla C., Yoo J.J., Atala A. (2016). A 3D bioprinting system to produce human-scale tissue constructs with structural integrity. Nat. Biotechnol..

[93] Jorgensen A.M., Yoo J.J., Atala A. (2020). Solid organ bioprinting: strategies to achieve organ function. Chem. Rev..

[94] Gao G., Huang Y., Schilling A.F., Hubbell K., Cui X. (2018). Organ bioprinting: are we there yet?. Adv. Healthc. Mater..

[95] Gao G., Park J.Y., Kim B.S., Jang J., Cho D.W. (2018). Coaxial cell printing of freestanding, perfusable, and functional in vitro vascular models for recapitulation of native vascular endothelium pathophysiology. Adv. Healthc. Mater..

[96] Zhu W., Qu X., Zhu J., Ma X., Patel S., Liu J., Wang P., Lai C.S.E., Gou M., Xu Y., Zhang K., Chen S. (2017). Direct 3D bioprinting of prevascularized tissue constructs with complex microarchitecture. Biomaterials.

[97] Albanna M., Binder K.W., Murphy S.V., Kim J., Qasem S.A., Zhao W., Tan J., El-Amin I.B., Dice D.D., Marco J., Green J., Xu T., Skardal A., Holmes J.H., Jackson J.D., Atala A., Yoo J.J. (2019). In situ bioprinting of autologous skin cells accelerates wound healing of extensive excisional full-thickness wounds. Sci. Rep..

[98] Augustine R. (2018). Skin bioprinting: a novel approach for creating artificial skin from synthetic and natural building blocks. Prog. Biomater..

[99] Wang Z., Lee S.J., Cheng H.J., Yoo J.J., Atala A. (2018). 3D bioprinted functional and contractile cardiac tissue constructs. Acta Biomater..

[100] Ong C.S., Fukunishi T., Zhang H., Huang C.Y., Nashed A., Blazeski A., Disilvestre D., Vricella L., Conte J., Tung L., Tomaselli G.F., Hibino N. (2017). Biomaterial-free three-dimensional bioprinting of cardiac tissue using human induced pluripotent stem cell derived cardiomyocytes. Sci. Rep..

[101] Chowdhury S.R., Keshavan N., Basu B. (2021).

[102] Abdollahiyan P., Oroojalian F., Mokhtarzadeh A., de la Guardia M. (2020). Hydrogel-based 3D bioprinting for bone and cartilage tissue engineering. Biotechnol. J..

[103] Ashammakhi N., Hasan A., Kaarela O., Byambaa B., Sheikhi A., Gaharwar A.K., Khademhosseini A. (2019). Advancing frontiers in bone bioprinting. Adv. Healthc. Mater..

[104] McCarthy R.R., Ullah M.W., Pei E., Yang G. (2019). Antimicrobial inks: the anti-infective applications of bioprinted bacterial polysaccharides. Trends Biotechnol..

[105] McCarthy R.R., Ullah M.W., Booth P., Pei E., Yang G. (2019). The use of bacterial polysaccharides in bioprinting. Biotechnol. Adv..

[107] Hinderer S., Brauchle E., Schenke-Layland K. (2015). Generation and assessment of functional biomaterial scaffolds for applications in cardiovascular tissue engineering and regenerative medicine. Adv. Healthc. Mater..

[108] Lukomska B., Stanaszek L., Zuba-Surma E., Legosz P., Sarzynska S., Drela K. (2019). Challenges and controversies in human mesenchymal stem cell therapy. Stem Cell. Int..

[109] Haider H.K., Lei Y., Ashraf M. (2008). MyoCell, a cell-based, autologous skeletal myoblast therapy for the treatment of cardiovascular diseases. Curr. Opin. Mol. Therapeut..

[110] Pettinato G., Perelman L.T., Fisher R.A. (2022). Methods Mol. Biol..

[111] Moreno-Manzano V., Mellado-López M., Morera-Esteve M.J., Alastrue-Agudo A., Bisbal-Velasco V., Forteza-Vila J., Serrano-Aroca Á., Vera-Donoso C.D. (2019). Human adipose-derived mesenchymal stem cells accelerate decellularized neobladder regeneration. Regen. Biomater..

[112] Minguell J.J., Erices A., Conget P. (2016).

[113] Panahi M., Rahimi B., Rahimi G., Yew Low T., Saraygord-Afshari N., Alizadeh E. (2020). Cytoprotective effects of antioxidant supplementation on mesenchymal stem cell therapy. J. Cell. Physiol..

[114] Mukheem A., Shahabuddin S., Akbar N., Anwar A., Sarih N.M., Sudesh K., Khan N.A., Sridewi N. (2020). Fabrication of biopolymer polyhydroxyalkanoate/chitosan and 2D molybdenum disulfide–doped scaffolds for antibacterial and biomedical applications. Appl. Microbiol. Biotechnol..

[115] Sumitha M.S., Shalumon K.T., Sreeja V.N., Jayakumar R., Nair S.V., Menon D. (2012). Biocompatible and antibacterial nanofibrous poly(ε-caprolactone)- nanosilver composite scaffolds for tissue engineering applications. J. Macromol. Sci. Part A Pure Appl. Chem..

[116] Brennan E.P., Reing J., Chew D., Myers-Irvin J.M., Young E.J., Badylak S.F. (2006). Antibacterial activity within degradation products of biological scaffolds composed of extracellular matrix. Tissue Eng..

[117] Mukheem A., Shahabuddin S., Akbar N., Miskon A., Sarih N.M., Sudesh K., Khan N.A., Saidur R., Sridewi N. (2019). Boron nitride doped polyhydroxyalkanoate/chitosan nanocomposite for antibacterial and biological applications. Nanomaterials.

[118] Hardiansyah A., Tanadi H., Yang M.C., Liu T.Y. (2015). Electrospinning and antibacterial activity of chitosan-blended poly(lactic acid) nanofibers. J. Polym. Res..

[119] Yuan H., Fernandes H., Habibovic P., De Boer J., Barradas A.M.C., De Ruiter A., Walsh W.R., Van Blitterswijk C.A., De Bruijn J.D. (2010). Osteoinductive ceramics as a synthetic alternative to autologous bone grafting. Proc. Natl. Acad. Sci. U. S. A..

[120] Martin V., Bettencourt A. (2018). Bone regeneration: biomaterials as local delivery systems with improved osteoinductive properties. Mater. Sci. Eng. C..

[121] Patrick S.M. (2013).

[122] Krishnan A.G., Jayaram L., Biswas R., Nair M. (2015). Evaluation of antibacterial activity and cytocompatibility of ciprofloxacin loaded gelatin-hydroxyapatite scaffolds as a local drug delivery system for osteomyelitis treatment. Tissue Eng. - Part A..

[123] García-Alvarez R., Izquierdo-Barba I., Vallet-Regí M. (2017). 3D scaffold with effective multidrug sequential release against bacteria biofilm. Acta Biomater..

[124] Bakhsheshi-Rad H.R., Hamzah E., Ismail A.F., Aziz M., Hadisi Z., Kashefian M., Najafinezhad A. (2017). Novel nanostructured baghdadite-vancomycin scaffolds: in-vitro drug release, antibacterial activity and biocompatibility. Mater. Lett..

[125] Cheng T., Qu H., Zhang G., Zhang X. (2018). Osteogenic and antibacterial properties of vancomycin-laden mesoporous bioglass/PLGA composite scaffolds for bone regeneration in infected bone defects. Artif. Cells, Nanomedicine Biotechnol..

[126] Zhou Z., Yao Q., Li L., Zhang X., Wei B., Yuan L., Wang L. (2018). Antimicrobial activity of 3D-printed poly(ε-Caprolactone) (PCL) composite scaffolds presenting vancomycin-loaded polylactic acid-glycolic acid (PLGA) microspheres. Med. Sci. Mon. Int. Med. J. Exp. Clin. Res..

[127] Wei J., Wang Y., Jiang J., Yan Y., Fan D., Yang X., Zuo Y., Li Y., Gu H., Li J. (2019). Development of an antibacterial bone graft by immobilization of levofloxacin hydrochloride-loaded mesoporous silica microspheres on a porous scaffold surface. J. Biomed. Nanotechnol..

[128] Paris J.L., Lafuente-Gómez N., Cabañas M.V., Román J., Peña J., Vallet-Regí M. (2019). Fabrication of a nanoparticle-containing 3D porous bone scaffold with proangiogenic and antibacterial properties. Acta Biomater..

[129] Wu P., Hu S., Liang Q., Guo W., Xia Y., Shuai C., Li Y. (2020). A polymer scaffold with drug-sustained release and antibacterial activity. Int. J. Polym. Mater. Polym. Biomater..

[130] Liu D., Liu Z., Zou J., Li L., Sui X., Wang B., Yang N., Wang B. (2021). Synthesis and characterization of a hydroxyapatite-sodium alginate-chitosan scaffold for bone regeneration. Front. Mater..

[131] Orafa Z., Bakhshi H., Arab-Ahmadi S., Irani S. (2022). Laponite/amoxicillin-functionalized PLA nanofibrous as osteoinductive and antibacterial scaffolds. Sci. Rep..

[132] Wang Q., Yu X., Libera M. (2013). Reducing bacterial colonization of 3-D nanofiber cell scaffolds by hierarchical assembly of microgels and an antimicrobial peptide. Adv. Healthc. Mater..

[133] Zhou P., Xia Y., Jiang L., Zhang Y., Qiu C., Xie Y., Xu S. (2016). O-Acrylamidomethyl-2-hydroxypropyltrimethyl ammonium chloride chitosan and silk modified mesoporous bioactive glass scaffolds with excellent mechanical properties, bioactivity and long-lasting antibacterial activity. RSC Adv..

[134] Yang Y., Chu L., Yang S., Zhang H., Qin L., Guillaume O., Eglin D., Richards R.G., Tang T. (2018). Dual-functional 3D-printed composite scaffold for inhibiting bacterial infection and promoting bone regeneration in infected bone defect models. Acta Biomater..

[135] Li X., Wang Y., Guo M., Wang Z., Shao N., Zhang P., Chen X., Huang Y. (2018). Degradable three dimensional-printed polylactic acid scaffold with long-term antibacterial activity. ACS Sustain. Chem. Eng..

[136] Pant J., Sundaram J., Goudie M.J., Nguyen D.T., Handa H. (2019). Antibacterial 3D bone scaffolds for tissue engineering application. J. Biomed. Mater. Res. Part B Appl. Biomater..

[137] He Y., Jin Y., Ying X., Wu Q., Yao S., Li Y., Liu H., Ma G., Wang X. (2020). Development of an antimicrobial peptide-loaded mineralized collagen bone scaffold for infective bone defect repair. Regen. Biomater..

[138] Tian L., Zhang Z., Tian B., Zhang X., Wang N. (2020). Study on antibacterial properties and cytocompatibility of EPL coated 3D printed PCL/HA composite scaffolds. RSC Adv..

[139] Hu J., Wang Z., Miszuk J.M., Zhu M., Lansakara T.I., Tivanski A.V., Banas J.A., Sun H. (2021). Vanillin-bioglass cross-linked 3D porous chitosan scaffolds with strong osteopromotive and antibacterial abilities for bone tissue engineering. Carbohydr. Polym..

[140] Karamat-Ullah N., Demidov Y., Schramm M., Grumme D., Auer J., Bohr C., Brachvogel B., Maleki H. (2021). 3D printing of antibacterial, biocompatible, and biomimetic hybrid aerogel-based scaffolds with hierarchical porosities via integrating antibacterial peptide-modified silk fibroin with silica nanostructure. ACS Biomater. Sci. Eng..

[141] Ye Z., Zhu X., Mutreja I., Boda S.K., Fischer N.G., Zhang A., Lui C., Qi Y., Aparicio C. (2021). Biomimetic mineralized hybrid scaffolds with antimicrobial peptides. Bioact. Mater..

[142] Rama M., Vijayalakshmi U. (2022). Biological and mechanical investigation of novel flax/silk protein-based nanofibrous scaffold for bone regeneration. Prog. Nat. Sci. Mater. Int..

[143] Zanin H., Rodrigues B.V.M., Ribeiro Neto W.A., Bretas R.E.S., Da-Silva N.S., Marciano F.R., Oliveira Lobo A. (2016). High loading of graphene oxide/multi-walled carbon nanotubes into PDLLA: a route towards the design of osteoconductive, bactericidal and non-immunogenic 3D porous scaffolds. Mater. Chem. Phys..

[144] Ouyang L., Deng Y., Yang L., Shi X., Dong T., Tai Y., Yang W., Chen Z.G. (2018). Graphene-oxide-decorated microporous polyetheretherketone with superior antibacterial capability and in vitro osteogenesis for orthopedic implant. Macromol. Biosci..

[145] Angulo-Pineda C., Srirussamee K., Palma P., Fuenzalida V.M., Cartmell S.H., Palza H. (2020). Electroactive 3D printed scaffolds based on percolated composites of polycaprolactone with thermally reduced graphene oxide for antibacterial and tissue engineering applications. Nanomaterials.

[146] Melo S.F., Neves S.C., Pereira A.T., Borges I., Granja P.L., Magalhães F.D., Gonçalves I.C. (2020). Incorporation of graphene oxide into poly(ϵ-caprolactone) 3D printed fibrous scaffolds improves their antimicrobial properties. Mater. Sci. Eng. C..

[147] Lu H., Pan X., Hu M., Zhang J., Yu Y., Hu X., Jiang K. (2021). Fabrication of graphene/gelatin/chitosan/tricalcium phosphate 3D printed scaffolds for bone tissue regeneration applications. Appl. Nanosci..

[148] Aslam Khan M.U., Haider A., Abd Razak S.I., Abdul Kadir M.R., Haider S., Shah S.A., Hasan A., Khan R., ud din Khan S., Shakir I. (2021). Arabinoxylan/graphene-oxide/nHAp-NPs/PVA bionano composite scaffolds for fractured bone healing. J. Tissue Eng. Regen. Med..

[149] Umar Aslam Khan M., Haider S., Haider A., Izwan Abd Razak S., Rafiq Abdul Kadir M., Shah S.A., Javed A., Shakir I., Al-Zahrani A.A. (2021). Development of porous, antibacterial and biocompatible GO/n-HAp/bacterial cellulose/β-glucan biocomposite scaffold for bone tissue engineering. Arab. J. Chem..

[150] Najafinezhad A., Bakhsheshi-Rad H.R., Saberi A., Nourbakhsh A.A., Daroonparvar M., Ismail A.F., Sharif S., Ramakrishna S., Dai Y., Berto F. (2022). Graphene oxide encapsulated forsterite scaffolds to improve mechanical properties and antibacterial behavior, Biomed. Mater.

[151] Schneider O.D., Loher S., Brunner T.J., Schmidlin P., Stark W.J. (2008). Flexible, silver containing nanocomposites for the repair of bone defects: antimicrobial effect against E. coli infection and comparison to tetracycline containing scaffolds. J. Mater. Chem..

[152] Vitale-Brovarone C., Miola M., Balagna C., Verné E. (2008). 3D-glass-ceramic scaffolds with antibacterial properties for bone grafting. Chem. Eng. J..

[153] FGorriti M., Lopez J.M.P., Boccaccini A.R., Audisio C., Gorustovich A.A. (2009). In vitro study glassceramic of the antibacterial activity of bioactive scaffolds. Adv. Eng. Mater..

[154] Wu X., Li J., Wang L., Huang D., Zuo Y., Li Y. (2010). Biomed. Mater..

[155] Zhang Y., Yin Q.S., Xia H., Ai F.Z., Jiao Y.P., Chen X.Q. (2010). Determination of antibacterial properties and cytocompatibility of silver-loaded coral hydroxyapatite. J. Mater. Sci. Mater. Med..

[156] Balagna C., Vitale-Brovarone C., Miola M., Verné E., Canuto R.A., Saracino S., Muzio G., Fucale G., Maina G. (2011). Biocompatibility and antibacterial effect of silver doped 3D-glass-ceramic scaffolds for bone grafting. J. Biomater. Appl..

[157] Wu C., Zhou Y., Xu M., Han P., Chen L., Chang J., Xiao Y. (2013). Copper-containing mesoporous bioactive glass scaffolds with multifunctional properties of angiogenesis capacity, osteostimulation and antibacterial activity. Biomaterials.

[158] Marsich E., Bellomo F., Turco G., Travan A., Donati I., Paoletti S. (2013). Nano-composite scaffolds for bone tissue engineering containing silver nanoparticles: preparation, characterization and biological properties. J. Mater. Sci. Mater. Med..

[159] Smoak M., Chen C., Qureshi A., Garber L., Pojman J.A., Janes M.E., Hayes D.J. (2014). Antimicrobial cytocompatible pentaerythritol triacrylate-co-trimethylolpropane composite scaffolds for orthopaedic implants. J. Appl. Polym. Sci..

[160] Yazdimamaghani M., Vashaee D., Assefa S., Walker K.J., Madihally S.V., Köhler G.A., Tayebi L. (2014). Hybrid macroporous gelatin/bioactive-glass/nanosilver scaffolds with controlled degradation behavior and antimicrobial activity for bone tissue engineering. J. Biomed. Nanotechnol..

[161] Sánchez-Salcedo S., Shruti S., Salinas A.J., Malavasi G., Menabue L., Vallet-Regí M. (2014). In vitro antibacterial capacity and cytocompatibility of SiO 2-CaO-P2O5 meso-macroporous glass scaffolds enriched with ZnO. J. Mater. Chem. B..

[162] Ma R., Lai Y.X., Li L., Tan H.L., Wang J.L., Li Y., Tang T.T., Qin L. (2015). Bacterial inhibition potential of 3D rapid-prototyped magnesium-based porous composite scaffolds - an in vitro efficacy study. Sci. Rep..

[163] Stevanović M., Filipović N., Djurdjević J., Lukić M., Milenković M., Boccaccini A. (2015). 45S5Bioglass®-based scaffolds coated with selenium nanoparticles or with poly(lactide-co-glycolide)/selenium particles: processing, evaluation and antibacterial activity. Colloids Surfaces B Biointerfaces.

[164] Abdel-Ghany B.E., Abdel-Hady B.M., El-Kady A.M., Beheiry H.H., Guirguis O.W. (2015). Characterizations of nano-zinc doped hydroxyapatite to use as bone tissue engineering. Adv. Mater. Res..

[165] Wang H., Zhao S., Cui X., Pan Y., Huang W., Ye S., Luo S., Rahaman M.N., Zhang C., Wang D. (2015). Evaluation of three-dimensional silver-doped borate bioactive glass scaffolds for bone repair: biodegradability, biocompatibility, and antibacterial activity. J. Mater. Res..

[166] Jiang J., Li L., Li K., Li G., You F., Zuo Y., Li Y., Li J. (2016). Antibacterial nanohydroxyapatite/polyurethane composite scaffolds with silver phosphate particles for bone regeneration. J. Biomater. Sci. Polym. Ed..

[167] Sehgal R.R., Carvalho E., Banerjee R. (2016). Mechanically stiff, zinc cross-linked nanocomposite scaffolds with improved osteostimulation and antibacterial properties. ACS Appl. Mater. Interfaces.

[168] Lu M., Liao J., Dong J., Wu J., Qiu H., Zhou X., Li J., Jiang D., He T.C., Quan Z. (2016). An effective treatment of experimental osteomyelitis using the antimicrobial titanium/silver-containing nHP66 (nano-hydroxyapatite/polyamide-66) nanoscaffold biomaterials. Sci. Rep..

[169] Zeimaran E., Pourshahrestani S., Djordjevic I., Pingguan-Murphy B., Kadri N.A., Wren A.W., Towler M.R. (2016). Antibacterial properties of poly (octanediol citrate)/gallium-containing bioglass composite scaffolds. J. Mater. Sci. Mater. Med..

[170] Miola M., Verné E., Vitale-Brovarone C., Baino F. (2016). Antibacterial bioglass-derived scaffolds: innovative synthesis approach and characterization. Int. J. Appl. Glass Sci..

[171] Esteban-Tejeda L., Zheng K., Prado C., Cabal B., Torrecillas R., Boccaccini A.R., Moya J.S. (2016). Bone tissue scaffolds based on antimicrobial SiO2-Na2O-Al2O3-CaO-B2O3 glass. J. Non-Cryst. Solids.

[172] Amin Yavari S., Loozen L., Paganelli F.L., Bakhshandeh S., Lietaert K., Groot J.A., Fluit A.C., Boel C.H.E., Alblas J., Vogely H.C., Weinans H., Zadpoor A.A. (2016).

[173] Deng L., Deng Y., Xie K. (2017). AgNPs-decorated 3D printed PEEK implant for infection control and bone repair. Colloids Surfaces B Biointerfaces.

[174] Gao Y., Hassanbhai A.M., Lim J., Wang L., Xu C. (2017). Fabrication of a silver octahedral nanoparticle-containing polycaprolactone nanocomposite for antibacterial bone scaffolds. RSC Adv..

[175] Publikācija: Preparation and Antibacterial Properties of Silver Doped Hydroxyapatite Scaffolds, (n.d.).

[176] Zhang Y., Zhai D., Xu M., Yao Q., Zhu H., Chang J., Wu C. (2017). 3D-printed bioceramic scaffolds with antibacterial and osteogenic activity. Biofabrication.

[177] Wang Q., Tang P., Ge X., Li P., Lv C., Wang M., Wang K., Fang L., Lu X. (2018). Experimental and simulation studies of strontium/zinc-codoped hydroxyapatite porous scaffolds with excellent osteoinductivity and antibacterial activity. Appl. Surf. Sci..

[178] Kiran A.S.K., Kumar T.S.S., Sanghavi R., Doble M., Ramakrishna S. (2018). Antibacterial and bioactive surface modifications of titanium implants by PCL/TiO2 nanocomposite coatings. Nanomaterials.

[179] Wang Y., Gao Y., Xu G., Liu H., Xiang Y., Cui W. (2018). Accelerated fabrication of antibacterial and osteoinductive electrospun fibrous scaffolds: via electrochemical deposition. RSC Adv..

[180] Shuai C., Shuai C., Feng P., Gao C., Peng S., Yang Y. (2018). Antibacterial capability, physicochemical properties, and biocompatibility of nTiO2 incorporated polymeric scaffolds. Polymers.

[181] Wiedmer D., Cui C., Weber F., Petersen F.C., Tiainen H. (2018). Antibacterial surface coating for bone scaffolds based on the dark catalytic effect of titanium dioxide. ACS Appl. Mater. Interfaces.

[182] Felice B., Sánchez M.A., Socci M.C., Sappia L.D., Gómez M.I., Cruz M.K., Felice C.J., Martí M., Pividori M.I., Simonelli G., Rodríguez A.P. (2018). Controlled degradability of PCL-ZnO nanofibrous scaffolds for bone tissue engineering and their antibacterial activity. Mater. Sci. Eng. C..

[183] El-Rashidy A.A., Waly G., Gad A., Roether J.A., Hum J., Yang Y., Detsch R., Hashem A.A., Sami I., Goldmann W.H., Boccaccini A.R. (2018). Antibacterial activity and biocompatibility of zein scaffolds containing silver-doped bioactive glass. Biomed. Mater..

[184] Bagri L.P., Saini R.K., Kumar Bajpai A., Choubey R. (2019). Silver hydroxyapatite reinforced poly(vinyl alcohol)—starch cryogel nanocomposites and study of biodegradation, compressive strength and antibacterial activity. Polym. Eng. Sci..

[185] Touri M., Moztarzadeh F., Osman N.A.A., Dehghan M.M., Mozafari M. (2019). Optimisation and biological activities of bioceramic robocast scaffolds provided with an oxygen-releasing coating for bone tissue engineering applications. Ceram. Int..

[186] Li K., Cai K., Ran Q., Jiang D. (2019). Biomimetic triphase composite scaffolds with antibacterial and anti-tumor potentials for bone repair. Mater. Lett..

[187] Wang S., Li R., Qing Y., Wei Y., Wang Q., Zhang T., Sun C., Qin Y., Li D., Yu J. (2019). Antibacterial activity of Ag-incorporated zincosilicate zeolite scaffolds fabricated by additive manufacturing. Inorg. Chem. Commun..

[188] Kumar Saini R., Prasad Bagri L., Bajpai A.K. (2019). Nano-silver hydroxyapatite based antibacterial 3D scaffolds of gelatin/alginate/poly (vinyl alcohol) for bone tissue engineering applications. Colloids Surfaces B Biointerfaces.

[189] Patil S., Singh N. (2019). Antibacterial silk fibroin scaffolds with green synthesized silver nanoparticles for osteoblast proliferation and human mesenchymal stem cell differentiation. Colloids Surfaces B Biointerfaces.

[190] L. G, Q. S, Z. D, L. X (2019). Preparation of antibacterial degummed silk fiber/nano-hydroxyapatite/polylactic acid composite scaffold by degummed silk fiber loaded silver nanoparticles. Nanotechnology.

[191] Paterson T.E., Shi R., Tian J., Harrison C.J., De Sousa Mendes M., Hatton P.V., Li Z., Ortega I. (2020). Electrospun scaffolds containing silver-doped hydroxyapatite with antimicrobial properties for applications in orthopedic and dental bone surgery. J. Funct. Biomater..

[192] Zhang L., Jia G., Tang M., Chen C., Niu J., Huang H., Kang B., Pei J., Zeng H., Yuan G. (2020). Simultaneous enhancement of anti-corrosion, biocompatibility, and antimicrobial activities by hierarchically-structured brushite/Ag3PO4-coated Mg-based scaffolds. Mater. Sci. Eng. C..

[193] Arjunan A., Robinson J., Al Ani E., Heaselgrave W., Baroutaji A., Wang C. (2020). Mechanical performance of additively manufactured pure silver antibacterial bone scaffolds. J. Mech. Behav. Biomed. Mater..

[194] Zou F., Jiang J., Lv F., Xia X., Ma X. (2020). Preparation of antibacterial and osteoconductive 3D-printed PLGA/Cu(I)@ZIF-8 nanocomposite scaffolds for infected bone repair. J. Nanobiotechnol..

[195] Luo Y., Humayun A., Mills D.K. (2020). Surface modification of 3D printed PLA/halloysite composite scaffolds with antibacterial and osteogenic capabilities. Appl. Sci..

[196] Sun H., Hu C., Zhou C., Wu L., Sun J., Zhou X., Xing F., Long C., Kong Q., Liang J., Fan Y., Zhang X. (2020). 3D printing of calcium phosphate scaffolds with controlled release of antibacterial functions for jaw bone repair. Mater. Des..

[197] Suárez M., Fernández-García E., Fernández A., López-Píriz R., Díaz R., Torrecillas R. (2020). Novel antimicrobial phosphate-free glass–ceramic scaffolds for bone tissue regeneration. Sci. Rep..

[198] Shuai C., Wang C., Qi F., Peng S., Yang W., He C., Wang G., Qian G. (2020). Enhanced crystallinity and antibacterial of PHBV scaffolds incorporated with zinc oxide. J. Nanomater..

[199] Zhu T., Zhu M., Zhu Y. (2020). Fabrication of forsterite scaffolds with photothermal-induced antibacterial activity by 3D printing and polymer-derived ceramics strategy. Ceram. Int..

[200] Khan M.U.A., Al-Thebaiti M.A., Hashmi M.U., Aftab S., Razak S.I.A., Hassan S.A., Kadir M.R.A., Amin R. (2020). Synthesis of silver-coated bioactive nanocomposite scaffolds based on grafted beta-glucan/hydroxyapatite via freeze-drying method: anti-microbial and biocompatibility evaluation for bone tissue engineering. Materials.

[201] Bakhsheshi-Rad H.R., Najafinezhad A., Hadisi Z., Iqbal N., Daroonparvar M., Sharif S., Ismail A.F., Akbari M., RamaKrishna S., Berto F. (2021). Characterization and biological properties of nanostructured clinoenstatite scaffolds for bone tissue engineering applications. Mater. Chem. Phys..

[202] Radhakrishnan S., Nagarajan S., Belaid H., Farha C., Iatsunskyi I., Coy E., Soussan L., Huon V., Bares J., Belkacemi K., Teyssier C., Balme S., Miele P., Cornu D., Kalkura N., Cavaillès V., Bechelany M. (2021). Fabrication of 3D printed antimicrobial polycaprolactone scaffolds for tissue engineering applications. Mater. Sci. Eng. C..

[203] Hayashi K., Shimabukuro M., Ishikawa K. (2022). Antibacterial honeycomb scaffolds for achieving infection prevention and bone regeneration. ACS Appl. Mater. Interfaces.

[204] Kim H.-I., Raja N., Kim J., Sung A., Choi Y.-J., Yun H., Park H. (2022). A 3D calcium-deficient hydroxyapatite-based scaffold with gold nanoparticles effective against Micrococcus luteus as an artificial bone substitute. Mater. Des..

[205] Wang L., Yu B., Sun L.P., Ren L., Zhang Q.Q. (2008). Microsphere-integrated gelatin-siloxane hybrid scaffolds for bone tissue engineering: in vitro bioactivity & antibacterial activity. Front. Mater. Sci. China.

[206] Ma T., Shang B.C., Tang H., Zhou T.H., Xu G.L., Li H.L., Chen Q.H., Xu Y.Q. (2011). Nano-hydroxyapatite/chitosan/konjac glucomannan scaffolds loaded with cationic liposomal vancomycin: preparation, in vitro release and activity against staphylococcus aureus biofilms. J. Biomater. Sci. Polym. Ed..

[207] Zhou P., Xia Y., Wang J., Liang C., Yu L., Tang W., Gu S., Xu S. (2013). Antibacterial properties and bioactivity of HACC- and HACC-Zein-modified mesoporous bioactive glass scaffolds. J. Mater. Chem. B..

[208] S. Thanyaphoo, Characterization of Antimicrobial and Bone Regenerative Activities of Porous Si-nHA Scaffolds Containing Vancomycin and rhBMP2, (n.d).

[209] Xie C.M., Lu X., Wang K.F., Meng F.Z., Jiang O., Zhang H.P., Zhi W., Fang L.M. (2014). Silver nanoparticles and growth factors incorporated hydroxyapatite coatings on metallic implant surfaces for enhancement of osteoinductivity and antibacterial properties. ACS Appl. Mater. Interfaces.

[210] Li W., Wang H., Ding Y., Scheithauer E.C., Goudouri O.M., Grünewald A., Detsch R., Agarwal S., Boccaccini A.R. (2015). Antibacterial 45S5 Bioglass®-based scaffolds reinforced with genipin cross-linked gelatin for bone tissue engineering. J. Mater. Chem. B..

[211] Xu Z.L., Lei Y., Yin W.J., Chen Y.X., Ke Q.F., Guo Y.P., Zhang C.Q. (2016). Enhanced antibacterial activity and osteoinductivity of Ag-loaded strontium hydroxyapatite/chitosan porous scaffolds for bone tissue engineering. J. Mater. Chem. B..

[212] Correia T.R., Figueira D.R., de Sá K.D., Miguel S.P., Fradique R.G., Mendonça A.G., Correia I.J. (2016). 3D Printed scaffolds with bactericidal activity aimed for bone tissue regeneration. Int. J. Biol. Macromol..

[213] Bakhshandeh S., Gorgin Karaji Z., Lietaert K., Fluit A.C., Boel C.H.E., Vogely H.C., Vermonden T., Hennink W.E., Weinans H., Zadpoor A.A., Amin Yavari S. (2017).

[214] Bakhsheshi-Rad H.R., Hamzah E., Abbasizadeh N., Najafinezhad A., Kashefian M. (2018). Synthesis of novel nanostructured bredigite–amoxicillin scaffolds for bone defect treatment: cytocompatibility and antibacterial activity. J. Sol. Gel Sci. Technol..

[215] García-González C.A., Barros J., Rey-Rico A., Redondo P., Gómez-Amoza J.L., Concheiro A., Alvarez-Lorenzo C., Monteiro F.J. (2018). Antimicrobial properties and osteogenicity of vancomycin-loaded synthetic scaffolds obtained by supercritical foaming. ACS Appl. Mater. Interfaces.

[216] Qian Y., Zhou X., Sun H., Yang J., Chen Y., Li C., Wang H., Xing T., Zhang F., Gu N. (2018). Biomimetic domain-active electrospun scaffolds facilitating bone regeneration synergistically with antibacterial efficacy for bone defects. ACS Appl. Mater. Interfaces.

[217] Shuai C., Guo W., Wu P., Yang W., Hu S., Xia Y., Feng P. (2018). A graphene oxide-Ag co-dispersing nanosystem: dual synergistic effects on antibacterial activities and mechanical properties of polymer scaffolds. Chem. Eng. J..

[218] Bakhsheshi-Rad H.R., Hamzah E., Staiger M.P., Dias G.J., Hadisi Z., Saheban M., Kashefian M. (2018). Drug release, cytocompatibility, bioactivity, and antibacterial activity of doxycycline loaded Mg-Ca-TiO2 composite scaffold. Mater. Des..

[219] Anisimova N.Y., Zalepugin D.Y., Chernyshova I.V., Maksimkin A.V., Kiselevskii M.V., Senatov F.S., Spirina T.S., Sitdikova S.M., Karaulov A.V. (2019). Antibacterial activity of hybrid polymeric scaffold for reconstruction of tubular bone defects. Bull. Exp. Biol. Med..

[220] Bakhsheshi-Rad H.R., Chen X.B., Ismail A.F., Aziz M., Hamzah E., Najafinezhad A. (2019). A new multifunctional monticellite-ciprofloxacin scaffold: preparation, bioactivity, biocompatibility, and antibacterial properties. Mater. Chem. Phys..

[221] Dayaghi E., Bakhsheshi-Rad H.R., Hamzah E., Akhavan-Farid A., Ismail A.F., Aziz M., Abdolahi E. (2019). Magnesium-zinc scaffold loaded with tetracycline for tissue engineering application: in vitro cell biology and antibacterial activity assessment. Mater. Sci. Eng. C..

[222] Khan M.U.A., Mehboob H., Abd Razak S.I., Yahya M.Y., Yusof A.H.M., Ramlee M.H., Anand T.J.S., Hassan R., Aziz A., Amin R. (2020). Development of polymeric nanocomposite (xyloglucan-co-methacrylic acid/hydroxyapatite/SiO2) scaffold for bone tissue engineering applications-in-vitro antibacterial, cytotoxicity and cell culture evaluation. Polymers.

[223] Hu C., Wu L., Zhou C., Sun H., Gao P., Xu X., Zhang C., Liang J., Fan Y., Sun J., Zhou X., Zhang X. (2020). Berberine/Ag nanoparticle embedded biomimetic calcium phosphate scaffolds for enhancing antibacterial function. Nanotechnol. Rev..

[224] Maleki-Ghaleh H., Siadati M.H., Fallah A., Koc B., Kavanlouei M., Khademi-Azandehi P., Moradpur-Tari E., Omidi Y., Barar J., Beygi-Khosrowshahi Y., Kumar A.P., Adibkia K. (2021). Antibacterial and cellular behaviors of novel zinc-doped hydroxyapatite/graphene nanocomposite for bone tissue engineering. Int. J. Mol. Sci..

[225] Saxena V., Hasan A., Pandey L.M. (2021). Antibacterial nano-biocomposite scaffolds of Chitosan, Carboxymethyl Cellulose and Zn & Fe integrated Hydroxyapatite (Chitosan-CMC-FZO@HAp) for bone tissue engineering. Cellulose.

[226] Yu L., He T., Yao J., Xu W., Peng S., Feng P., Shuai C. (2022). Cu ions and cetyltrimethylammonium bromide loaded into montmorillonite: a synergistic antibacterial system for bone scaffolds. Mater. Chem. Front..

[227] Yu Z., Xu Y., Tian X. (2022). Silver-modified graphene oxide nanosheets for antibacterial performance of bone scaffold. AIP Adv..

[228] Khan M.U.A., Rizwan M., Razak S.I.A., Hassan A., Rasheed T., Bilal M. (2022). Electroactive polymeric nanocomposite BC-g-(Fe3O4/GO) materials for bone tissue engineering: in vitro evaluations. J. Biomater. Sci. Polym. Ed..

[229] Zhang X., He J., Qiao | Liang, Wang Z., Zheng Q., Xiong C., Yang H., Li K., Lu C., Li S., Chen H., Hu X. (2022). 3D printed PCLA scaffold with nano-hydroxyapatite coating doped green tea EGCG promotes bone growth and inhibits multidrug-resistant bacteria colonization. Cell Prolif.

[230] Khan M.U.A., Haider S., Shah S.A., Razak S.I.A., Hassan S.A., Kadir M.R.A., Haider A. (2020). Arabinoxylan-co-AA/HAp/TiO2 nanocomposite scaffold a potential material for bone tissue engineering: an in vitro study. Int. J. Biol. Macromol..

[231] Aslam Khan M.U., Raza M.A., Mehboob H., Abdul Kadir M.R., Abd Razak S.I., Shah S.A., Iqbal M.Z., Amin R. (2020). Development and: in vitro evaluation of κ-carrageenan based polymeric hybrid nanocomposite scaffolds for bone tissue engineering. RSC Adv..

[232] Aslam Khan M.U., Al-Arjan W.S., Binkadem M.S., Mehboob H., Haider A., Raza M.A., Abd Razak S.I., Hasan A., Amin R. (2021). Development of biopolymeric hybrid scaffold-based on aac/go/nhap/tio2 nanocomposite for bone tissue engineering: in-vitro analysis. Nanomaterials.

[233] Ge Y.-W., Fan Z.-H., Ke Q.-F., Guo Y.-P., Zhang C.-Q., Jia W.-T. (2022). SrFe12O19-doped nano-layered double hydroxide/chitosan layered scaffolds with a nacre-mimetic architecture guide in situ bone ingrowth and regulate bone homeostasis. Mater. Today Bio..

[234] Mouriño V., Boccaccini A.R. (2010). Bone tissue engineering therapeutics: controlled drug delivery in three-dimensional scaffolds. J. R. Soc. Interface.

[235] Bohlouli M., Tamjid E., Mohammadi S., Nikkhah M. (2020). Original research A study on cytotoxicity, hemocompatibility, and antibacterial properties of tetracycline hydrochloride-loaded PCL-based composite scaffolds for bone tissue engineering applications. Modares Journal of Biotechnology.

[236] Mortazavi M.M., Khan M.A., Quadri S.A., Suriya S.S., Fahimdanesh K.M., Fard S.A., Hassanzadeh T., Taqi M.A., Grossman H., Tubbs R.S. (2018). Cranial osteomyelitis: a comprehensive review of modern therapies. World Neurosurg.

[237] Muthukrishnan L. (2021). Imminent antimicrobial bioink deploying cellulose, alginate, EPS and synthetic polymers for 3D bioprinting of tissue constructs. Carbohydr. Polym..

[238] Tao F., Cheng Y., Shi X., Zheng H., Du Y., Xiang W., Deng H. (2020). Applications of chitin and chitosan nanofibers in bone regenerative engineering. Carbohydr. Polym..

[239] Lobo F.C.M., Franco A.R., Fernandes E.M., Reis R.L. (2021). An overview of the antimicrobial properties of lignocellulosic materials. Molecules.

[240] Romanò C.L., Scarponi S., Gallazzi E., Romanò D., Drago L. (2015). Antibacterial coating of implants in orthopaedics and trauma: a classification proposal in an evolving panorama. J. Orthop. Surg. Res..

[241] Fernandes M.M., Carvalho E.O., Lanceros-Mendez S. (2019). Electroactive smart materials: novel tools for tailoring bacteria behavior and fight antimicrobial resistance. Front. Bioeng. Biotechnol..

[242] Hitscherich P., Aphale A., Gordan R., Whitaker R., Singh P., hua Xie L., Patra P., Lee E.J. (2018). Electroactive graphene composite scaffolds for cardiac tissue engineering. J. Biomed. Mater. Res., Part A.

[243] Aparicio-Collado J.L., García-San Martín N., Molina-Mateo J., Torregrosa Cabanilles C., Donderis Quiles V., Serrano-Aroca A., Sabater i Serra R. (2022). Electroactive calcium-alginate/polycaprolactone/reduced graphene oxide nanohybrid hydrogels for skeletal muscle tissue engineering. Colloids Surfaces B Biointerfaces.

[244] Voegele P., Badiola J., Schmidt-Malan S.M., Karau M.J., Greenwood-Quaintance K.E., Mandrekar J.N., Patel R. (2016). Antibiofilm activity of electrical current in a catheter model. Antimicrob. Agents Chemother..

[245] Elias L., Taengua R., Frígols B., Salesa B., Serrano-Aroca Á. (2019). Carbon nanomaterials and LED irradiation as antibacterial strategies against gram-positive multidrug-resistant pathogens. Int. J. Mol. Sci..

[246] Salesa B., Assis M., Andrés J., Serrano-Aroca Á. (2021). Carbon nanofibers versus silver nanoparticles: time-dependent cytotoxicity, proliferation, and gene expression. Biomedicines.

[247] Salesa B., Serrano-Aroca Á. (2021). Multi-layer graphene oxide in human keratinocytes: time-dependent cytotoxicity, proliferation, and gene expression. Coatings.

[248] Llorens-Gámez M., Serrano-Aroca Á. (2018). Low-cost advanced hydrogels of calcium alginate/carbon nanofibers with enhancedwater diffusion and compression properties. Polymers.

[249] Sanmartín-Santos I., Gandía-Llop S., Salesa B., Martí M., Lillelund Aachmann F., Serrano-Aroca Á. (2021). Enhancement of antimicrobial activity of alginate films with a low amount of carbon nanofibers (0.1% w/w). Appl. Sci..

[250] Salesa B., Martí M., Frígols B., Serrano-Aroca Á. (2019). Carbon nanofibers in pure form and in calcium alginate composites films: new cost-effective antibacterial biomaterials against the life-threatening multidrug-resistant Staphylococcus epidermidis. Polymers.

[251] Martí M., Frígols B., Salesa B., Serrano-Aroca Á. (2019). Calcium alginate/graphene oxide films: reinforced composites able to prevent Staphylococcus aureus and methicillin-resistant Staphylococcus epidermidis infections with no cytotoxicity for human keratinocyte HaCaT cells. Eur. Polym. J..

[252] Frígols B., Martí M., Salesa B., Hernández-Oliver C., Aarstad O., Teialeret Ulset A.S., Sӕtrom G.I., Lillelund Aachmann F., Serrano-Aroca A. (2019).

[253] Salesa B., Llorens-Gámez M., Serrano-Aroca Á. (2020). Study of 1D and 2D carbon nanomaterial in alginate films. Nanomaterials.

[254] Serrano-Aroca Á., Iskandar L., Deb S. (2018). Green synthetic routes to alginate-graphene oxide composite hydrogels with enhanced physical properties for bioengineering applications. Eur. Polym. J..

[255] Serrano-Aroca Á., Deb S. (2017). Synthesis of irregular graphene oxide tubes using green chemistry and their potential use as reinforcement materials for biomedical applications. PLoS One.

[256] Jodati H., Yilmaz B., Evis Z. (2021). In vitro and in vivo properties of graphene-incorporated scaffolds for bone defect repair. Ceram. Int..

[257] Azizi-Lalabadi M., Hashemi H., Feng J., Jafari S.M. (2020). Carbon nanomaterials against pathogens; the antimicrobial activity of carbon nanotubes, graphene/graphene oxide, fullerenes, and their nanocomposites. Adv. Colloid Interface Sci..

[258] Qiao Y., Zhai Z., Chen L., Liu H. (2015). Cytocompatible 3D chitosan/hydroxyapatite composites endowed with antibacterial properties: toward a self-sterilized bone tissue engineering scaffold. Sci. Bull..

[259] Ryu J.H., Kwon J.S., Kim K.M., Hong H.J., Koh W.G., Lee J., Lee H.J., Choi H.J., Yi S., Shin H., Hong M.H. (2019). Synergistic effect of porous hydroxyapatite scaffolds combined with bioactive glass/poly(lactic- co-glycolic acid) composite fibers promotes osteogenic activity and bioactivity. ACS Omega.

[260] Rahaman M.N., Day D.E., Sonny Bal B., Fu Q., Jung S.B., Bonewald L.F., Tomsia A.P. (2011). Bioactive glass in tissue engineering. Acta Biomater..

[261] Khurana A., Tekula S., Saifi M.A., Venkatesh P., Godugu C. (2019). Therapeutic applications of selenium nanoparticles. Biomed. Pharmacother..

[262] Li Y., Lin Z., Gong G., Guo M., Xu T., Wang C., Zhao M., Xia Y., Tang Y., Zhong J., Chen Y., Hua L., Huang Y., Zeng F., Zhu B. (2019). Inhibition of H1N1 influenza virus-induced apoptosis by selenium nanoparticles functionalized with arbidol through ROS-mediated signaling pathways. J. Mater. Chem. B..

[263] Jones J.R., Ehrenfried L.M., Saravanapavan P., Hench L.L. (2006). Controlling ion release from bioactive glass foam scaffolds with antibacterial properties. J. Mater. Sci. Mater. Med., J Mater Sci Mater Med.

[264] Agarwal T., Kazemi S., Costantini M., Perfeito F., Correia C.R., Gaspar V., Montazeri L., De Maria C., Mano J.F., Vosough M., Makvandi P., Maiti T.K. (2021). Oxygen releasing materials: towards addressing the hypoxia-related issues in tissue engineering. Mater. Sci. Eng. C..

[265] Ren L., Lin X., Tan L., Yang K. (2011). Effect of surface coating on antibacterial behavior of magnesium based metals. Mater. Lett..

[266] Salesa B., Serra R.S.I., Serrano-Aroca Á. (2021). Zinc chloride: time-dependent cytotoxicity, proliferation and promotion of glycoprotein synthesis and antioxidant gene expression in human keratinocytes. Biology.

[267] Gentile P., Chiono V., Carmagnola I., Hatton P.V. (2014). An overview of poly(lactic-co-glycolic) Acid (PLGA)-based biomaterials for bone tissue engineering. Int. J. Mol. Sci..

[268] Skidmore S., Hadar J., Garner J., Park H., Park K., Wang Y., Jeff X.(, Jiang (2019). Complex sameness: separation of mixed poly(lactide-co-glycolide)s based on the lactide:glycolide ratio. J. Contr. Release.

[269] Nair M.B., Kretlow J.D., Mikos A.G., Kasper F.K. (2011). Infection and tissue engineering in segmental bone defects-a mini review. Curr. Opin. Biotechnol..

[270] Ritz U., Gerke R., Götz H., Stein S., Rommens P.M. (2017). A new bone substitute developed from 3D-prints of polylactide (PLA) loaded with collagen i: an in vitro study. Int. J. Mol. Sci..

[271] Campoccia D., Montanaro L., Arciola C.R. (2006). The significance of infection related to orthopedic devices and issues of antibiotic resistance. Biomaterials.

[272] Akkineni A.R., Spangenberg J., Geissler M., Reichelt S., Buechner H., Lode A., Gelinsky M. (2021). Controlled and local delivery of antibiotics by 3d core/shell printed hydrogel scaffolds to treat soft tissue infections. Pharmaceutics.

[273] Dzikowski M., Castanié N., Guedon A., Verrier B., Primard C., Sohier J. (2017). Antibiotic incorporation in jet-sprayed nanofibrillar biodegradable scaffolds for wound healing. Int. J. Pharm..

[274] Buck E., Maisuria V., Tufenkji N., Cerruti M. (2018). Antibacterial properties of PLGA electrospun scaffolds containing ciprofloxacin incorporated by blending or physisorption. ACS Appl. Bio Mater..

[275] Iga C., Agata T., Marcin Ł., Natalia F., Justyna K.L. (2020). Ciprofloxacin-modified degradable hybrid polyurethane-polylactide porous scaffolds developed for potential use as an antibacterial scaffold for regeneration of skin. Polymers.

[276] Cai D., Chen S., Wu B., Chen J., Tao D., Li Z., Dong Q., Zou Y., Chen Y., Bi C., Zu D., Lu L., Fang B. (2021). Construction of multifunctional porcine acellular dermal matrix hydrogel blended with vancomycin for hemorrhage control, antibacterial action, and tissue repair in infected trauma wounds. Mater. Today Bio..

[277] Mohd Razali N.A., Lin W.-C. (2022). Accelerating the excisional wound closure by using the patterned microstructural nanofibrous mats/gentamicin-loaded hydrogel composite scaffold. Mater. Today Bio.

[278] Wang J., Zhu Y., Bawa H.K., Ng G., Wu Y., Libera M., Van Der Mei H.C., Busscher H.J., Yu X. (2011). Oxygen-generating nanofiber cell scaffolds with antimicrobial properties. ACS Appl. Mater. Interfaces.

[279] Raghavendra G.M., Jayaramudu T., Varaprasad K., Sadiku R., Ray S.S., Mohana Raju K. (2013). Cellulose-polymer-Ag nanocomposite fibers for antibacterial fabrics/skin scaffolds. Carbohydr. Polym..

[280] Lim M.M., Sultana N. (2016). In vitro cytotoxicity and antibacterial activity of silver-coated electrospun polycaprolactone/gelatine nanofibrous scaffolds. 3 Biotech.

[281] Ai F., Liu T., Liu Y., Yang K., Liu Y., Wang W., Yuan F., Dong L., Xin H., Wang X. (2018). A 3D printed wound cooling system incorporated with injectable, adsorbable, swellable and broad spectrum antibacterial scaffolds for rapid hematischesis processing. J. Mater. Chem. B..

[282] Pérez-Díaz M.A., Silva-Bermudez P., Jiménez-López B., Martínez-López V., Melgarejo-Ramírez Y., Brena-Molina A., Ibarra C., Baeza I., Martínez-Pardo M.E., Reyes-Frías M.L., Márquez-Gutiérrez E., Velasquillo C., Martínez-Castañon G., Martinez-Gutierrez F., Sánchez-Sánchez R. (2018). Silver-pig skin nanocomposites and mesenchymal stem cells: suitable antibiofilm cellular dressings for wound healing. J. Nanobiotechnol..

[283] Zheng K., Balasubramanian P., Paterson T.E., Stein R., MacNeil S., Fiorilli S., Vitale-Brovarone C., Shepherd J., Boccaccini A.R. (2019). Ag modified mesoporous bioactive glass nanoparticles for enhanced antibacterial activity in 3D infected skin model. Mater. Sci. Eng. C..

[284] Aktürk A., Erol Taygun M., Karbancıoğlu Güler F., Goller G., Küçükbayrak S. (2019). Fabrication of antibacterial polyvinylalcohol nanocomposite mats with soluble starch coated silver nanoparticles. Colloids Surfaces A Physicochem. Eng. Asp..

[285] Demir D., Ceylan S., Gül G., İyigündoğdu Z., Bölgen N. (2019). Green synthesized silver nanoparticles loaded PVA/Starch cryogel scaffolds with antibacterial properties. Teh. Glas..

[286] Abudula T., Gauthaman K., Hammad A.H., Navare K.J., Alshahrie A.A., Bencherif S.A., Tamayol A., Memic A. (2020). Oxygen-releasing antibacterial nanofibrous scaffolds for tissue engineering applications. Polymers.

[287] Nejaddehbashi F., Hashemitabar M., Bayati V., Moghimipour E., Movaffagh J., Orazizadeh M., Abbaspour M. (2020). Incorporation of silver sulfadiazine into an electrospun composite of polycaprolactone as an antibacterial scaffold for wound healing in rats. Cell J.

[288] Oliveira R.L.M.S., Barbosa L., Hurtado C.R., Ramos L. de P., Montanheiro T.L.A., Oliveira L.D., Tada D.B., Trichês E. de S. (2020). Bioglass-based scaffolds coated with silver nanoparticles: synthesis, processing and antimicrobial activity. J. Biomed. Mater. Res., Part A.

[289] Li Y., Xu T., Tu Z., Dai W., Xue Y., Tang C., Gao W., Mao C., Lei B., Lin C. (2020). Bioactive antibacterial silica-based nanocomposites hydrogel scaffolds with high angiogenesis for promoting diabetic wound healing and skin repair. Theranostics.

[290] Ahmed M.K., Zayed M.A., El-dek S.I., Hady M.A., El Sherbiny D.H., Uskoković V. (2021). Nanofibrous ε-polycaprolactone scaffolds containing Ag-doped magnetite nanoparticles: physicochemical characterization and biological testing for wound dressing applications in vitro and in vivo. Bioact. Mater..

[291] Eivazzadeh-Keihan R., Moghim Aliabadi H.A., Radinekiyan F., Sobhani M., Khalili Farzane, Maleki A., Madanchi H., Mahdavi M., Shalan A.E. (2021). Investigation of the biological activity, mechanical properties and wound healing application of a novel scaffold based on lignin-agarose hydrogel and silk fibroin embedded zinc chromite nanoparticles. RSC Adv..

[292] Tao B., Lin C., Qin X., Yu Y., Guo A., Li K., Tian H., Yi W., Lei D., Chen Y., Chen L. (2022). Fabrication of gelatin-based and Zn2+-incorporated composite hydrogel for accelerated infected wound healing, Mater. Today Bio.

[293] Fang Y., Xu Y., Wang Z., Zhou W., Yan L., Fan X., Liu H. (2020). 3D porous chitin sponge with high absorbency, rapid shape recovery, and excellent antibacterial activities for noncompressible wound. Chem. Eng. J..

[294] Tardajos M.G., Cama G., Dash M., Misseeuw L., Gheysens T., Gorzelanny C., Coenye T., Dubruel P. (2018). Chitosan functionalized poly-ε-caprolactone electrospun fibers and 3D printed scaffolds as antibacterial materials for tissue engineering applications. Carbohydr. Polym..

[295] Piątkowski M., Kitala D., Radwan-Pragłowska J., Janus, Klama-Baryła A., Łabuś W., Tomanek E., Glik J., Matýsek D., Kawecki M. (2018). Chitosan/aminoacid hydrogels with antimicrobial and bioactive properties as new scaffolds for human mesenchymal stem cells culture applicable in wound healing. Express Polym. Lett..

[296] Xie X., Li D., Su C., Cong W., Mo X., Hou G., Wang C. (2019). Functionalized biomimetic composite nanfibrous scaffolds with antibacterial and hemostatic efficacy for facilitating wound healing. J. Biomed. Nanotechnol..

[297] Goller S., Turner N.J. (2020). The antimicrobial effectiveness and cytotoxicity of the antibiotic-loaded chitosan: ecm scaffolds. Appl. Sci..

[298] Bou Haidar N., Marais S., Dé E., Schaumann A., Barreau M., Feuilloley M.G.J., Duncan A.C. (2020). Chronic wound healing: a specific antibiofilm protein-asymmetric release system. Mater. Sci. Eng. C..

[299] Kandhasamy S., Liang B., Yang D.P., Zeng Y. (2021). Antibacterial Vitamin K3 carnosine peptide-laden silk fibroin electrospun fibers for improvement of skin wound healing in diabetic rats. ACS Appl. Bio Mater..

[300] Chizari M., Khosravimelal S., Tebyaniyan H., Moosazadeh Moghaddam M., Gholipourmalekabadi M. (2022). Fabrication of an antimicrobial peptide-loaded silk fibroin/gelatin bilayer sponge to apply as a wound dressing; an in vitro study. Int. J. Pept. Res. Therapeut..

[301] Zine R., Sinha M. (2017). Nanofibrous poly(3-hydroxybutyrate-co-3-hydroxyvalerate)/collagen/graphene oxide scaffolds for wound coverage.

[302] Thangavel P., Kannan R., Ramachandran B., Moorthy G., Suguna L., Muthuvijayan V. (2018). Development of reduced graphene oxide (rGO)-isabgol nanocomposite dressings for enhanced vascularization and accelerated wound healing in normal and diabetic rats. J. Colloid Interface Sci..

[303] Jian Z., Wang H., Liu M., Chen S., Wang Z., Qian W., Luo G., Xia H. (2020). Polyurethane-modified graphene oxide composite bilayer wound dressing with long-lasting antibacterial effect. Mater. Sci. Eng. C..

[304] Zmejkoski D.Z., Marković Z.M., Mitić D.D., Zdravković N.M., Kozyrovska N.O., Bugárová N., Todorović Marković B.M. (2022). Antibacterial composite hydrogels of graphene quantum dots and bacterial cellulose accelerate wound healing. J. Biomed. Mater. Res. Part B Appl. Biomater..

[305] Hurtado A., Cano-Vicent A., Tuñón-Molina A., Aparicio-Collado J.L., Salesa B., i Serra R.S., Serrano-Aroca Á. (2022). Engineering alginate hydrogel films with poly(3-hydroxybutyrate-co-3-valerate) and graphene nanoplatelets: enhancement of antiviral activity, cell adhesion and electroactive properties. Int. J. Biol. Macromol..

[306] Xing Z.C., Meng W., Yuan J., Moon S., Jeong Y., Kang I.K. (2012). In vitro assessment of antibacterial activity and cytocompatibility of quercetin-containing PLGA nanofibrous scaffolds for tissue engineering. J. Nanomater..

[307] Sarhan W.A., Awad W. (2016).

[308] Biswas D.P., O'Brien-Simpson N.M., Reynolds E.C., O'Connor A.J., Tran P.A. (2018). Comparative study of novel in situ decorated porous chitosan-selenium scaffolds and porous chitosan-silver scaffolds towards antimicrobial wound dressing application. J. Colloid Interface Sci..

[309] Marei N.H. (2018). Development of chitosan 2D film scaffolds and nanoparticles enriched with royal jelly and grape seed extract: Enhanced antibacterial and wound healing activity.

[310] Mukheem A., Muthoosamy K., Manickam S., Sudesh K., Shahabuddin S., Saidur R., Akbar N., Sridewi N. (2018). Fabrication and characterization of an electrospun PHA/graphene silver nanocomposite scaffold for antibacterial applications. Materials.

[311] Çakır C.O., Ozturk M.T., Tuzlakoglu K. (2018). Design of antibacterial bilayered silk fibroin-based scaffolds for healing of severe skin damages. Mater. Technol..

[312] Dhand C., Balakrishnan Y., Ong S.T., Dwivedi N., Venugopal J.R., Harini S., Leung C.M., Low K.Z.W., Loh X.J., Beuerman R.W., Ramakrishna S., Verma N.K., Lakshminarayanan R. (2018). Antimicrobial quaternary ammonium organosilane cross-linked nanofibrous collagen scaffolds for tissue engineering. Int. J. Nanomed..

[313] Adeli-Sardou M., Torkzadeh-Mahani M., Yaghoobi M.M., Dodel M. (2018). Biomacromolecular journal antibacterial and anti-biofilm investigation of electrospun PCL/gelatin/Lawsone nano fiber scaffolds against biofilm producing bacteria. Biomacromolecules J..

[314] De Silva R.T., Dissanayake R.K., Wijesinghe W.P.S.L., Kaleel S.S., Premachandra T.N., Weerasinghe L., Amaratunga G.A.J., De Silva K.M.N., M.M.M.G.P.G. Mantilaka (2018). Drug-loaded halloysite nanotube-reinforced electrospun alginate-based nanofibrous scaffolds with sustained antimicrobial protection. ACS Appl. Mater. Interfaces.

[315] Hixon K.R., Bogner S.J., Ronning-Arnesen G., Janowiak B.E., Sell S.A. (2019). Investigating manuka honey antibacterial properties when incorporated into cryogel, hydrogel, and electrospun tissue engineering scaffolds. Gels.

[316] Gomaa S.F., Madkour T.M., Moghannem S., El-Sherbiny I.M. (2017).

[317] Popelka A., Abdulkareem A., Mahmoud A.A., Nassr M.G., Al-Ruweidi M.K.A.A., Mohamoud K.J., Hussein M.K., Lehocky M., Vesela D., Humpolíček P., Kasak P. (2020). Antimicrobial modification of PLA scaffolds with ascorbic and fumaric acids via plasma treatment. Surf. Coating. Technol..

[318] Fan S., Chen K., Yuan W., Zhang D., Yang S., Lan P., Song L., Shao H., Zhang Y. (2020). Biomaterial-based scaffolds as antibacterial suture materials. ACS Biomater. Sci. Eng..

[319] Demir D., Özdemir S., Yalçın M.S., Bölgen N. (2020). Chitosan cryogel microspheres decorated with silver nanoparticles as injectable and antimicrobial scaffolds. Int. J. Polym. Mater. Polym. Biomater..

[320] Tripathi S., Singh B.N., Singh D., kumar G., Srivastava P. (2021). Optimization and evaluation of ciprofloxacin-loaded collagen/chitosan scaffolds for skin tissue engineering. 3 Biotech.

[321] Tripathi S., Singh B.N., Divakar S., Kumar G., Mallick S.P., Srivastava P. (2021). Design and evaluation of ciprofloxacin loaded collagen chitosan oxygenating scaffold for skin tissue engineering. Biomed. Mater..

[322] Parandhaman T., Choudhary P., Ramalingam B., Schmidt M., Janardhanam S., Das S.K. (2021). Antibacterial and antibiofouling activities of antimicrobial peptide-functionalized graphene-silver nanocomposites for the inhibition and disruption of Staphylococcus aureus biofilms. ACS Biomater. Sci. Eng..

[323] Slate A.J., Karaky N., Crowther G.S., Butler J.A., Banks C.E., McBain A.J., Whitehead K.A. (2021). Graphene matrices as carriers for metal ions against antibiotic susceptible and resistant bacterial pathogens. Coatings.

[324] Nazir S., Umar Aslam Khan M., Shamsan Al-Arjan W., Izwan Abd Razak S., Javed A., Rafiq Abdul Kadir M. (2021). Nanocomposite hydrogels for melanoma skin cancer care and treatment: in-vitro drug delivery, drug release kinetics and anti-cancer activities. Arab. J. Chem..

[325] Khan M.U.A., Iqbal I., Ansari M.N.M., Razak S.I.A., Raza M.A., Sajjad A., Jabeen F., Mohamad M.R., Jusoh N. (2021). Development of antibacterial, degradable and ph-responsive chitosan/guar gum/polyvinyl alcohol blended hydrogels for wound dressing. Molecules.

[326] Khan M.U.A., Yaqoob Z., Nainar M.M.A., Razak S.I.A., Raza M.A., Sajjad A., Haider S., Busra F.M. (2021). Chitosan/poly vinyl alcohol/graphene oxide based ph-responsive composite hydrogel films: drug release, anti-microbial and cell viability studies. Polymers.

[327] Khan M.U.A., Haider S., Raza M.A., Shah S.A., Razak S.I.A., Kadir M.R.A., Subhan F., Haider A. (2021). Smart and pH-sensitive rGO/Arabinoxylan/chitosan composite for wound dressing: in-vitro drug delivery, antibacterial activity, and biological activities. Int. J. Biol. Macromol..

[328] Khan M.U.A., Abd Razaq S.I., Mehboob H., Rehman S., Al-Arjan W.S., Amin R. (2021). Antibacterial and hemocompatible ph-responsive hydrogel for skin wound healing application: in vitro drug release. Polymers.

[329] Khan M.U.A., Razak S.I.A., Hassan A., Qureshi S., Stojanović G.M., Ihsan-Ul-Haq (2022). Multifunctional arabinoxylan-functionalized-graphene oxide based composite hydrogel for skin tissue engineering. Front. Bioeng. Biotechnol..

[330] Al-Arjan W.S., Khan M.U.A., Almutairi H.H., Alharbi S.M., Razak S.I.A. (2022). pH-responsive PVA/BC-f-GO dressing materials for burn and chronic wound healing with curcumin release kinetics. Polymers.

[331] Khan M.U.A., Razak S.I.A., Haider S., Mannan H.A., Hussain J., Hasan A. (2022). Sodium alginate-f-GO composite hydrogels for tissue regeneration and antitumor applications. Int. J. Biol. Macromol..

[332] Li Z.P., You S., Mao R., Xiang Y., Cai E., Deng H., Shen J., Qi X. (2022). Architecting polyelectrolyte hydrogels with Cu-assisted polydopamine nanoparticles for photothermal antibacterial therapy, Mater. Today Bio.

[333] Gritsch L., Boccaccini A.R. (2017). Antimicrobial chitosan foams with and without polyester blending as tissue engineering scaffolds. Syntactic Compos. Foam. V..

[334] Mohandas A., Deepthi S., Biswas R., Jayakumar R. (2018). Chitosan based metallic nanocomposite scaffolds as antimicrobial wound dressings. Bioact. Mater..

[335] Tiwari S., Patil R., Dubey S.K., Bahadur P. (2020). Graphene nanosheets as reinforcement and cell-instructive material in soft tissue scaffolds. Adv. Colloid Interface Sci..

[336] Kucinska-Lipka J., Gubanska I., Janik H., Pokrywczynska M., Drewa T. (2015). L-ascorbic acid modified poly(ester urethane)s as a suitable candidates for soft tissue engineering applications. React. Funct. Polym..

[337] Chung S., Webster T.J. (2016). Adv. Polyurethane Biomater..

[338] Zheng J.P., Li C.Z., Chen G.Q., Song G.D., Zhang Y.Z. (2018). Three-dimensional printed skull base simulation for transnasal endoscopic surgical training. World Neurosurg.

[339] Jeppson J. (2012).

[340] Palasuk J., Kamocki K., Hippenmeyer L., Platt J.A., Spolnik K.J., Gregory R.L., Bottino M.C. (2014). Bimix antimicrobial scaffolds for regenerative endodontics. J. Endod..

[341] Kamocki K., Nör J.E., Bottino M.C. (2015). Effects of ciprofloxacin-containing antimicrobial scaffolds on dental pulp stem cell viability - in vitro studies. Arch. Oral Biol..

[342] Albuquerque M.T.P., Evans J.D., Gregory R.L., Valera M.C., Bottino M.C. (2016). Antibacterial TAP-mimic electrospun polymer scaffold: effects on P. gingivalis-infected dentin biofilm. Clin. Oral Invest..

[343] Kim D.H., Son J.S., Kwon T.Y. (2020). Antimicrobial effect of chlorhexidine-releasing porous hydroxyapatite scaffold incorporated with human serum albumin nanoparticles. Mater. Lett..

[344] Da Wu H., Ji D.Y., Chang W.J., Yang J.C., Lee S.Y. (2012). Chitosan-based polyelectrolyte complex scaffolds with antibacterial properties for treating dental bone defects. Mater. Sci. Eng. C..

[345] Shao J., Ma J., Lin L., Wang B., Jansen J.A., Walboomers X.F., Zuo Y., Yang F. (2019). Three-dimensional printing of drug-loaded scaffolds for antibacterial and analgesic applications. Tissue Eng. C Methods.

[346] Marin E., Boschetto F., Sunthar T.P.M., Zanocco M., Ohgitani E., Zhu W., Pezzotti G. (2020). Antibacterial effects of barium titanate reinforced polyvinyl-siloxane scaffolds. Int. J. Polym. Mater. Polym. Biomater..

[347] Craciunescu O., Seciu A.M., Zarnescu O. (2021). In vitro and in vivo evaluation of a biomimetic scaffold embedding silver nanoparticles for improved treatment of oral lesions. Mater. Sci. Eng. C..

[348] El-Sayed S.A.M., Mabrouk M., Khallaf M.E., Abd El-Hady B.M., El-Meliegy E., Shehata M.R. (2020). Antibacterial, drug delivery, and osteoinduction abilities of bioglass/chitosan scaffolds for dental applications. J. Drug Deliv. Sci. Technol..

[349] Esfahanizadeh N., Nourani M.R., Bahador A., Akhondi N., Montazeri M. (2020). The anti-biofilm activity of nanometric zinc doped bioactive glass against putative periodontal pathogens: an in vitro study. Biomed. Glas..

[350] Kwon Y.S., Kim H.J., Hwang Y.C., Rosa V., Yu M.K., Min K.S. (2017). Effects of epigallocatechin gallate, an antibacterial cross-linking agent, on proliferation and differentiation of human dental pulp cells cultured in collagen scaffolds. J. Endod..

[351] Wu S., Weir M.D., Lei L., Liu J., Xu H.H.K. (2021). Novel nanographene oxide-calcium phosphate cement inhibits Enterococcus faecalis biofilm and supports dental pulp stem cells. J. Orthop. Surg. Res..

[352] Sairaman S., Nivedhitha M.S., Shrivastava D., Al Onazi M.A., Algarni H.A., Mustafa M., Alqahtani A.R., AlQahtani N., Teja K.V., Janani K., Eswaramoorthy R., Sudhakar M.P., Alam M.K., Srivastava K.C. (2022). Biocompatibility and antioxidant activity of a novel carrageenan based injectable hydrogel scaffold incorporated with Cissus quadrangularis for facilitating dentin-pulp complex regeneration – an in vitro study. ResearchSquare.

[353] Barua S., Gogoi B., Aidew L., Buragohain A.K., Chattopadhyay P., Karak N. (2015). Sustainable resource based hyperbranched epoxy nanocomposite as an infection resistant, biodegradable, implantable muscle scaffold. ACS Sustain. Chem. Eng..

[354] Zhao X., Li P., Guo B., Ma P.X. (2015). Antibacterial and conductive injectable hydrogels based on quaternized chitosan-graft-polyaniline/oxidized dextran for tissue engineering. Acta Biomater..

[355] Kang Y., Wang C., Qiao Y., Gu J., Zhang H., Peijs T., Kong J., Zhang G., Shi X. (2019). Tissue-engineered trachea consisting of electrospun patterned sc-PLA/GO- g-IL fibrous membranes with antibacterial property and 3D-printed skeletons with elasticity. Biomacromolecules.

[356] Vahedi P., Moghaddamshahabi R., Webster T.J., Koyuncu A.C.C., Ahmadian E., Khan W.S., Mohamed A.J., Eftekhari A. (2021). The use of infrapatellar fat pad-derived Mesenchymal stem cells in Articular cartilage regeneration: a review. Int. J. Mol. Sci..

[357] Godoy-Gallardo M., Eckhard U., Delgado L.M., de Roo Puente Y.J.D., Hoyos-Nogués M., Gil F.J., Perez R.A. (2021). Antibacterial approaches in tissue engineering using metal ions and nanoparticles: from mechanisms to applications. Bioact. Mater..

[358] Chen J., Zhou B., Li Q., Ouyang J., Kong J., Zhong W., Xing M.M.Q. (2011). PLLA-PEG-TCH-labeled bioactive molecule nanofibers for tissue engineering. Int. J. Nanomed..

[359] Visscher L.E., Dang H.P., Knackstedt M.A., Hutmacher D.W., Tran P.A. (2018). 3D printed Polycaprolactone scaffolds with dual macro-microporosity for applications in local delivery of antibiotics. Mater. Sci. Eng. C..

[360] Kumari S., Bargel H., Scheibel T. (2020). Recombinant spider silk–silica hybrid scaffolds with drug-releasing properties for tissue engineering applications, macromol. Rapid Commun.

[361] Nguyen T.T.T., Tae B., Park J.S. (2011). Synthesis and characterization of nanofiber webs of chitosan/poly(vinyl alcohol) blends incorporated with silver nanoparticles. J. Mater. Sci..

[362] Muñoz L., Tamayo L., Gulppi M., Rabagliati F., Flores M., Urzúa M., Azócar M., Zagal J.H., Encinas M.V., Zhou X., Thompson G., Páez M. (2018). Surface functionalization of an aluminum alloy to generate an antibiofilm coating based on poly(methyl methacrylate) and silver nanoparticles. Molecules.

[363] Qing Y., Li K., Li D., Qin Y. (2020). Antibacterial effects of silver incorporated zeolite coatings on 3D printed porous stainless steels. Mater. Sci. Eng. C..

[364] Abdallah O.M., EL-Baghdady K.Z., Khalil M.M.H., El Borhamy M.I., Meligi G.A. (2020). Antibacterial, antibiofilm and cytotoxic activities of biogenic polyvinyl alcohol-silver and chitosan-silver nanocomposites. J. Polym. Res..

[365] Ge J., Li Y., Wang M., Gao C., Yang S., Lei B. (2021). Engineering conductive antioxidative antibacterial nanocomposite hydrogel scaffolds with oriented channels promotes structure-functional skeletal muscle regeneration. Chem. Eng. J..

[366] Mortazavi V., Mehdikhani Nahrkhalaji M., Fathi M.H., Mousavi S.B., Nasr Esfahani B. (2010). Antibacterial effects of sol-gel-derived bioactive glass nanoparticle on aerobic bacteria. J. Biomed. Mater. Res., Part A.

[367] Tang Y., Zhao Y., Wang H., Gao Y., Liu X., Wang X., Lin T. (2012). Layer-by-layer assembly of antibacterial coating on interbonded 3D fibrous scaffolds and its cytocompatibility assessment. J. Biomed. Mater. Res., Part A.

[368] Dong R., Zhao X., Guo B., Ma P.X. (2016). Self-healing conductive injectable hydrogels with antibacterial activity as cell delivery carrier for cardiac cell therapy. ACS Appl. Mater. Interfaces.

[369] Vargas-Alfredo N., Dorronsoro A., Cortajarena A.L., Rodríguez-Hernández J. (2017). Antimicrobial 3D porous scaffolds prepared by additive manufacturing and breath figures. ACS Appl. Mater. Interfaces.

[370] Ghannadian P., Moxley J.W., MacHado De Paula M.M., Lobo A.O., Webster T.J. (2018). Micro-nanofibrillar polycaprolactone scaffolds as translatable osteoconductive grafts for the treatment of musculoskeletal defects without infection. ACS Appl. Bio Mater..

[371] Murugesan B., Sonamuthu J., Samayanan S., Arumugam S., Mahalingam S. (2018). Highly biological active antibiofilm, anticancer and osteoblast adhesion efficacy from MWCNT/PPy/Pd nanocomposite. Appl. Surf. Sci..

[372] Ahonen M.J.R., Dorrier J.M., Schoenfisch M.H. (2019). Antibiofilm efficacy of nitric oxide-releasing alginates against cystic fibrosis bacterial pathogens. ACS Infect. Dis..

[373] Chakraborty P., Oved H., Bychenko D., Yao Y., Tang Y., Zilberzwige-Tal S., Wei G., Dvir T., Gazit E. (2021). Nanoengineered peptide-based antimicrobial conductive supramolecular biomaterial for cardiac tissue engineering. Adv. Mater.

[374] Norahan M.H., Pourmokhtari M., Saeb M.R., Bakhshi B., Soufi Zomorrod M., Baheiraei N. (2019). Electroactive cardiac patch containing reduced graphene oxide with potential antibacterial properties. Mater. Sci. Eng. C..

[375] Murugesan B., Pandiyan N., Arumugam M., Sonamuthu J., Samayanan S., Yurong C., Juming Y., Mahalingam S. (2020). Fabrication of palladium nanoparticles anchored polypyrrole functionalized reduced graphene oxide nanocomposite for antibiofilm associated orthopedic tissue engineering. Appl. Surf. Sci..

[376] Zuo M., Pan N., Liu Q., Ren X., Liu Y., Huang T.S. (2020). Three-dimensionally printed polylactic acid/cellulose acetate scaffolds with antimicrobial effect. RSC Adv..

[377] Amna T., Alghamdi A.A.A., Shang K., Hassan M.S. (2021). Nigella sativa-coated hydroxyapatite scaffolds: synergetic cues to stimulate myoblasts differentiation and offset infections. Tissue Eng. Regen. Med..

[378] Yin J., Zhong J., Wang J., Wang Y., Li T., Wang L., Yang Y., Zhen Z., Li Y., Zhang H., Zhong S., Wu Y., Huang W. (2022). 3D-printed high-density polyethylene scaffolds with bioactive and antibacterial layer-by-layer modification for auricle reconstruction. Mater. Today Bio..

[379] Zhao X., Ma H., Han H., Zhang L., Tian J., Lei B., Zhang Y. (2022). Precision medicine strategies for spinal degenerative diseases: injectable biomaterials with in situ repair and regeneration, Mater. Today Bio.

[380] Serrano-Aroca Á., Takayama K., Tuñón-Molina A., Seyran M., Hassan S.S., Pal Choudhury P., Uversky V.N., Lundstrom K., Adadi P., Palù G., Aljabali A.A.A., Chauhan G., Kandimalla R., Tambuwala M.M., Lal A., Abd El-Aziz T.M., Sherchan S., Barh D., Redwan E.M., Bazan N.G., Mishra Y.K., Uhal B.D., Brufsky A. (2021). Carbon-based nanomaterials: promising antiviral agents to combat COVID-19 in the microbial-resistant era. ACS Nano.

[381] Wang S.Z., Sheng C.Q., Zhang W.N. (2010). Recent advances in the study of new antifungal lead compounds. Yaoxue Xuebao.

[382] Liu N., Wang C., Su H., Zhang W., Sheng C. (2016). Strategies in the discovery of novel antifungal scaffolds. Future Med. Chem..

[383] Demirci T., Hasköylü M.E., Eroğlu M.S., Hemberger J., Toksoy Öner E. (2020). Levan-based hydrogels for controlled release of Amphotericin B for dermal local antifungal therapy of Candidiasis. Eur. J. Pharmaceut. Sci..

[384] Karimi S., Moradipour P., Azandaryani A.H., Arkan E. (2019). Amphotericin-B and vancomycin-loaded chitosan nanofiber for antifungal and antibacterial application. Brazilian J. Pharm. Sci..

[385] Pekmezovic M., Krusic M.K., Malagurski I., Milovanovic J., Stępień K., Guzik M., Charifou R., Babu R., O’connor K., Nikodinovic-Runic J. (2021). Polyhydroxyalkanoate/antifungal polyene formulations with monomeric hydroxyalkanoic acids for improved antifungal efficiency. Antibiotics.

[386] Bakadia B.M., Lamboni L., Qaed Ahmed A.A., Zheng R., Ode Boni B.O., Shi Z., Song S., Souho T., Mukole B.M., Qi F., Yang G. (2023). Antibacterial silk sericin/poly (vinyl alcohol) hydrogel with antifungal property for potential infected large burn wound healing: systemic evaluation. Smart Mater. Med..

[387] Hipler U.C., Elsner P., Fluhr J.W. (2006). Antifungal and antibacterial properties of a silver-loaded cellulosic fiber. J. Biomed. Mater. Res. Part B Appl. Biomater..

[388] Vlad S., Tanase C., Macocinschi D., Ciobanu C., Balaes T., Filip D., Gostin I.N., Gradinaru L.M. (2012). Antifungal behaviour of polyurethane membranes with zinc oxide nanoparticles. Dig. J. Nanomater. Biostructures..

[389] Eraković S., Janković A., Ristoscu C., Duta L., Serban N., Visan A., Mihailescu I.N., Stan G.E., Socol M., Iordache O., Dumitrescu I., Luculescu C.R., Janaćković D., Miškovic-Stanković V. (2014). Antifungal activity of Ag:hydroxyapatite thin films synthesized by pulsed laser deposition on Ti and Ti modified by TiO 2 nanotubes substrates. Appl. Surf. Sci..

[390] Yazdimamaghani M., Vashaee D., Assefa S., Shabrangharehdasht M., Rad A.T., Eastman M.A., Walker K.J., Madihally S.V., Köhler G.A., Tayebi L. (2014). Green synthesis of a new gelatin-based antimicrobial scaffold for tissue engineering. Mater. Sci. Eng. C..

[391] Ouis M.A., Fayad A.A., Abd El Aty A.A., El-Bassyouni G.T. (2018). Processing, characterization and application of some borophosphate glasses containing antibacterial and antifungaloxides in bioactive demands, Egypt. J. Chem..

[392] Trcin M.T., Zdraveva E., Dolenec T., Zimić I.V., Mihica M.B., Batarilo I., Dekaris I., Blažević V., Slivac I., Grgurić T.H., Bajsić E.G., Markov K., Čanak I., Kuzmić S., Tarbuk A., Tomljenović A., Mrkonjić N., Mijović B. (2020). Poly(ε-caprolactone) titanium dioxide and cefuroxime antimicrobial scaffolds for cultivation of human limbal stem cells. Polymers.

[393] Artunduaga Bonilla J.J., Honorato L., Cordeiro De Oliveira D.F., Araújo Gonçalves R., Guimarães A., Miranda K., Nimrichter L. (2021). 1Ilver chitosan nanocomposites as a potential treatment for superficial candidiasis. Med. Mycol..

[394] Viana J.F.C., Carrijo J., Freitas C.G., Paul A., Alcaraz J., Lacorte C.C., Migliolo L., Andrade C.A., Falcão R., Santos N.C., Gonçalves S., Otero-González A.J., Khademhosseini A., Dias S.C., Franco O.L. (2015). Antifungal nanofibers made by controlled release of sea animal derived peptide. Nanoscale.

[395] Mofidfar M., Wang J., Long L., Hager C.L., Vareechon C., Pearlman E., Baer E., Ghannoum M., Wnek G.E. (2017). Polymeric nanofiber/antifungal formulations using a novel Co-extrusion approach. AAPS PharmSciTech.

[396] Misra S.K., Ramteke P.W., Patil S., Pandey A.C., Pandey H. (2018). Tolnaftate–graphene composite-loaded nanoengineered electrospun scaffolds as efficient therapeutic dressing material for regimen of dermatomycosis. Appl. Nanosci..

[397] Esenturk I., Gumrukcu S., Buse A., Sert Ö., Kök F.N., Döşler S., Gungor S., Erdal M.S., Sarac A.S. (2020). International Journal of Polymeric Materials and Silk-fibroin-containing nanofibers for topical sertaconazole delivery : preparation , characterization , and antifungal activity. Int. J. Polym. Mater. Polym. Biomater..

[398] Mishra P., Gupta P., Srivastava A.K., Poluri K.M., Prasad R. (2021). Eucalyptol/β-cyclodextrin inclusion complex loaded gellan/PVA nanofibers as antifungal drug delivery system. Int. J. Pharm..

[399] Açarı İ.K., Boran F., Kolak S., Tatlıcı E., Yeşilada Ö., Köytepe S., Ateş B. (2022). Preparation of 10-undecenoic acid based polyurethane/PCL fibers by electrospinning method and investigation of their antifungal properties. Polym. Bull..

[400] Zahedi P., Rezaeian I., Ranaei-Siadat S.O., Jafari S.H., Supaphol P. (2010). A review on wound dressings with an emphasis on electrospun nanofibrous polymeric bandages. Polym. Adv. Technol..

[401] Lakshminarayanan R., Sridhar R., Loh X.J., Nandhakumar M., Barathi V.A., Kalaipriya M., Kwan J.L., Liu S.P., Beuerman R.W., Ramakrishna S. (2014). Interaction of gelatin with polyenes modulates antifungal activity and biocompatibility of electrospun fiber mats. Int. J. Nanomed..

[402] Mahfooz-Ur-Rehman M., Rehman W., Waseem M., Shah B.A., Shakeel M., Haq S., Zaman U., Bibi I., Khan H.D. (2019). Fabrication of titanium-tin oxide nanocomposite with enhanced adsorption and antimicrobial applications. J. Chem. Eng. Data.

[403] Otte J.M., Vordenbäumen S. (2011). Role of antimicrobial peptides in inflammatory bowel disease. Polymers.

[404] Veerachamy S., Yarlagadda T., Manivasagam G., Yarlagadda P.K. (2014). Bacterial adherence and biofilm formation on medical implants: a review. Proc. Inst. Mech. Eng. Part H J. Eng. Med..

[405] Black C.E., Costerton J.W. (2010). Current concepts regarding the effect of wound microbial ecology and biofilms on wound healing. Surg. Clin..

[406] Tarai B., Das P., Kumar D. (2013). Recurrent challenges for clinicians: emergence of methicillin-resistant Staphylococcus aureus , vancomycin resistance, and current treatment options. J. Lab. Physicians..

[407] Chhibber T., Gondil V.S., Sinha V.R. (2020). Development of chitosan-based hydrogel containing antibiofilm agents for the treatment of Staphylococcus aureus–infected burn wound in mice. AAPS PharmSciTech.

[408] Sriramulu D.D., Lünsdorf H., Lam J.S., Römling U. (2005). Microcolony formation: a novel biofilm model of Pseudomonas aeruginosa for the cystic fibrosis lung. J. Med. Microbiol..

[409] Boccaccini A.R., Erol M., Stark W.J., Mohn D., Hong Z., Mano J.F. (2010). Polymer/bioactive glass nanocomposites for biomedical applications: a review. Compos. Sci. Technol..

[410] Martí M., Frígols B., Serrano-Aroca Á. (2018). Antimicrobial characterization of advanced materials for bioengineering applications. JoVE.

[411] Alonso C.A., Domínguez C., Heras J., Mata E., Pascual V., Torres C., Zarazaga M. (2017). Antibiogramj: a tool for analysing images from disk diffusion tests. Comput. Methods Progr. Biomed..

[412] Bauer A.W., Kirby W.M.M., Sherris J.C., Turck A.M. (1966). Antibiotic susceptibility testing by a standardized single disk method. Am. J. Clin. Pathol..

[413] Stewart P.S., Costerton J.W. (2001). Antibiotic resistance of bacteria in biofilms. Lancet.

[414] Pérez-Köhler B., Sotomayor S., Rodríguez M., Gegúndez M.I., Pascual G., Bellón J.M. (2015). Bacterial adhesion to biological versus polymer prosthetic materials used in abdominal wall defect repair: do these meshes show any differences in vitro?. Hernia.

[415] Mendez E., Walker D.K., Vipham J., Trinetta V. (2020). The use of a CDC biofilm reactor to grow multi-strain Listeria monocytogenes biofilm. Food Microbiol..

[416] Fernández-Rivero M.E., Del Pozo J.L., Valentín A., de Diego A.M., Pemán J., Cantón E. (2017). Activity of amphotericin B and anidulafungin combined with rifampicin, clarithromycin, ethylenediaminetetraacetic acid, N-acetylcysteine, and farnesol against Candida tropicalis biofilms. J. Fungi..

[417] Palmer R.J., Sternberg C. (1999). Modern microscopy in biofilm research: confocal microscopy and other approaches. Curr. Opin. Biotechnol..

[418] Schlafer S., Meyer R.L. (2017). Confocal microscopy imaging of the biofilm matrix. J. Microbiol. Methods.

[419] Beech I.B., Smith J.R., Steele A.A., Penegar I., Campbell S.A. (2002). The use of atomic force microscopy for studying interactions of bacterial biofilms with surfaces. Colloids Surfaces B Biointerfaces.

[420] Chatterjee S., Biswas N., Datta A., Dey R., Maiti P. (2014). Atomic force microscopy in biofilm study. Microscopy.

[421] Ahmed W., Zhai Z., Gao C. (2019). Adaptive antibacterial biomaterial surfaces and their applications, Mater. Today Bio.

[422] Gelmi A., Schutt C.E. (2021). Stimuli-responsive biomaterials: scaffolds for stem cell control. Adv. Healthc. Mater..

[423] Ramesh S., Kovelakuntla V., Meyer A.S. (2021). Three-dimensional printing of stimuli-responsive hydrogel with antibacterial activity. Bioprinting.

[424] Oliva N., Almquist B.D. (2020). Spatiotemporal delivery of bioactive molecules for wound healing using stimuli-responsive biomaterials. Adv. Drug Deliv. Rev..

[425] Campoccia D., Montanaro L., Arciola C.R. (2013). A review of the biomaterials technologies for infection-resistant surfaces. Biomaterials.

[426] Campoccia D., Montanaro L., Arciola C.R. (2013). A review of the clinical implications of anti-infective biomaterials andinfection-resistant surfaces. Biomaterials.

[427] Ramamurthy C.H., Padma M., mariya samadanam I.D., Mareeswaran R., Suyavaran A., Kumar M.S., Premkumar K., Thirunavukkarasu C. (2013). The extra cellular synthesis of gold and silver nanoparticles and their free radical scavenging and antibacterial properties. Colloids Surfaces B Biointerfaces.

[428] Xiang Y., Liu X., Mao C., Liu X., Cui Z., Yang X., Yeung K.W.K., Zheng Y., Wu S. (2018). Infection-prevention on Ti implants by controlled drug release from folic acid/ZnO quantum dots sealed titania nanotubes. Mater. Sci. Eng. C..

[429] Cross E.R., Coulter S.M., Fuentes-Caparrós A.M., McAulay K., Schweins R., Laverty G., Adams D.J. (2020). Tuning the antimicrobial activity of low molecular weight hydrogels using dopamine autoxidation. Chem. Commun..

[430] Li M., Liu X., Xu Z., Yeung K.W.K., Wu S. (2016). Dopamine modified organic-inorganic hybrid coating for antimicrobial and osteogenesis. ACS Appl. Mater. Interfaces.

[431] He X., Obeng E., Sun X., Kwon N., Shen J., Yoon J. (2022). Polydopamine, harness of the antibacterial potentials-A review. Mater. Today Bio..

[432] Zheng L.Y., Zhu J.F. (2003). Study on antimicrobial activity of chitosan with different molecular weights. Carbohydr. Polym..

[433] Wu M., Zou L., Jiang L., Zhao Z., Liu J. (2021). Osteoinductive and antimicrobial mechanisms of graphene-based materials for enhancing bone tissue engineering. J. Tissue Eng. Regen. Med..

